# SARS-CoV-2 seeds IAPP aggregation and β-cell failure

**DOI:** 10.1038/s41392-026-02914-y

**Published:** 2026-10-09

**Authors:** Zhiqi Song, Yanfeng Xu, Wenjie Zhao, Ling Zhang, Yanhong Li, Feifei Qi, Wei Liang, Yunlin Han, Ruolin Wang, Qi Lv, Xujian Liang, Fengdi Li, Ran Deng, Wei Deng, Linlin Bao, Chuan Qin

**Affiliations:** 1 State Key Laboratory of Respiratory Health and Multimorbidity; NHC Key Laboratory of Comparative Medicine; National Human Diseases Animal Model Resource Center, National Center of Technology Innovation for animal model; Beijing Key Laboratory for Animal Models of Emerging and Remerging Infectious Diseases; Key Laboratory of Pathogen Infection Prevention and Control, Ministry of Education, National Institute of Pathogen Biology; Institute of Laboratory Animal Science, Chinese Academy of Medical Sciences and Comparative Medicine Center, Peking Union Medical College, Beijing, China; 2 Institute of Aging and Health, Chinese Academy of Medical Sciences and Peking Union Medical College, Beijing, China; 3 Plastic Surgery Hospital, Chinese Academy of Medical Sciences and Peking Union Medical College, Beijing, China

**Keywords:** Endocrine system and metabolic diseases, Infectious diseases, Infection

## Abstract

The global diabetes incidence has surged post-COVID-19, yet how SARS-CoV-2 drives long-term beta cell (β-cell) failure remains incompletely elucidated. We identified a mechanism whereby SARS-CoV-2 structural proteins directly hijack islet amyloid polypeptide (IAPP) to induce pathogenic amyloid deposition, disrupting insulin granule maturation, trafficking, and secretion in an age-susceptible islet microenvironment. In macaque models, aged subjects showed extensive islet IAPP aggregation with increased β-cell necroptosis, whereas adult infected animals exhibited minimal IAPP pathology. Multiplex fluorescence in situ hybridization detecting viral sense and antisense RNAs with IAPP immunostaining revealed focal overlap of active viral replication foci with IAPP-rich amyloid regions in aged islets. Molecular docking was used as a hypothesis-generating tool, whereas coimmunoprecipitation, proximity ligation assays, and surface plasmon resonance experimentally confirmed direct spike/nucleocapsid-IAPP binding; interface-guided peptide mutagenesis further showed that five predicted IAPP contact residues are required for binding. Thioflavin-T kinetics and electron microscopy demonstrated that the spike/nucleocapsid markedly accelerated IAPP monomer conversion into dense fibrillar networks. In β cells and primary hIAPP-overexpressing mouse islets, spike/nucleocapsid expression or protein exposure reduced insulin content and induced endoplasmic reticulum stress and inflammatory programs. Integrated spatial islet proteomics linked IAPP-viral complexes to complement activation and vesicle transport dysfunction. Crucially, primary islet qPCR and immunoblotting revealed stalled insulin mRNA with compensatory upregulation of trafficking/exocytosis transcripts but accumulation of insulin precursor proteins, consistent with impaired granule maturation/processing. Thus, SARS-CoV-2 proteins act as pathogenic “seeds” for IAPP assembly, establishing an IAPP aggregation-impaired granule maturation-complement activation-vesicle transport blockade axis underlying age-associated vulnerability to post-COVID-19 diabetes.

## Introduction

Clinical evidence has demonstrated that SARS-CoV-2 infection not only induces acute respiratory syndromes but is also intricately linked to the onset and exacerbation of various chronic metabolic diseases, such as diabetes.^1,2^ Epidemiologic and clinical studies have reported increased diabetes diagnoses and worsened glycemic control following SARS-CoV-2 infection, particularly among patients with severe disease or preexisting metabolic risk.^2,3^ These findings, corroborated by large-scale cohort studies, underscore a positive correlation between SARS-CoV-2 infection and long-term diabetes risk, highlighting the urgent need for healthcare systems to address the emerging burden of chronic metabolic diseases in the postpandemic era.^1,4^ However, the mechanistic basis by which a respiratory virus contributes to durable β-cell dysfunction remains incompletely defined, and competing models include direct endocrine infection, indirect inflammatory injury, altered insulin sensitivity, and stress hormone-driven dysregulation.

Experimental work has demonstrated that SARS-CoV-2 can access the pancreas^5–7^ and that pancreatic exocrine tissue is frequently involved,^8,9^ while endocrine involvement may be focal and context-dependent. Multiple studies, including our prior work, support age as a major modifier of pancreatic vulnerability in vivo, with older non-human primates (NHPs) exhibiting more severe islet pathology than adults under comparable viral exposure conditions.^10–13^ In particular, our previous study documented SARS-CoV-2 distribution in both exocrine and endocrine compartments and described age-linked endocrine injury, fibrosis, and impaired insulin-related readouts in older animals.^14^ However, the proximal molecular trigger that converts viral exposure into progressive islet failure has remained unclear.

A central, disease-relevant vulnerability of β cells is proteostasis stress around insulin granule biogenesis and peptide hormone co-packaging. Islet amyloid polypeptide (IAPP; amylin) is co-secreted with insulin and can misfold into oligomers and fibrils; toxic IAPP oligomers are implicated in β-cell dysfunction and loss during diabetes progression. While host factors influencing IAPP misfolding are known, it is not established whether viral structural proteins can directly bind a host endocrine peptide and catalyze amyloid assembly in vivo. The hypothesis that a respiratory virus could physically interact with, hijack, and maliciously accelerate the aggregation of a host endocrine hormone introduces a highly complex, multisystemic pathogenic paradigm that bridges virology and amyloidosis. The proposition that viral structural proteins can act as cross-seeding nucleators for host amyloidogenic proteins is currently revolutionizing neurodegenerative research. Recent groundbreaking studies have demonstrated that SARS-CoV-2 can accelerate amyloid-β (Aβ) pathology in ex vivo human retinal explants and cerebral organoids, driving pathological signatures analogous to Alzheimer’s disease.^15^ Despite these advancements in the central nervous system (CNS), parsing the relative contributions of direct viral-mediated amyloidosis versus bystander injury in the endocrine system has proved exceptionally challenging.

In this study, we present a unified, multi-layered mechanistic framework elucidating how SARS-CoV-2 induces terminal endocrine failure. Using aged and adult rhesus macaque tissues, in silico interface modeling, quantitative binding assays, and β-cell/primary humanized IAPP (hIAPP) transgenic mouse islet model systems, we define a virus–host interaction axis that links spike/nucleocapsid-IAPP binding to amyloid deposition, complement/inflammatory activation, and impaired insulin granule maturation and trafficking. We introduce robust 5-amino-acid mutant surface plasmon resonance (SPR) assays and thioflavin-T (ThT) aggregation kinetics to prove the direct nucleating capability of viral spike and nucleocapsid proteins. Utilizing high-resolution spatial multiomics, triple-labeling in situ hybridization (identifying active antisense viral replication), and absolute ultrastructural quantification, we define a “physical entanglement” model wherein viral-seeded IAPP webs mechanically trap proinsulin cargo and sequester vital membrane-trafficking machinery. Together, these findings fundamentally redefine post-COVID-19 metabolic disease as a virally accelerated, mechanically driven amyloidosis, offering precise molecular targets for clinical intervention.

## Results

### Aged infection is associated with extensive IAPP amyloid deposition and insulin loss in primate islets

In our previous studies, SARS-CoV-2 infection in adult rhesus macaques caused minimal pancreatic damage, characterized by limited inflammatory responses and subtle histological changes.^14^ Here, we extend our investigation to explore the cellular basis linking SARS-CoV-2 infection and islet amyloid plaque formation in aged rhesus macaques. Hematoxylin-eosin (H&E) staining of pancreatic sections from aged infected monkeys revealed pronounced eosinophilic amyloid deposits in islets, whereas aged uninfected controls showed only minimal or focal amyloid, and adult control monkeys and adult infected monkeys had none. We confirmed these deposits to be islet amyloid by immunostaining for IAPP and by Thioflavin S staining, which binds amyloid fibrils. Biochemical analysis of the adult infected cohort revealed significantly elevated fasting C-peptide levels and C-peptide/glucose ratios,^10,14^ indicating that the adult host mounts an early, resilient secretory compensation to counteract systemic insulin resistance, crucially without the manifestation of structural amyloidosis.

**Fig. 1:**
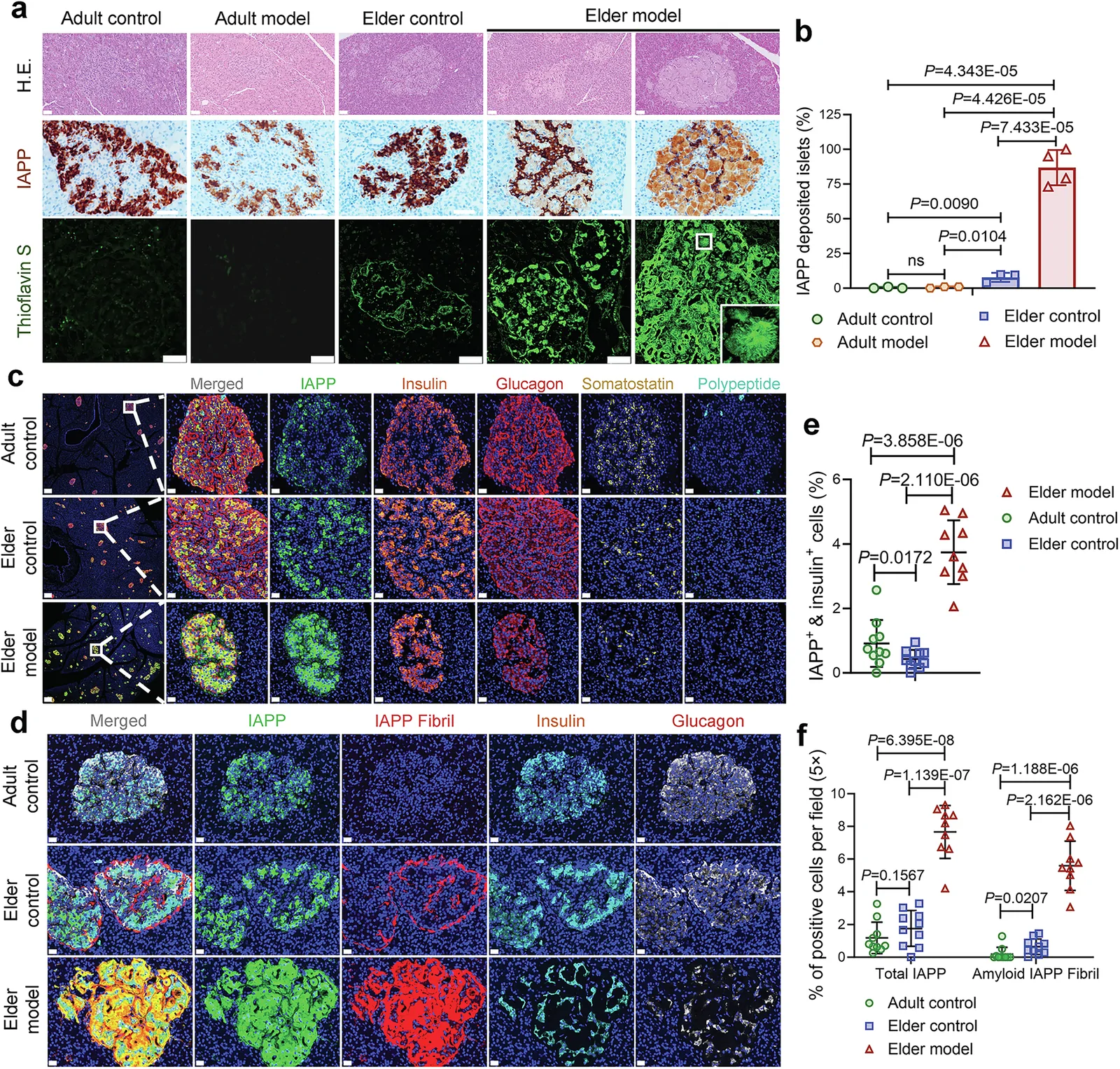
Age- and infection-associated IAPP aggregation in primate pancreatic islets. **a** Representative pancreatic sections from adult control (AC) (*n* = 3), SARS-CoV-2-infected adult model (AM) (*n* = 3), elder control (EC) (*n* = 3), and SARS-CoV-2-infected elder model (EM) (*n* = 4) macaques stained with H&E, IAPP (brown, DAB), and thioflavin S (green) to detect amyloid fibrils. Boxes indicate higher-magnification regions. Scale bars, 50 µm. **b** Quantification of the IAPP-positive area as a percentage of the total islet area (*n* = 3–4 slides/animal/group). **c** Multiplex immunofluorescence of IAPP (green) and major islet hormones (insulin-orange, glucagon-red, somatostatin-yellow, pancreatic polypeptide-cyan) reveals disrupted islet architecture and prominent IAPP accumulation in EM animals. Low-magnification image with scale bar, 200 μm. Insets show enlarged views, Scale bars, 20 µm. **d** Multiplex immunofluorescence of IAPP (green), fibril-specific IAPP conformational antibody (red), insulin (cyan), and glucagon (white) reveals extensive amyloid fibrils within β-cells of EM islets. Scale bars, 20 µm. **e** Quantification of IAPP^+^/insulin^+^ double-positive β-cells per islet field (×20 magnification; *n* = 10 images examined in total/group). **f** Total IAPP^+^ area (left) and amyloid IAPP fibril^+^ area (right) as percentages of islet area (×20 magnification; *n* = 10 images examined in total/group). Statistical analysis: ten images from 3–4 animals per group were quantified. Measurements from the same animal were averaged, and animal-level means were used for statistical testing. Data are presented as the mean ± SD. *p* values were calculated by unpaired two-tailed Student’s *t* test. *p < 0.05, **p < 0.01, and ***p < 0.001

Specifically, our findings indicate the following:

a. Under physiological conditions, aging is associated with modest, punctate or focal amyloid deposits in islets (22/167 = 9.58%, *P* = 0.0090 vs. adult group), whereas no such deposits were detected in adults (0/185). b. SARS-CoV-2 infection markedly promoted IAPP deposition in aged islets, with a significant increase in both the number of positive islets (441/496 = 88.91%, *P* = 4.343E-05 vs. adult group, *P* = 7.433E-05 vs. elder group) and plaque area. c. Two distinct pathological phenotypes of IAPP accumulation were observed in the infected aged group: one featured swollen, degenerative β-cells retaining cord-like β-cell structures with faint pericellular IAPP expression; the other showed profound β-cell loss with dense, dark brown IAPP-positive amyloid plaques filling the interstitial space (Fig. 1a, b). Consistent with fibrillar amyloid, thioflavin S staining was strongly positive in aged infected islets.

Building on our prior findings that SARS-CoV-2 infection reduces α-, β-, and δ-cell populations in aged monkeys, we next employed multiplex panoramic immunofluorescence to investigate IAPP localization under physiological and pathological conditions. We assessed IAPP’s spatial relationship with insulin^+^ β-cells, glucagon^+^ α-cells, somatostatin^+^ δ-cells, and pancreatic polypeptide^+^ PP-cells. Under normal conditions, while total IAPP protein levels were similar between adults and aged monkeys, the proportion of insulin^+^ IAPP^+^ double-positive β-cells declined from 1.02% to 0.42% with age (Fig. 1c–f). Upon SARS-CoV-2 infection, this ratio rose significantly to 3.61%, with widespread accumulation of IAPP around β-cells. These IAPP-positive regions exhibited a loss of cellular contours and adopted amorphous plaque-like morphology (Fig. 1c–e).

Amyloid formation involves aberrant protein aggregation. IAPP, in this context, transitions from its native soluble state to insoluble amyloid fibrils. To determine whether insoluble IAPP fibers were present, we further quantified their expression. In physiological states, fibrous IAPP was negligible in adults (0.19%) and modestly increased in aged controls (0.64%), mainly peri-islets and not colocalized with β-cells. However, SARS-CoV-2 infection significantly elevated IAPP fibril formation (5.59%), which was broadly distributed across islets, displacing both α- and β-cells. Consistent with this, insulin and glucagon expression were both markedly reduced in infected islets (Fig. 1d, f). The minimal IAPP plaque formation in the adult infected animals compared with aged infected animals supports age as a major modifier of this phenotype.

### SARS-CoV-2 RNA and protein signals co-localize with IAPP aggregates in pancreatic β-cells, inducing ultrastructural pathology and β-cell necroptosis

**Fig. 2:**
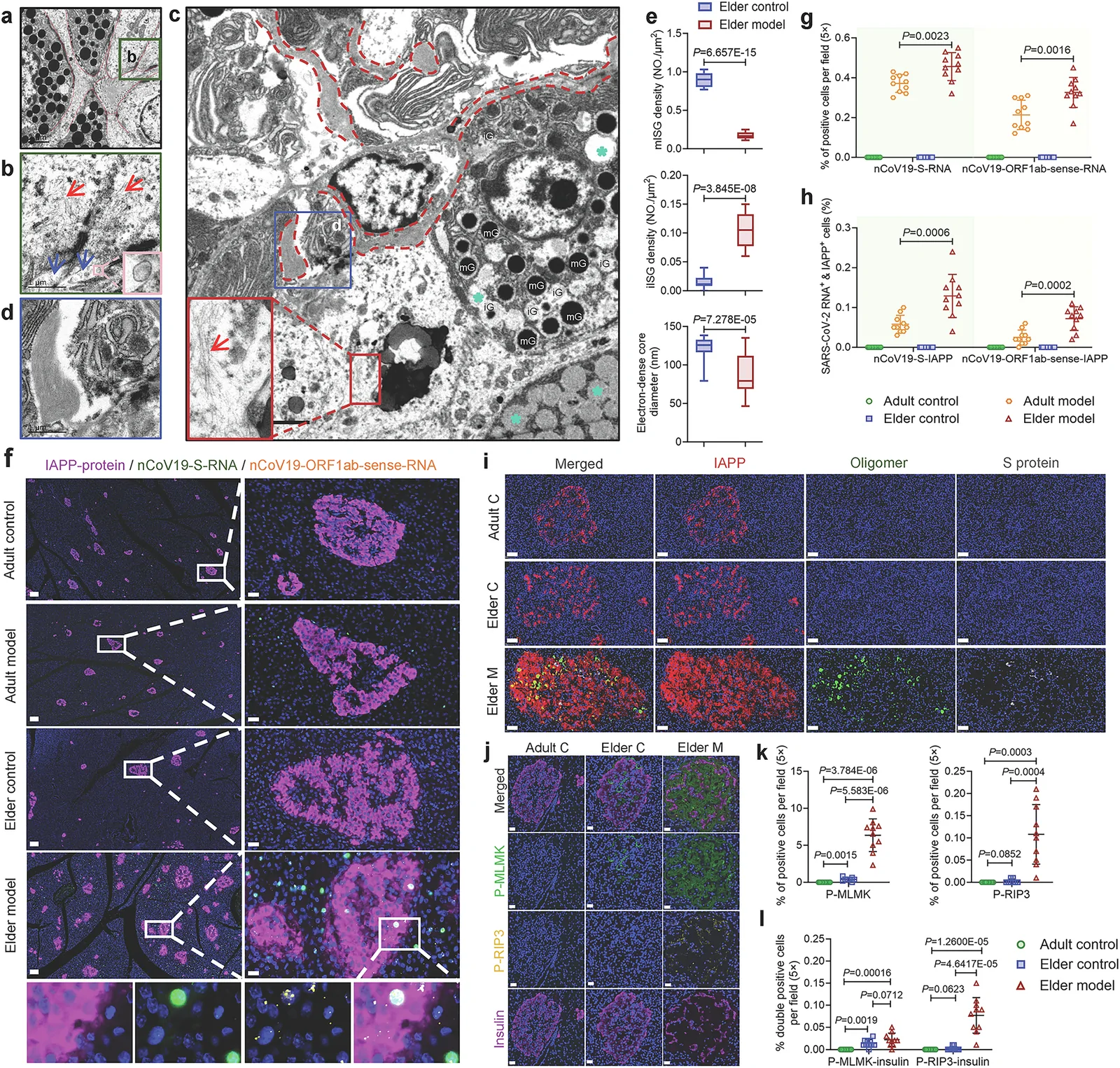
Ultrastructural pathology, focal plaque-associated viral RNA signals, and necroptosis activation in infected aged islets. **a**–**d** Ultrastructural abnormalities in β-cells from SARS-CoV-2-infected aged monkey islets. TEM of EM β-cells showing extensive fibrillar aggregates (red dashed lines), vesicular accumulation (blue arrows), and disrupted endomembranes. TEM of β-cells from EC animals showing markedly depleted insulin granules (cyan asterisk). Progressive magnification of infected EM β-cells showing large amyloid-like deposits, fragmented organelles, and dense fibrils (red arrows). Scale bars: **a** 2 µm; **b** 1 µm; **c** 5 µm; **d** 1 µm. **e** TEM-based quantification of mature insulin granules (mG), immature insulin granules (iG), and granule diameter distribution (*n* = 10 images/group). **f** Multiplex fluorescence in situ hybridization (FISH) for SARS-CoV-2 positive-sense genomic RNA (green) and negative-sense (antisense) RNA (yellow) and immunofluorescence for IAPP (magenta) in AC, AM, EC, and EM islets. Representative fields show focal overlap of a subset of intra-islet viral RNA signals, particularly antisense RNA-positive replication foci, with IAPP-rich plaque regions in EM islets. DAPI, blue. Low-magnification image, scale bar 100 μm; enlarged views, scale bars 20 μm. **g** Quantification of intra-islet positive-sense RNA-positive and antisense RNA-positive foci per field (×20 magnification; *n* = 10 images examined in total/group). **h** Object-based quantification of the percentage of antisense RNA-positive replication foci overlapping IAPP-positive plaque regions per field (×20 magnification; *n* = 10 images examined in total/group). **i** Co-detection of IAPP (red), oligomeric IAPP (green), and SARS-CoV-2 spike protein (gray) showing colocalization in EM islets. Scale bars, 50 µm. **j** Immunofluorescence for the necroptotic markers P-MLKL (green) and P-RIP3 (yellow) and insulin (magenta) reveals necroptotic activation in EM β-cells. Scale bars, 20 µm. **k** Quantification of P-MLKL^+^ and P-RIP3^+^ cells per islet field (×20 magnification; *n* = 10 images examined in total/group). **l** Quantification of IAPP + /P-MLKL^+^ and IAPP + /P-RIP3^+^ double-positive cells per field (×20 magnification; *n* = 10 images examined in total/group). Statistical analysis: 10 images from 3 to 4 animals per group were quantified. Measurements from the same animal were averaged, and animal-level means were used for statistical testing. Data are presented as the mean ± SD. *p* values were calculated by unpaired two-tailed Student’s *t* test: **p < 0.01; ***p < 0.001; ns not significant

Transmission electron microscopy (TEM) revealed profound β-cell ultrastructural abnormalities in aged infected islets, including cytoplasmic high-electron-density fibrillar material (red arrow) consistent with amyloid, widened intercellular gaps (Fig. 2a), large numbers of unbound vesicular structures scattered both within β-cells and in the enlarged gaps between cells (Fig. 2b, blue arrow), and depletion of electron-dense insulin granules (Fig. 2a–d) compared with controls (Supplementary Fig. 1a, b). The infected β-cells had a striking depletion of insulin granules in the cytoplasm (cyan asterisk), indicating loss of insulin storage (Fig. 2c). To elevate these qualitative observations to a standard of absolute rigorous scientific quantification, we conducted a detailed morphometric analysis of vesicle subpopulations per 100 µm² of β-cell cytoplasm (Fig. 2e). In the aged infected cohort, the density of functional, mature insulin granules (mG) (distinctly identified by their electron-dense crystalline cores and clear halos) plummeted by over 80% compared to adult controls. Concurrently, the population of immature granules (iG) or empty “ghost vesicles” (cyan asterisk) skyrocketed nearly threefold. In some regions, fibrils aggregated into higher-order bundles or parallel arrays (Fig. 2c, d). These fibril bundles were found between dilated endoplasmic reticulum (ER) cisternae, often compressing and distorting the ER (Fig. 2c, red dashed line). These ultrastructural findings confirm that SARS-CoV-2 infection in aged islets leads to massive intracellular and extracellular IAPP fibril accumulation, with concomitant damage to cellular organelles (ER and membranes) and loss of insulin secretory granules (Fig. 2c, d). The TEM evidence dovetails with the histological data, indicating that β-cells in infected islets are both under stress (ER dilation, lysosomal overload) and lose their ability to store insulin (few granules left), likely resulting in impaired insulin secretion.

To determine whether active intra-islet viral replication could be detected within IAPP-rich lesions, we performed a “2 RNA + 1 protein” multiplex FISH/immunofluorescence assay for SARS-CoV-2 positive-sense RNA, negative-sense RNA, and IAPP (Fig. 2f). We emphasize that this assay was designed to detect focal spatial association rather than to imply that all pancreatic or all intra-islet viral signals are restricted to IAPP-positive cells (Supplementary Fig. 1c). As expected, positive-sense RNA showed a broader distribution, whereas negative-sense RNA marked more discrete replication-associated foci. Both sense and antisense viral RNA signals were increased in aged infected islets compared with adult infected islets (Fig. 2g). Object-based spatial analysis further showed that 0.072% of antisense RNA-positive replication foci overlapped IAPP-positive plaque regions in aged infected islets (Fig. 2h), supporting focal enrichment of active viral replication within amyloid-rich lesions.

We probed for IAPP oligomers in the infected islets because oligomeric intermediates are the most toxic species in amyloid diseases.^16^ Using an oligomer-specific antibody, we found that IAPP oligomers were detectable only in infected aged islets, not in other control islets. The oligomer signals appeared as scattered puncta intermingled with broader IAPP protein deposits (Fig. 2i). Some of the IAPP oligomer puncta co-localized with viral spike (S) protein signals in the islet, which we visualized by immunostaining for the SARS-CoV-2 S protein (Fig. 2i). These observations indicate that SARS-CoV-2 infection leads to the formation of toxic IAPP oligomers within islets, and viral proteins are present in close proximity to these IAPP aggregates.

Given that IAPP oligomers are known to be cytotoxic, we examined whether β-cells in infected islets were undergoing cell death. Our previous work noted necrotic cell death in infected islets.^14^ To provide definitive quantitative evidence, we performed triple-labeling immunofluorescence for the terminal executioner proteins of necroptosis: phosphorylated MLKL (p-MLKL) and phosphorylated RIP3 (p-RIP3),^17^ alongside insulin (Fig. 2j). While both adult and elderly uninfected controls exhibited near-zero baseline expression of these markers, the aged infected islets displayed a massive upregulation (Fig. 2k). p-MLKL was intensely localized to the ruptured intercellular boundaries of actively dying β-cell clusters, while numerous residual insulin-positive cells exhibited intense p-RIP3 costaining (Fig. 2l). This quantitative analysis definitively proves that the physical and biomechanical insult of viral-seeded IAPP oligomer/amyloidosis specifically triggers the highly inflammatory RIP3/MLKL-mediated necroptotic pathway.

### Spike and nucleocapsid directly bind proIAPP validated by orthogonal assays

**Fig. 3:**
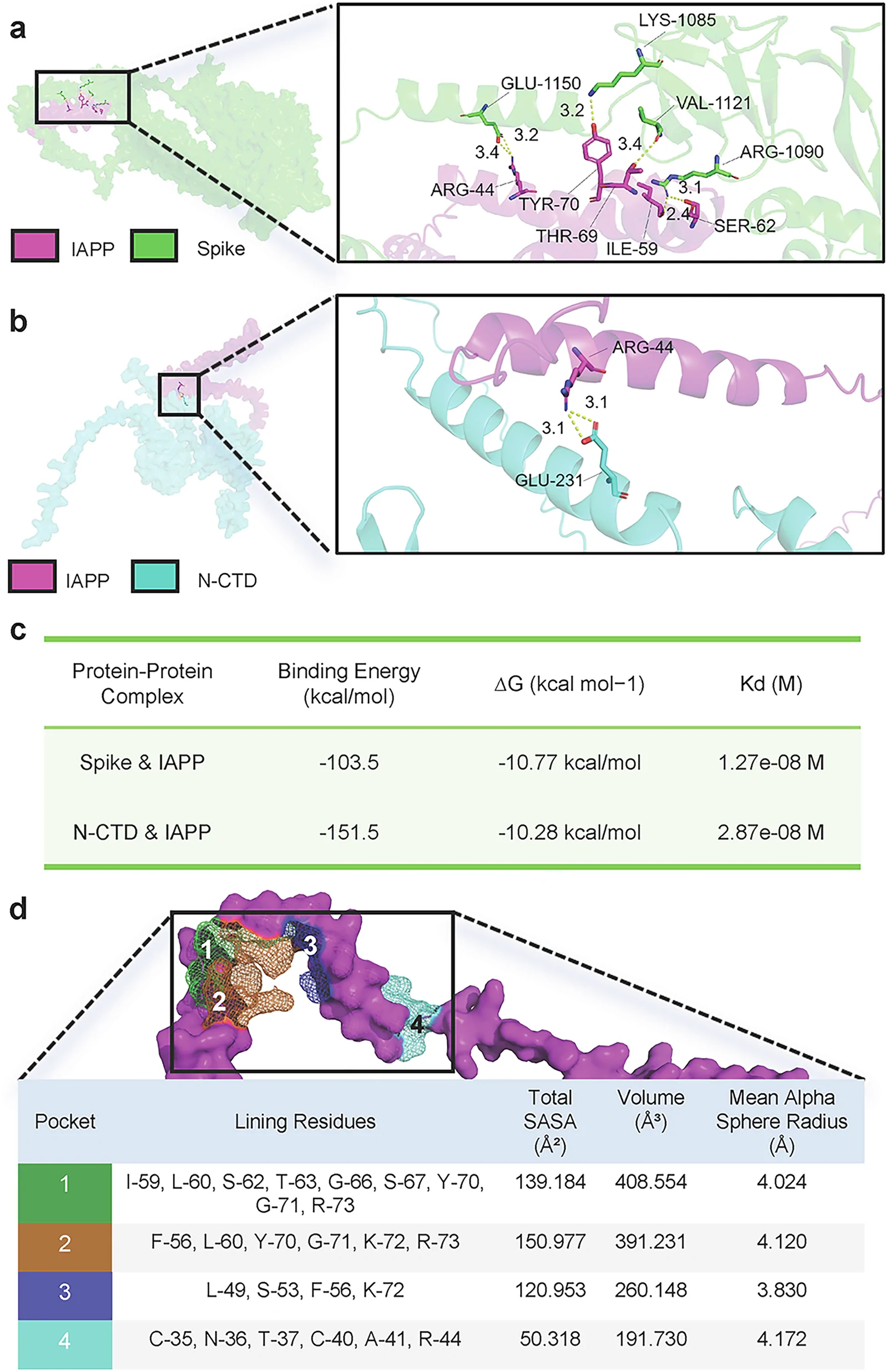
Structural modeling suggests putative proIAPP contact regions for spike and nucleocapsid binding. **a**, **b** Docking-derived candidate interface models showing putative contact residues between IAPP and Spike (**a**) or N-CTD (**b**). **c** Model-derived interaction energy estimates for the candidate complexes. These values are shown as qualitative computational support and were not used as experimental affinity measurements. **d** To evaluate druggability at the virus-IAPP interface, we performed geometry-based pocket mapping on the IAPP structure

SARS-CoV-2 Spike (S) and Nucleocapsid (N) are major structural proteins of the virus.^18^ We used AlphaFold3 and complementary docking as a hypothesis-generating approach to nominate candidate contact surfaces between proIAPP and the Spike or N-CTD protein (Supplementary Table 1). Because the AF3 interface confidence values were modest, these models were not used as standalone structural evidence. Instead, they served to guide residue selection for downstream experimental testing. The modeling suggested two putative IAPP regions (residues 35–44 and 59–73) that might contribute to viral protein engagement (Fig. 3a, b). At the Spike–IAPP interface, IAPP Arg44, Tyr70, Thr69, Ile59 and Ser62 formed polar contacts/salt bridges with Spike Glu1150, Lys1085, Val1121 and Arg1090 at 2.4–3.4 Å distances (PyMOL measurements on AF3 models), whereas in the N-CTD-IAPP complex, IAPP Arg44 engaged N-CTD Glu231 (~3.1 Å) (Fig. 3a, b). The binding energetics calculated via GMX-MM-PBSA indicated spontaneous and stable complex formation, generating affinities of −103.5 kcal/mol for S-IAPP (*K*_d_ = 1.27 × 10^−8^ M) and −151.5 kcal/mol for N-IAPP (*K*_d_ = 2.87 × 10^−8^ M) (Fig. 3c).

To evaluate druggability at the virus-IAPP interface, we performed geometry-based pocket mapping on the IAPP structure. Four candidate pockets were identified on the IAPP surface (Fig. 3d). Pocket 1 (lining residues I59-L60-S62-T63-G66-S67-Y70-G71-R73) and Pocket 2 (F56-L60-Y70-G71-K72-R73) were the largest and most solvent-exposed (SASA 139.2/151.0 Å²; volumes 408.6/391.2 Å³), overlapping the 59–73 contact cluster engaged by Spike/N-CTD. Pocket 4 (C35-N36-T37-C40-A41-R44) encompassed Arg44, the recurrent hotspot for both viral proteins, while Pocket 3 (L49-S53-F56-K72) bridged the two clusters (Fig. 3d). Pocket mapping further identified solvent-accessible features around these regions that may be useful for future inhibitor design.

Functionally, the AF3-defined hotspots preproIAPP Arg44 (maps to IAPP Arg11 in the N-terminal membrane-binding helix)^19^ and preproIAPP 59–70 (maps to IAPP 26-37 overlapping the canonical amyloid core and β-prone C-terminal stretch)^20^ colocalize with regions implicated in membrane-catalyzed nucleation and fibril growth, respectively. Thus, the structural complementarity and drug-addressable IAPP pockets together offer a mechanistic explanation for how viral S/N proteins bias IAPP toward aggregation, in line with the in situ observation of IAPP oligomers/fibrils and β-cell injury in aged infected primates described above.

**Fig. 4:**
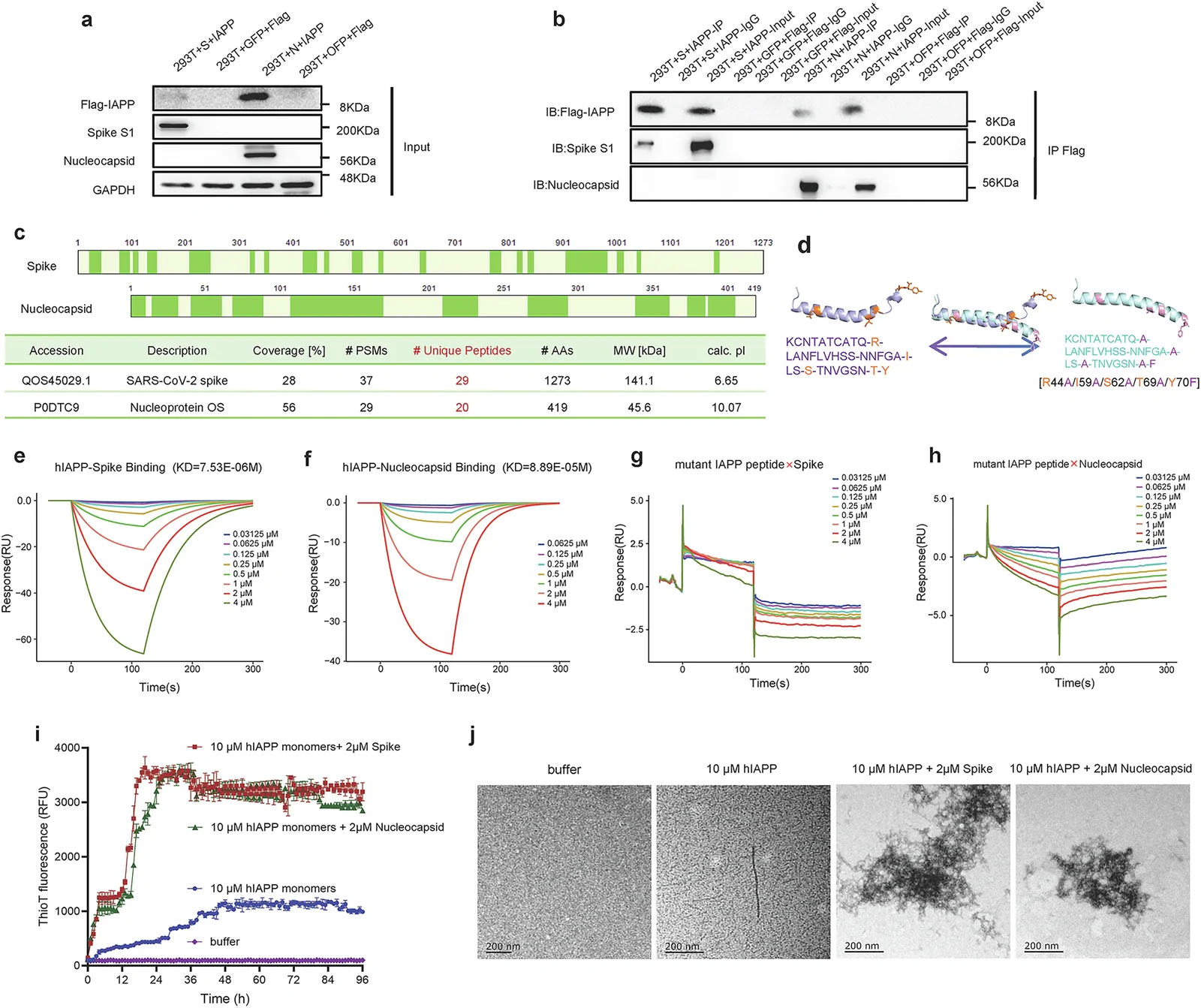
Orthogonal validation of spike/nucleocapsid-IAPP interactions and interface dependence. **a** Western blot confirming successful co-expression of target proteins in HEK293T cells. **b** IP assays show specific interactions between Flag-IAPP and either Spike or Nucleoprotein. IgG control and empty vector controls validate specificity. **c** MS analysis identifies unique peptides for both Spike (29) and Nucleoprotein (20), confirming direct interactions. **d** Comparison of the IAPP proprotein fragment (residues 37–70) between wild-type and the pentamutant (R44A/I59A/S62A/T69A/Y70F). **e**, **f** SPR sensorgrams for binding of wild-type IAPP to the immobilized spike protein (**e**) or immobilized nucleocapsid protein (**f**). Viral proteins were immobilized on the sensor chip, and IAPP was injected as the analyte. Concentration-dependent responses yielded measurable binding in the micromolar range. **g**, **h** SPR sensorgrams showing loss of detectable binding when the IAPP pentamutant was injected over immobilized Spike (**g**) or immobilized Nucleocapsid (**h**). **i**, **j** Thioflavin-based aggregation kinetics and EM visualization showing accelerated IAPP amyloid assembly and larger aggregates upon addition of Spike or Nucleocapsid

To experimentally validate these interactions, we co-transfected PANC-1, a human pancreatic cell line, with plasmids expressing SARS-CoV-2 S protein (C-terminus-GFP tag), N protein (C-terminus-OFP tag), and IAPP (C-terminus-Flag tag). The expression of IAPP in PANC-1 cells was confirmed (Supplementary Fig. 1d), and confocal immunofluorescence demonstrated perinuclear co-localization of IAPP with both S and N proteins (Supplementary Fig. 1e). To more sensitively detect direct interactions, we turned to coimmunoprecipitation (Co-IP) assays in HEK293T cells (which have high transfection efficiency). Four western blot lanes were prepared: lane 1, S-GFP + IAPP-Flag co-transfection; lane 2, GFP + Flag empty vectors (control); lane 3, N-OFP + IAPP-Flag co-transfection; and lane 4, OFP + Flag empty vectors (control) (Figs. 4a and Supplementary Fig. 2a, original data). As expected, none of the three proteins was expressed endogenously in HEK293T cells. Successful expression of S + IAPP (lane 1) and N + IAPP (lane 3) was confirmed, while the control groups showed no protein expression. We then performed co-IP assays using specific antibodies and IgG isotype controls to eliminate nonspecific interactions. Coomassie blue staining (Supplementary Fig. 2b) verified the integrity of immunoprecipitated proteins, and negative controls confirmed the specificity of the interaction. Strikingly, IAPP showed specific interactions with both the S and N proteins (Figs. 4b and Supplementary Fig. 2c, original data).

To further validate these findings, we subjected immunoprecipitated complexes to mass spectrometry (MS) analysis. Protein identification in the IAPP-containing complexes confirmed the presence of 29 unique peptides from the SARS-CoV-2 S protein (Accession: QOS45029.1) and 20 unique peptides from the N protein (Accession: P0DTC9), confirming the direct physical interaction of IAPP with both viral structural proteins (Fig. 4c).

### Spike and Nucleocapsid bind proIAPP through a discrete interface and accelerate IAPP amyloid kinetics

To definitively validate these in silico docking models in vitro, we rationally designed a mutant IAPP peptide. Based on the docking interface, the five critical interacting amino acids were substituted with alanine or phenylalanine [R44A/I59A/S62A/T69A/Y70F], effectively destroying the predicted binding pocket (Fig. 4d). SPR was employed to precisely measure binding kinetics. Wild-type IAPP demonstrated robust, high-affinity binding to both viral proteins in the low micromolar range (equilibrium dissociation constant KD for S-IAPP: 7.53E-06 M; KD for N-IAPP: 8.89E-05 M) in a concentration-dependent manner (Fig. 4e, f). Strikingly, when the 5-amino-acid mutant peptide was flowed over the SPR sensor chip, binding was entirely abolished. The response signals flatlined at baseline noise levels, demonstrating zero interaction with either the S (Fig. 4g) or N proteins (Fig. 4h). This binary loss-of-function confirms that these 5 specific amino acid residues constitute the interface for viral “hijacking” of IAPP. As an orthogonal exploratory assay using a published IAPP-binding chaperone-related probe, recombinant ITM2B bound free IAPP with high affinity, whereas its interaction with the preformed Spike–IAPP complex was markedly weakened, and no measurable binding was detected for the preformed Nucleocapsid-IAPP complex (Supplementary Fig. 2d–f). These data suggest that viral proteins may partially or completely mask/remodel an ITM2B-accessible IAPP surface.

To dynamically track the functional consequence of this binding, we conducted in vitro ThT aggregation kinetics assays. Wild-type IAPP monomers alone exhibited a prolonged lag phase and a slow, linear fibrillization curve. However, the introduction of either isolated S or N proteins into the monomeric IAPP solution induced a radical thermodynamic shift: the lag phase was virtually eliminated, and the fluorescence intensity spiked exponentially within minutes, indicating rapid, massive aggregation (Fig. 4i). The corresponding 24-hour negative-stain and TEM of these in vitro incubations visually corroborated the biophysics. While IAPP alone formed only sparse, isolated protofibrils after 24 hours, the IAPP + S/N co-incubations generated massive, hyperdense, and highly entangled macromolecular fibril webs (Fig. 4j), phenocopying the catastrophic amyloid plaques observed in the aged NHP islets. By physically “hijacking” IAPP, the virus may seed or stabilize IAPP aggregates, linking viral infection to islet amyloid formation.

### IAPP-virus interactions impair β-cell insulin storage and blunt stimulus-evoked secretion in MIN6 β-cells while driving disproportionate EV release

**Fig. 5:**
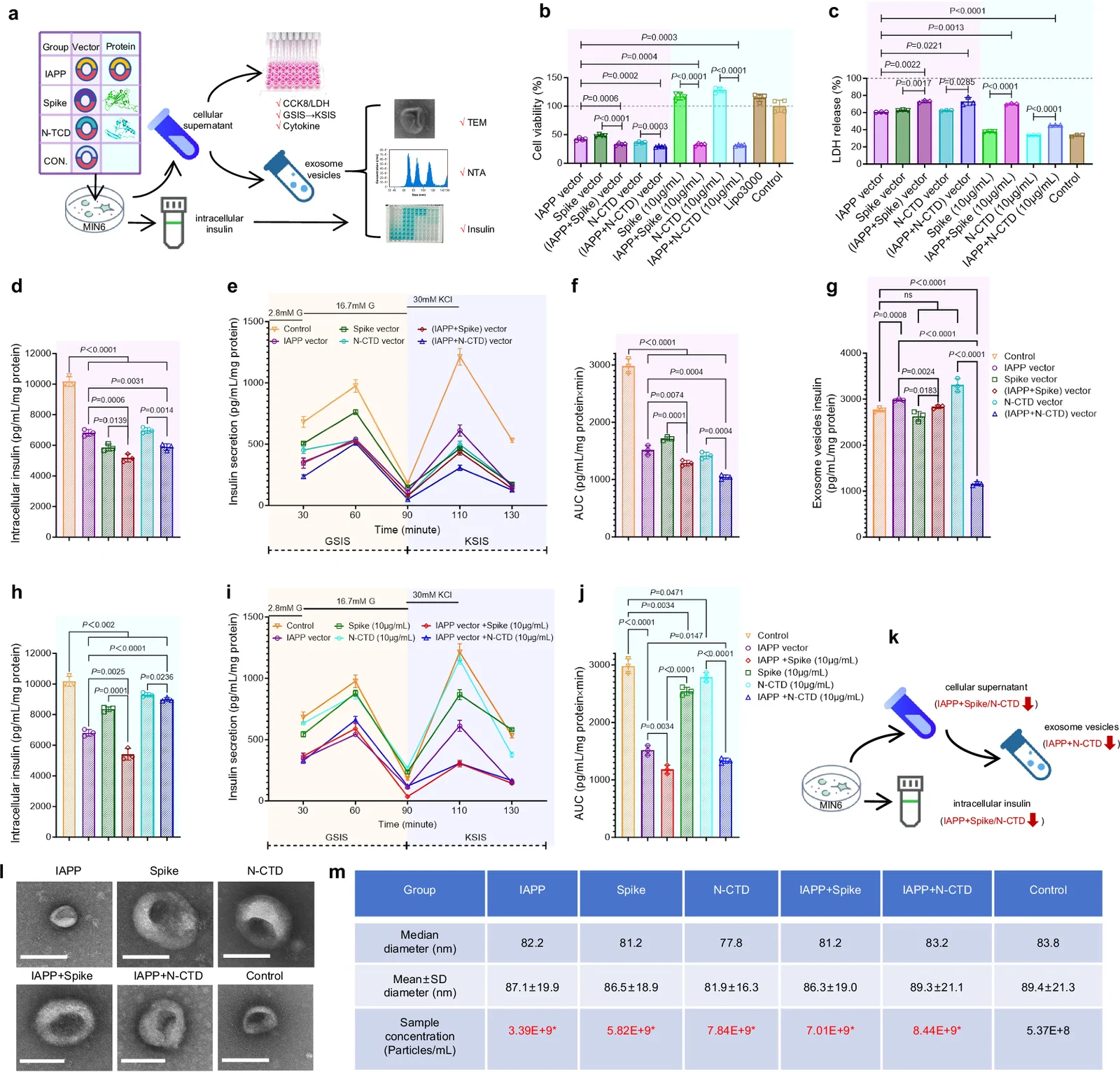
In vitro evidence that SARS-CoV-2 S/N proteins interact with IAPP impairs β-cell viability and insulin secretion (MIN6 cell experiments). **a** Schematic overview of the MIN6 experimental design, including vector-based expression and short-term protein exposure conditions. **b**, **c** Cell viability assays (CCK-8 and LDH) in MIN6 cells: Bar graphs show per cent viability relative to control, (*n* = 4 biological replicates) (**b**). LDH release (cytotoxicity) was minimal in the control, slightly increased with single transfections, and significantly higher with IAPP + S or IAPP + N (indicating more cell membrane damage) (*n* = 3 biological replicates) (**c**). Values are reported as the mean±SEM and represent normalized units. Multiple comparisons were evaluated by two-sided Welch’s *t* tests with Holm correction within each family of tests (vs control). **d**–**g** Detection of intracellular insulin content, GSIS, KSIS, and exosome vesicle insulin in MIN6 cells transfected with plasmids expressing IAPP, spike, and N (either individually or in combination): bar graph of cellular insulin content (normalized to protein) in different groups. IAPP + S and IAPP + N cells had lower insulin content than both the alone and control cells (*p* < 0.01) (**d**). Glucose-stimulated insulin secretion (GSIS) results: insulin secretion (% of content released during 90 min 16.7 mM glucose stimulation after basal) revealed that combined IAPP+viral protein causes a severe secretory defect. Values are reported as the mean±SEM and represent normalized units (a.u.). KCl-stimulated insulin secretion (KSIS): High K+ (30 mM)-induced insulin release is also significantly reduced in IAPP + S and IAPP + N cells. This indicates a fundamental exocytotic machinery impairment rather than just glucose sensing (**e**). AUC analysis of insulin secretion induced by GSIS (30–90 min) and KSIS (90–130 min) is shown in (**f**). Insulin content within EVs (ELISA) was detected. IAPP + S and IAPP + N EVs carry significantly less. The total EV-insulin output was even lower than that of the control despite more vesicles (particularly low in the IAPP + N group) (**g**). Values are reported as the mean ± SEM and represent normalized units. Multiple comparisons were evaluated by two-sided Welch’s *t* tests with Holm correction within each family of tests (vs control). **h**–**j** Detection of intracellular insulin content, GSIS, and KSIS in MIN6 cells incubated with IAPP, spike, and N proteins (either individually or in combination). AUC analysis of insulin secretion induced by GSIS (30–90 min) and KSIS (90–130 min) shown in j. Protein experiments recapitulated these trends with lower basal toxicity. **k** Schematic diagram of the results of analyzing the insulin secretion status using the MIN6 cell model. (**l**,**m**) Extracellular vesicle (EV) secretion and insulin cargo: representative TEM images of exosomes from control vs IAPP + N co-expression culture (**l**). Both show typical 60–120 nm vesicles, while in IAPP + N, vesicles appear more numerous (consistent with NTA data) yet have reduced insulin. Scale, 100 nm. NTA quantification of EV particles per mL in each group is shown in m

We next investigated the functional consequences of the IAPP–S/N interaction on β-cell health and insulin secretion. We utilized the MIN6 pancreatic β-cell line (a mouse insulinoma cell line that produces insulin and IAPP) as an in vitro model. We designed experiments to simulate the co-presence of viral proteins and IAPP within β-cells using two perturbation modes: (i) plasmid overexpression (“vector”) and (ii) short-term protein exposure (“protein”) (workflow in Fig. 5a).

We first assessed whether the expression of IAPP, S, and N proteins affects β-cell viability. MIN6 cells were transfected with plasmids expressing IAPP, Spike, and N (either individually or in combination), and cell viability was measured by the CCK-8 assay (which measures metabolic activity) (Fig. 5b) and LDH release assay (which measures cytotoxicity) (Fig. 5c). Vector conditions reduced cell viability and increased cytotoxicity, with the IAPP+Spike and IAPP + N-CTD combinations most affected (CCK-8, 33.1 ± 1.0% and 29.2 ± 0.5% of control 100 ± 5.3%; LDH, 73.0 ± 0.9% and 73.0 ± 2.7% vs 33.3 ± 0.6%; all adjusted *p* < 0.01 vs control). Single-vector constructs showed intermediate toxicity (IAPP 41.9 ± 1.1%; Spike 48.3 ± 0.9%; N-CTD 35.9 ± 0.7%). In contrast, proteins alone were largely noncytotoxic (spike 116.9 ± 2.9%; N-CTD 128.0 ± 2.3% viability; LDH 37.6 ± 0.2% and 33.7 ± 0.2%, respectively), whereas co-application with IAPP peptides decreased viability and increased LDH (IAPP+spike 32.4 ± 1.0% viability; LDH 70.0 ± 0.5%; adjusted *p* <0.01 vs the corresponding single protein).

IAPP coexistence with viral proteins depleted intracellular insulin under both delivery modes. In vector experiments, the intracellular insulin AUC fell from 10,173 ± 188 a.u. (control) to 5174 ± 152 a.u. with IAPP+Spike (≈50.9% of control; adjusted *p* = 2.7 × 10^−4^ vs control; *p* = 0.0006 vs IAPP alone; *p* = 0.0139 vs Spike alone) and to 5887 ± 123 a.u. with IAPP + N-CTD ( ≈ 57.9% of control; adjusted = 6.4 × 10^−4^ vs control; *p* = 0.0031 vs IAPP alone; *p* = 0.0014 vs N-CTD alone) (Fig. 5d). In protein experiments, Supplementary Fig. 1f demonstrates the efficient cellular internalization of extracellularly administered Spike protein (f1) and N-CTD protein (f2) for 24 h by MIN6 β-cells. IAPP+Spike (10 µg/mL) lowered intracellular insulin to 5406 ± 218 a.u. (53.1% of control; adjusted *p* = 0.0022 vs control; *p* = 0.0001 vs spike protein), whereas IAPP + N-CTD (10 µg/mL) was 88.3% of control (8985 ± 74 a.u.; adjusted *p* = 0.0289 vs control; *p* = 0.0236 vs N-CTD protein; *p* <0.0001 vs IAPP alone) (Fig. 5h).

Time-course assays showed two secretion peaks: glucose-stimulated insulin secretion (GSIS) and KCl-stimulated insulin secretion (KSIS). In vector experiments, GSIS decreased from 976 ± 29 a.u. (control) to 526 ± 8 a.u. (IAPP+Spike) and 509 ± 7 a.u. (IAPP + N-CTD) (adjusted *p* = 0.012 and 0.010 vs control), while KSIS decreased from 1214 ± 39 a.u. (control) to 435 ± 13 a.u. (IAPP+Spike) and 307 ± 12 a.u. (IAPP + N-CTD) (adjusted *p* = 0.005 and 0.005 vs control; *p* = 0.020 vs IAPP alone and *p* = 0.0048 vs N-CTD alone, respectively) (Fig. 5e). Aggregate secretion (AUC) was reduced to 1287 ± 28 a.u. with IAPP+Spike (43.2% of control) and 1044 ± 23 a.u. with IAPP + N-CTD (35.1% of control; both adjusted *p* ≤ 0.0029 vs control; significant vs corresponding single vectors) (Fig. 5f).

Protein experiments recapitulated these trends with lower basal toxicity. GSIS fell from 976 ± 29 a.u. (control) to 589 ± 26 a.u. (IAPP+Spike) and 660 ± 17 a.u. (IAPP + N-CTD) (adjusted *p* = 0.0039 and 0.0064 vs control; significant vs the corresponding single proteins). KSIS declined from 1214 ± 39 a.u. to 304 ± 15 a.u. (IAPP+Spike) and 307 ± 7 a.u. (IAPP + N-CTD) (adjusted *p* = 0.0022 and 0.0042 vs control; *p* ≤ 0.0011 vs the corresponding single proteins) (Fig. 5i). The secretion AUC mirrored these deficits (IAPP+Spike 1182 ± 43 a.u., IAPP + N-CTD 1334 ± 26 a.u.; 39.7% and 44.8% of control; adjusted *p* ≤ 0.0023 vs control) (Fig. 5j).

Notably, EV insulin cargo (vector mode) was selectively depleted in the IAPP + N-CTD group (1163 ± 24 a.u.) compared with the control (2764 ± 25 a.u.; adjusted *p* < 0.0001) and with N-CTD alone (3307 ± 83 a.u.; adjusted *p* < 0.0001). EV-insulin in IAPP+Spike did not differ from the control (2835 ± 22 vs 2764 ± 25 a.u.; adjusted *p* = 0.21) (Fig. 5g). Consistent with the ultrastructural data, all treatment groups released ~10-fold more EVs than the control by nanoparticle tracking analysis (e.g., control 5.37 × 10⁸ vs 5.82–8.44 × 10⁹ particles/mL across treatment groups; median diameters 77.8–83.8 nm), while TEM confirmed typical cup-shaped vesicles (Fig. 5l, m, Supplementary material of raw size distribution and concentration profiles of exosomes). Together, these data indicate EV hypersecretion under stimulated conditions with inadequate insulin packaging, particularly when IAPP and N-CTD coexist. We treat these vesicle findings as supportive but not standalone evidence for insulin secretion mechanisms.

### SARS-CoV-2 S/N proteins exacerbate β-cell dysfunction in human-IAPP transgenic islets ex vivo

**Fig. 6:**
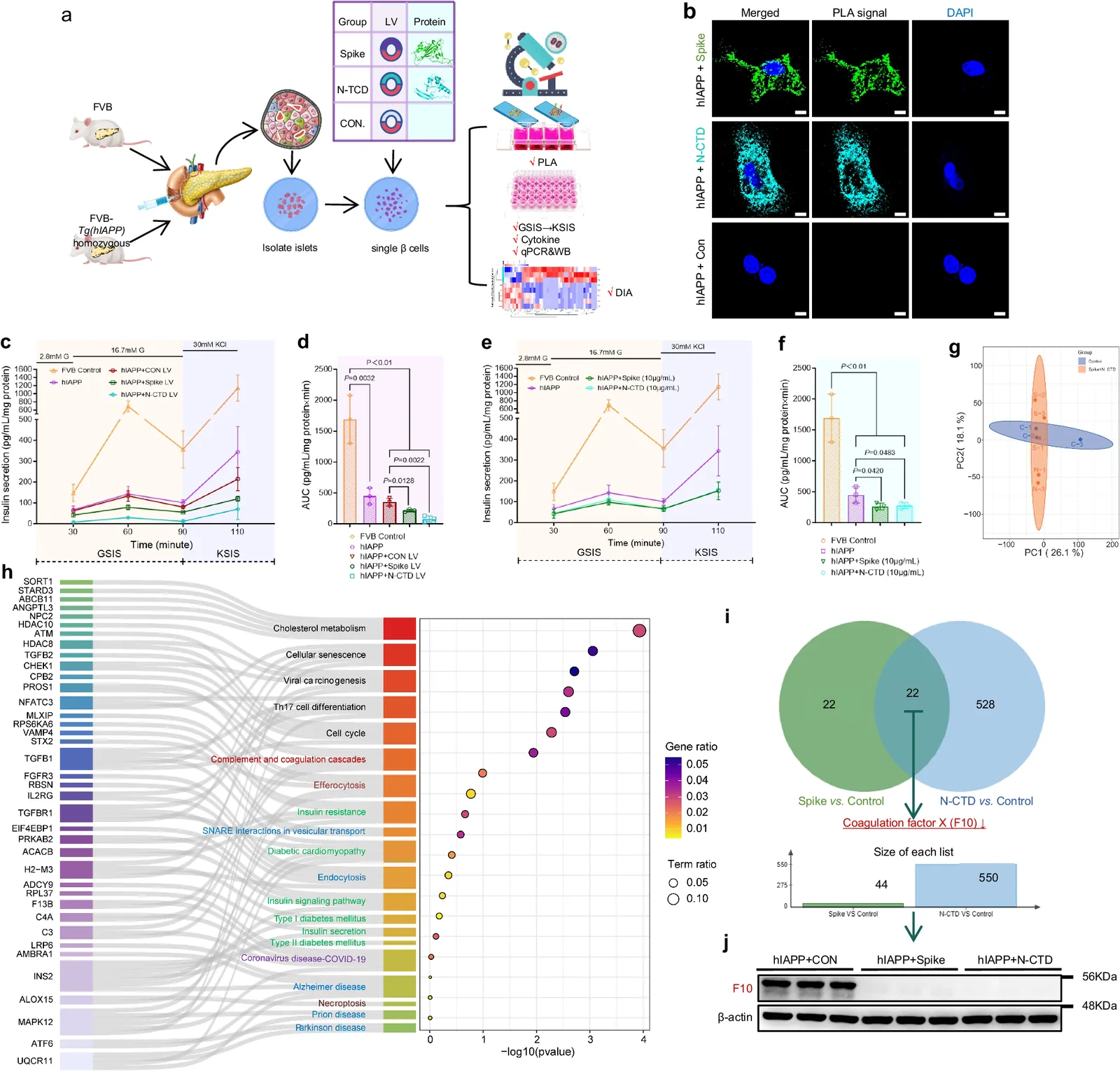
SARS-CoV-2 Spike and N protein exacerbate β-cell dysfunction in human-IAPP transgenic mouse islets (ex vivo validation). **a** Schematic overview of the ex vivo experimental design in islets isolated from hIAPP transgenic mice. **b** Proximity ligation assay (PLA) detecting IAPP-Spike and IAPP-N interactions in islets: Representative images of Duolink PLA in islets isolated from hIAPP transgenic mice after 48 h of infection with spike lentivirus (LV) or N LV. Green or cyan fluorescent spots indicate the IAPP protein in close proximity to the spike or N protein. Control islet: essentially no PLA signal (no viral protein present). Spike-treated islets: numerous red PLA puncta within β-cells (circled), indicating Spike–IAPP complexes. N-treated islet: similarly shows multiple PLA foci per cell. Nuclei counterstained with DAPI (blue). Scale, 50 μm. **c**, **d** Impaired insulin secretion in primary islets by Spike/N LV (**c**). AUC analysis of insulin secretion induced by GSIS (30–90 min) and KSIS (90-110 min) shown in (**d**). Data from three independent experiments, nine mice. **e**, **f** Spike or N protein incubation in islets isolated from hIAPP mice significantly blunted the insulin release capacity. AUC analysis of insulin secretion induced by GSIS (30–90 min) and KSIS (90–110 min) shown in (**f**). Data from three independent experiments, nine mice. **g** Principal component analysis (PCA) of proteomic datasets demonstrates distinct clustering of control islet samples separately from S or N-protein-incubated islet samples, indicating significant proteomic alterations induced by viral proteins. **h** Proteomic pathway analysis of spike/N-treated islets: Sankey diagram illustrating key pathways enriched among proteins changed in spike/N-treated islets vs control islets. Notable enriched pathways include insulin secretion, SNARE interactions, complement cascade, etc. Arrows flow from common proteins to the pathways (right) to which they contribute. The width of the flow corresponds to the number of shared proteins. The dot plot shows the significance and ratio of affected proteins for each pathway. **i** The Venn diagram depicts the intersecting proteins between the differentially expressed proteins of group S and the control group, as well as those of group N and the control group, indicating 22 shared proteins with biomarker potential. **j** Primary islet Western blot analysis of F10 expression was performed to validate the co-downregulated proteins identified by bioinformatics analysis. β-actin was used as a loading control

To further validate these findings in a more physiological setting, we isolated primary islets from FVB background hIAPP-overexpressing homozygous mice and exposed them ex vivo to either lentiviral vectors (LV) encoding Spike or N-CTD or to purified proteins (10 µg mL^−1^) (workflow in Fig. 6a). These mice are known to develop islet amyloid as they age or under metabolic stress, mimicking human type 2 diabetes pathology.^21,22^ We isolated primary islets from adult hIAPP transgenic mice and treated them with SARS-CoV-2 proteins to observe the effects on insulin secretion and β-cell health.

In situ proximity ligation assays (PLA) in primary hIAPP islets visually confirmed the direct intracellular binding of viral proteins to IAPP ( < 40 nm proximity) (Fig. 6b). This provides visual evidence that IAPP and viral proteins physically interact within primary β-cells, consistent with our co-IP results in cell lines and the co-localization in monkey islets.

In LV experiments, GSIS fell from 133.5 ± 14.1 a.u. in hIAPP+CON LV to 78.8 ± 6.7 a.u. with hIAPP+Spike LV (adjusted *p* = 0.043; ~40% reduction) and to 29.8 ± 1.7 a.u. with hIAPP+N-CTD LV (adjusted *p* = 0.0339; ~78% reduction). KSIS likewise decreased from 214.2 ± 32.1 a.u. (hIAPP+CON LV) to 119.8 ± 6.7 a.u. (Spike; adjusted *p* = 0.093, trend) and 70.2 ± 28.5 a.u. (N-CTD; adjusted *p* = 0.058, trend) (Fig. 6c). Aggregate secretion (AUC) was reduced from 351.7 ± 37.2 a.u. (hIAPP+CON LV) to 215.0 ± 12.7 a.u. (Spike; adjusted *p* = 0.0128) and 80.3 ± 28.7 a.u. (N-CTD; adjusted *p* = 0.0022) (Fig. 6d). For reference, wild-type FVB control islets displayed far larger GSIS/KSIS responses (GSIS 689.1 ± 77.3 a.u., KSIS 1141.4 ± 185.1 a.u.), highlighting the functional gap between healthy and hIAPP-sensitized islets (Fig. 6d).

Short-term protein exposure (10 µg mL^−1^, ex vivo) reproduced the direction of the LV results with lower toxicity. GSIS decreased from 142.9 ± 21.3 a.u. in hIAPP islets to 97.7 ± 8.2 a.u. (hIAPP+Spike protein) and 109.4 ± 13.0 a.u. (hIAPP+N-CTD protein); KSIS dropped from 344.4 ± 69.8 a.u. (hIAPP) to 152.4 ± 24.8 a.u. (Spike) and 156.3 ± 19.0 a.u. (N-CTD) (Fig. 6e). AUC values showed the same pattern (hIAPP, 449.7 ± 75.9 a.u.; spike, 261.7 ± 31.5 a.u.; N-CTD, 274.0 ± 28.9 a.u.) (Fig. 6f). The effect sizes (GSIS 0.68× and 0.77×; KSIS 0.44–0.45× of hIAPP) were consistent with the LV data.

To gain insight into molecular changes, we performed a microscale data-independent acquisition (DIA) proteomic analysis on the islets after treatment. DIA proteomics of primary islets revealed clear group separation by PCA and pathway-level remodeling under both Spike and N-CTD (vs control) (Fig. 6g). Enrichment analysis highlighted “insulin secretion”, “SNARE interactions in vesicular transport”, “endocytosis” and “complement and coagulation cascades” among the top altered pathways (Fig. 6h). A Venn diagram indicated a small overlap between the spike- and N-driven proteomes, with coagulation factor X (F10) among the shared hits, pointing to convergent complement/coagulation activation alongside secretory-trafficking defects (see Sankey map, Fig. 6h, i). Western blot analysis of primary islets from matching experimental groups validated the significant downregulation of F10 protein expression initially identified by proteomic screening (Fig. 6j; Supplementary Fig. 7, original data). This suggests that the mechanistic cascade triggered by IAPP-virus interaction is conserved across species: it includes activation of innate immune/complement responses and suppression of insulin vesicle trafficking proteins.

Across PLA, secretion kinetics, and unbiased proteomics, SARS-CoV-2 S/N exacerbates the β-cell vulnerability created by human IAPP, producing high-confidence intracellular IAPP-viral protein complexes, depressed GSIS/KSIS, and proteomic signatures consistent with SNARE-mediated granule transport impairment and complement/coagulation activation. These ex vivo data in hIAPP transgenic islets reinforce the MIN6 phenotypes and align with the structural interfaces defined for S/N-IAPP, providing an integrated mechanistic link between viral exposure and IAPP-driven secretory failure.

### SARS-CoV-2 infection reprograms the aged primate islet proteome: complement activation and insulin vesicle trafficking impairment

**Fig. 7:**
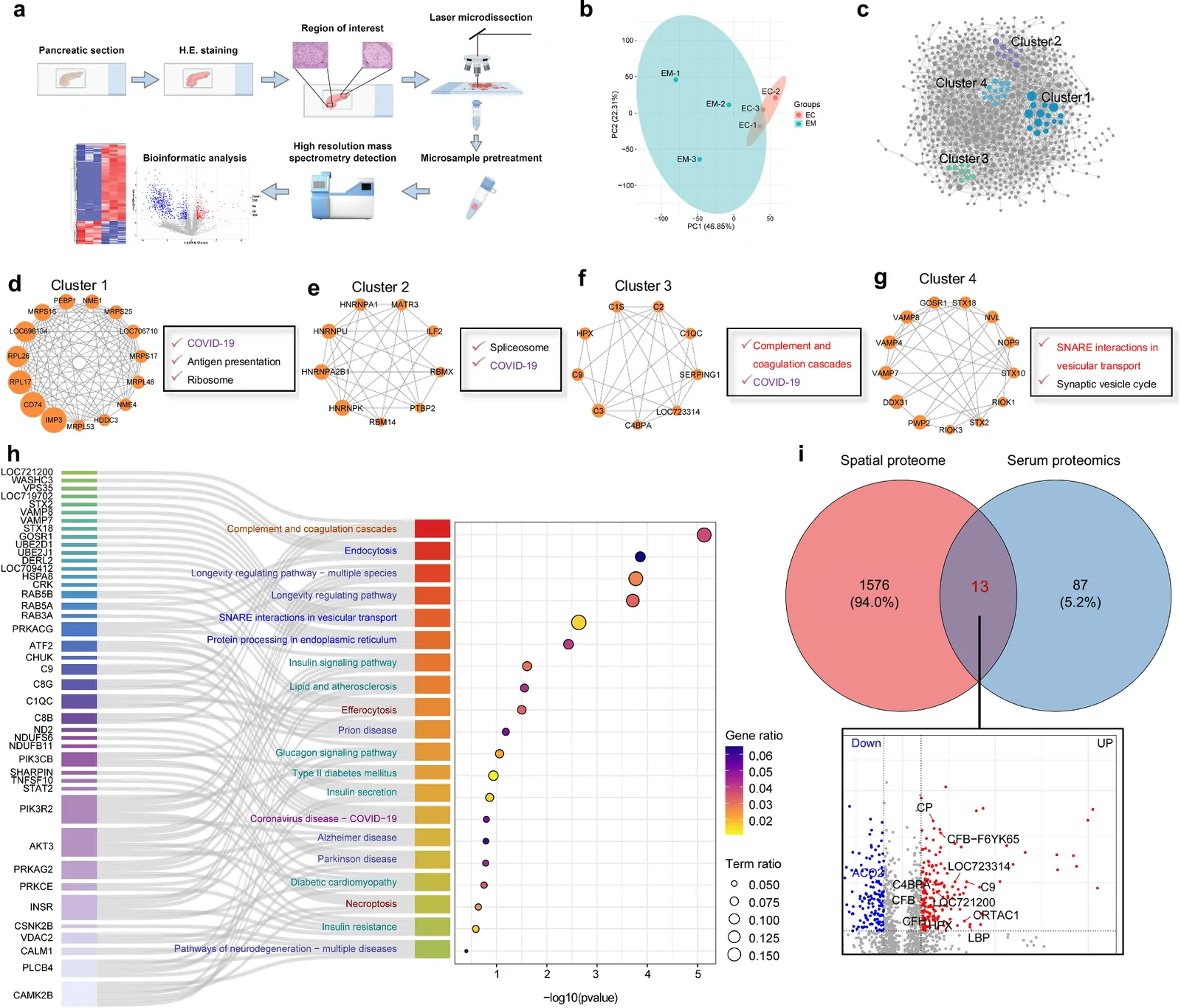
Spatial proteomics reveals key pathways altered in pancreatic islets of SARS-CoV-2-infected elder macaques. **a** Workflow overview illustrating the pipeline from pancreatic tissue preparation, H&E staining, laser microdissection of islet regions of interest, microsample pretreatment, and high-resolution mass spectrometry (MS)-based proteomic analysis to subsequent bioinformatic evaluation. **b** Principal component analysis (PCA) of proteomic datasets demonstrates distinct clustering of elder model (EM) islet samples separately from elder control (EC), indicating significant proteomic alterations induced by SARS-CoV-2 infection. **c** The protein‒protein interaction (PPI) network shows four distinct clusters of highly interconnected proteins. **d**–**g** Cluster enrichment analysis links dysregulated proteins to ribosome/antigen presentation (**d**), spliceosome (**e**), complement activation (**f**), and SNARE vesicle trafficking (**g**). **h** Combined use of the Sankey diagram and dotplot diagram in KEGG pathway analysis identifies altered pathways, including complement activation, vesicular trafficking, ER stress, necroptosis, COVID-19, insulin signaling, insulin resistance, type II diabetes mellitus and neurodegeneration. **i** Venn diagram and volcano plot comparing islet and serum proteomics reveal 13 shared dysregulated proteins with biomarker potential

To globally map the in vivo microenvironmental shift, laser-capture microdissection and LC‒MS/MS spatial proteomics were performed on isolated primate islets (workflow in Fig. 7a).

Principal component analysis revealed profound proteomic divergence post-infection (Fig. 7b), with 184 proteins upregulated and 580 downregulated (Supplementary Fig. 3a). Unsupervised hierarchical clustering of differentially expressed proteins highlighted global downregulation across most islet proteins, with only a subset being upregulated (Supplementary Fig. 3b). To identify regulatory hubs, we performed protein–protein interaction (PPI) network analysis. Four clusters of tightly interconnected proteins exhibited coordinated expression changes (Fig. 7c). Cluster 1 was enriched in proteins involved in ribosome function, COVID-19, and antigen presentation (Fig. 7d). RPL26, a large ribosomal subunit protein, plays a role in ER protein homeostasis and is regulated via UFMylation.^23^ CD74, which is involved in MHC-mediated antigen presentation, has been linked to disease severity in COVID-19 patients.^24^ Cluster 2 was enriched for spliceosome and COVID-19-related proteins (Fig. 7e). SARS-CoV-2 may interfere with mRNA splicing, leading to aberrant inflammatory responses. HNRNPK, a hub in SARS-CoV-2–host interactions, is critical in RNA processing and immune modulation.^25^ Cluster 3 involved complement and coagulation proteins (Fig. 7f). Clinical evidence supports complement overactivation as a key driver of inflammation and organ damage in severe COVID-19.^26^ We confirmed the islet-enriched activation of complement, including upregulation of C1s and deregulation of C4BPA. Cluster 4 was enriched for proteins involved in SNARE-mediated vesicular transport and synaptic vesicle cycling (Fig. 7g), which are critical for insulin granule exocytosis. Disruption of this pathway likely underlies defective insulin release in infected islets. KEGG pathway-enrichment analysis identified the top 20 pathways (*P* < 0.05), visualized using Sankey diagrams and dot plots (Fig. 7h).

Notably, pathways related to metabolism and hormone secretion were heavily affected. This downregulation of INSR (log_2_FC = −7.45, *P* = 0.0037) could compound the metabolic disturbance by making tissues less responsive to insulin, in addition to reduced insulin secretion—“a double hit” impairing blood glucose regulation. Complement and coagulation cascades were strongly enriched among the upregulated proteins (Fig. 7h, cluster 3). We found 14 complement-related proteins that were significantly elevated in infected islets, including components of the classical pathway (C1q, C1s, C2, C4BPA), alternative pathway (CFB, CFH), and terminal pathway (C3, C8, C9). This indicates robust local complement system activation in islets due to infection. Protein processing in the ER, endocytosis, and vesicular transport (SNARE interactions) were enriched among downregulated proteins (Fig. 7h, cluster 4). Many proteins involved in the secretory pathway were decreased, suggesting a disruption in insulin synthesis, folding, and the vesicle trafficking system. Specifically, the SNARE complex pathway had six key proteins downregulated (GOSR1, STX18, STX2, VAMP4, VAMP7, and VAMP8). These SNARE proteins are crucial for vesicle fusion, either for secretion or for lysosomal targeting of granules. Their coordinated downregulation points to a significant impairment in vesicle handling and exocytosis in infected islets.

Interestingly, many differentially expressed proteins were also associated with pathways of chronic neurodegenerative diseases such as prion disease, Parkinson’s disease, and Alzheimer’s disease (Fig. 7h). The list of proteins overlapping these pathways (e.g., MAPT/tau, APOE, LRP1, certain subunits of mitochondrial complexes and proteasome) implies that SARS-CoV-2 infection may cause islet cells to experience molecular changes akin to those seen in protein-misfolding diseases. This raises the question of whether viral infection accelerates common pathological protein aggregation processes across different organs (a concept we explore in the Discussion).

Next, we compared the islet proteome changes with our previously obtained serum proteomics from the same monkeys (aged control vs infected serum). A Venn diagram revealed 13 proteins with consistent trends in both serum and islets, including upregulated ceruloplasmin (CP), CABPA, hemopexin (HPX), CFH, CFB, LBP, C2, C9, and CRTAC1 and downregulated ACO2 (Fig. 7i). These findings underscore systemic changes reflected in local islet environments and identify potential circulating biomarkers of virus-induced islet injury. Most of these proteins are associated with complement activation, reinforcing the central role of innate immune dysregulation in SARS-CoV-2-induced islet pathology. We subsequently compared the proteomic profiles of isolated islets with the spatial proteomics data from monkey pancreatic sections. Venn diagram analysis revealed two overlapping proteins that exhibited consistent regulation patterns: F10, which was significantly downregulated, and C3, which was significantly upregulated (Supplementary Fig. 3c). Both of these proteins are closely implicated in the activation of the complement system.

Overall, the pathway analysis paints a picture of SARS-CoV-2 turning islets into an environment of metabolic dysfunction (downstream insulin signaling compromised), innate immune activation (complement up), and protein-trafficking stress (ER and vesicle pathways down), which together would severely perturb β-cell function.

### Complement system activation and inflammatory response in the infected islets microenvironment

To further validate the bioinformatics findings from our spatial proteomics analysis regarding complement system activation in the islet microenvironment, we used Pathview to generate KEGG pathway-enrichment maps. By mapping differential protein expression data onto the topological structure of the “complement and coagulation cascades” pathway, we visualized the distribution and regulation of related proteins. Upregulated proteins are shown in red, and downregulated proteins in green. This visualization revealed a pronounced activation trend in the complement pathway following SARS-CoV-2 infection in aged monkeys (Supplementary Fig. 4), with key components including MAC, C1, C3, C4, C6/7/8/9, C4BPA, factor B (FB), and factor H (FH) being upregulated, while only F10 was significantly downregulated.

**Fig. 8:**
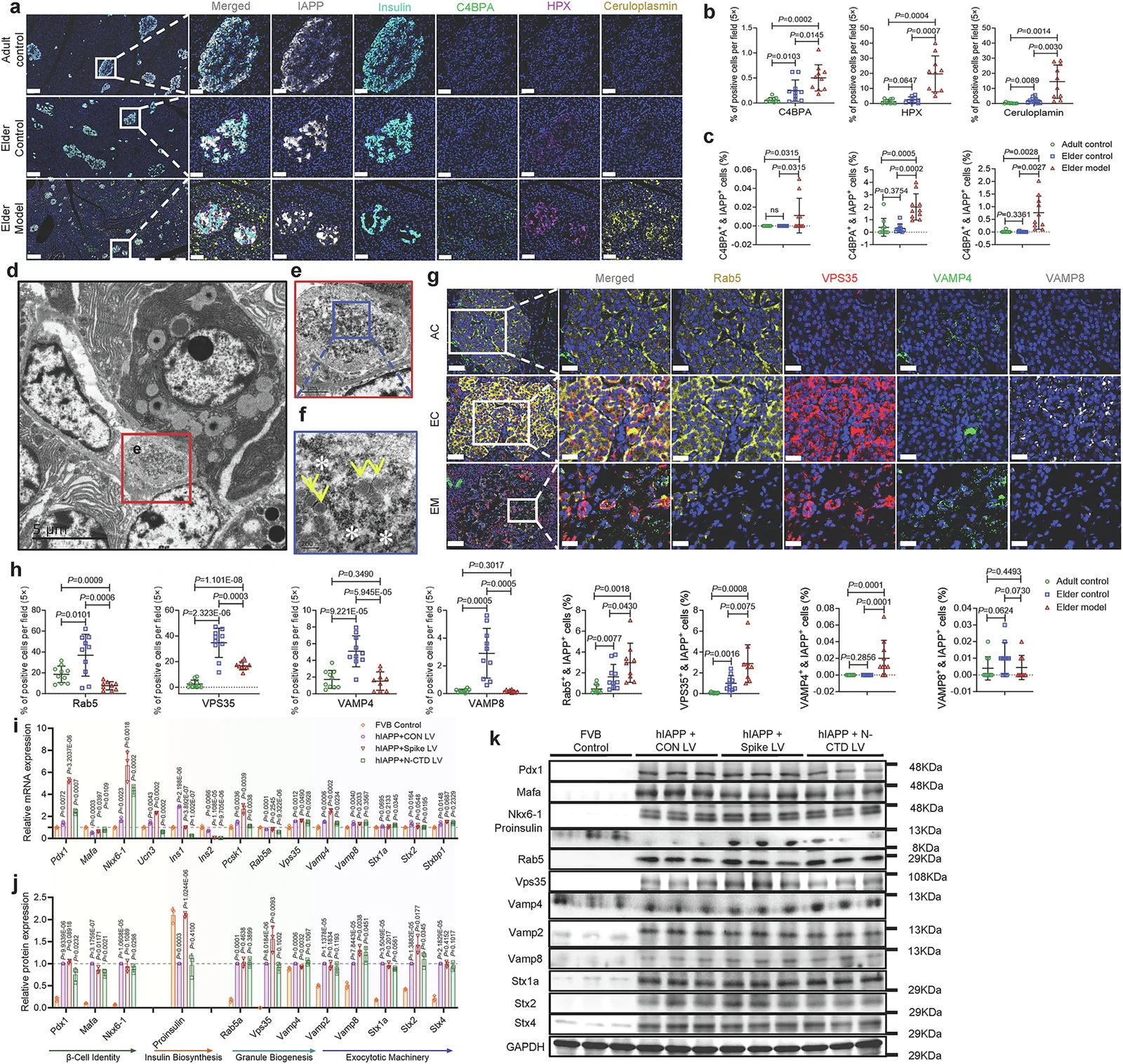
SARS-CoV-2 infection impairs insulin maturation, inhibits SNARE-mediated vesicular trafficking, and promotes complement activation in vivo and ex vivo. **a** Multiplex immunofluorescence of islets from adult control (AC), elder control (EC), and infected elder model (EM) macaques stained for IAPP (white), insulin (cyan), and acute-phase/complement-associated proteins: C4BPA (green), HPX (magenta), and ceruloplasmin (yellow). Enlarged inset boxes demonstrate marked accumulation of complement activation-associated proteins in EM islets. Low-magnification image with scale bar, 100 μm. Insets show enlarged views, Scale bars, 20 µm. **b**, **c** Quantitative analysis of fluorescence intensity (% positive area and % positive cells) confirms significant upregulation of C4BPA, HPX, and ceruloplasmin in EM animals compared with AC and EC animals. (×20 magnification; *n* = 10 images examined in total/group). **d**–**f** TEM images of EM islets show dilated vesicles and vesicular entrapment within the fibrotic matrix. Scale bars: **d** 5 µm; **e** 1 µm; **f** 200 nm. **g** Multiplex immunofluorescence staining of vesicular trafficking proteins Rab5 (orange), VPS35 (red), VAMP4 (green), and VAMP8 (white) across adult control (AC), elder control (EC), and elder model (EM) groups. Boxed regions in merged images are magnified to the right, illustrating altered expression and disrupted distribution patterns in EM islets. Scale bars, 20 µm. **h** Quantitative analysis showing the percentages of cells positive for Rab5, VPS35, VAMP4, and VAMP8, as well as their colocalization with IAPP, highlighting significant reductions in Rab5 and VPS35 expression and changes in VAMP4/VAMP8 distribution in EM islets (×20 magnification; *n* = 10 images examined in total/group). Statistical analysis: 10 images from 3–4 animals per group were quantified. Measurements from the same animal were averaged, and animal-level means were used for statistical testing. Data are presented as the mean ± SD. *p* values were calculated by unpaired two-tailed Student’s *t* test: *p < 0.05; **p < 0.01; ***p < *0.001*; ns not significant. **i**–**k** qPCR and immunoblot analyses of β-cell identity markers (*Pdx1, Mafa, Nkx6-1*, and *Ucn3*), insulin mRNA (*Ins1* and *Ins2*), granule biogenesis (*Pcsk1*), granule trafficking (*Rab5a, Vps35*, and *Vamp4*), exocytosis (*Vamp2, Vamp8, Stx1a, Stx2, Stx4*, and *Stxbp1*), and insulin precursor proteins in FVB and hIAPP islets following lentiviral transduction with Spike or Nucleocapsid. GAPDH was used as a loading control. mRNA and protein levels were quantified and normalized to GAPDH. mRNA expression data are presented relative to the FVB control group; protein expression data are presented relative to the hIAPP + control LV group. Data are shown as the mean ± SD from three independent experiments (*n* = 3). Statistical significance was determined using an unpaired two-tailed Student’s *t* test: *p < 0.05, **p < 0.01, ***p < 0.001; ns not significant

To validate these findings, we performed multiplex immunofluorescence to examine the in situ expression of three representative proteins enriched in both serum and islet proteomic datasets: C4BPA, HPX, and ceruloplasmin (Fig. 8a). C4BPA is a negative regulator of the classical pathway, attenuating complement activity and limiting inflammation.^27^ HPX is a plasma protein that binds free heme, mitigating oxidative stress and inflammation.^28^ Ceruloplasmin is a copper-binding α₂-glycoprotein involved in iron metabolism and antioxidant defense, with roles in modulating immune responses. Under normal physiological conditions, these proteins play protective roles in host tissues.^29^ Quantitative immunofluorescence analysis revealed the following: (a) In adult monkeys, the expression of C4BPA, HPX, and ceruloplasmin in pancreatic islets was minimal (0.056%, 1.256%, and 0.250%, respectively). (b) In aged controls, C4BPA (0.247%, *P* = 0.0102) and ceruloplasmin (1.96%, *P* = 0.00886) were significantly elevated, while HPX showed a non-significant upward trend (2.455%, *P* = 0.0647). However, the number of double-positive cells co-expressing IAPP and these proteins (e.g., C4BPA^+^IAPP^+^, HPX^+^IAPP^+^, Ceruloplasmin^+^IAPP^+^) did not differ significantly between the adult and aged groups, suggesting that in the aging islet environment, higher expression of protective proteins serves to maintain local homeostasis independently of IAPP aggregation. (c) In the aged model group, despite the expression levels of all three proteins being significantly elevated compared to both adult and aged control groups (0.502%, 19.608%, and 14.46%, respectively), the numbers of double-positive cells for co-localized proteins C4BPA^+^IAPP^+^ (0.01%), HPX^+^IAPP^+^ (2.023%), and Ceruloplasmin^+^IAPP^+^(0.76%) were increased. Notably, the double-positive cell counts for HPX^+^IAPP^+^ (*P* = 0.000245 vs. adult group; *P* = 0.000462 vs. aged group) and Ceruloplasmin+IAPP+ (*P* = 0.00270 vs. adult group; *P* = 0.00282 vs. aged group) were significantly higher than those in both the adult and aged control groups (Fig. 8b, c). This suggests that although protective proteins are upregulated during infection in an attempt to counter local inflammation, they may be sequestered by pathological IAPP aggregates, rendering their protective effects ineffective.

Complement overactivation is known to promote cellular stress and protein misfolding, exacerbating inflammation and organ dysfunction in COVID-19.^30^ We next assessed markers of endoplasmic reticulum stress (ERS) and inflammation within islets using multiplex immunofluorescence: CHOP (also known as DDIT3) and GRP78 (BiP/HSPA5) as ER stress markers and IL-6 and TNF-α as key pro-inflammatory cytokines (Supplementary Fig. 5a). a. In adult monkeys, the baseline expression of CHOP (0.02%), GRP78 (0.00%), IL-6 (0.33%), and TNF-α (1.07%) was minimal. b. In aged controls, CHOP (0.05%, *P* = 0.0589) and GRP78 (0.004%, *P* = 0.0998) were slightly elevated without statistical significance. IL-6 (0.900%, *P* = 0.00275) and TNF-α (2.92%, *P* = 0.00464) showed significant increases, yet double-positive cells co-expressing IAPP and these markers were unchanged—except for a slight increase in TNF-α^+^IAPP^+^ cells (0.107%, *P* = 0.0187), suggesting a mild chronic inflammatory state in the aging islet microenvironment. c. In the infected aged group, GRP78 (0.06%), IL-6 (10.18%), and TNF-α (8.71%) were significantly upregulated compared to controls, while CHOP (0.040%) remained similar to the aged group but was significantly higher than in adults. Colocalization analysis showed marked increases in CHOP^+^IAPP^+^ (0.055%), GRP78^+^IAPP^+^ (0.020%), IL-6^+^IAPP^+^ (1.128%), and TNF-α^+^IAPP^+^ (2.380%) double-positive cells (Supplementary Fig. 5b, c).

In diabetes, CHOP mediates β-cell apoptosis and contributes to insulin secretion defects.^31^ GRP78 is involved in β-cell function and insulin resistance.^32^ IL-6 and TNF-α are central inflammatory mediators^33^; in COVID-19, they fuel cytokine storms and immune dysregulation, while in diabetes, they promote chronic inflammation and metabolic imbalance, accelerating β-cell injury and insulin dysfunction. These findings are consistent with our spatial proteomics data, suggesting that ER stress in β-cells may result in ribosome stalling, further exacerbating β-cell demise.

To further investigate whether IAPP-viral protein co-exposure augments IL-6 and TNF-α production in β-cells, we quantified IL-6 and TNF-α in MIN6 cultures under the same perturbations used for GSIS/KSIS. Control cultures produced 1.06 ± 0.27 a.u. IL-6. The copresence of IAPP with viral proteins markedly increased IL-6: a. (IAPP + N-CTD) vector: 5.11 ± 0.49 a.u. (4.84× vs control; adjusted *p* = 0.030); b. (IAPP+Spike) vector: 4.64 ± 0.40 a.u. (4.40×; *p* = 0.023); c. N-CTD vector: 3.53 ± 0.31 a.u. (3.34×; *p* = 0.030); d. IAPP + N-CTD protein (10 μg mL^−1^): 5.67 ± 0.39 a.u. (5.37×; *p* = 0.010) (Supplementary Fig. 5d). Other conditions showed similar directions (e.g., IAPP+Spike protein 4.11 ± 0.53 a.u., 3.89×; *p* = 0.062, trend). Notably, combinations tended to exceed single treatments: (IAPP+Spike) vector > IAPP (*p* = 0.0039) and > Spike (*p* = 0.0096); IAPP + N-CTD protein > N-CTD protein (*p* = 0.0012) (Supplementary Fig. 5d). For TNF-α, the control was 1.18 ± 0.35 a.u. IAPP alone elevated TNF-α (6.72 ± 0.19 a.u., 5.69×; adjusted *p* = 0.006) (Supplementary Fig. 5d). The strongest increases occurred when IAPP and Spike were combined: a. (IAPP+Spike) vector: 10.27 ± 1.35 a.u. (8.69×; unadjusted *p* = 0.016); b. IAPP+Spike protein: 10.43 ± 1.15 a.u. (8.83×; unadjusted *p* = 0.0099; vs Spike protein *p* = 0.0028). c. (IAPP + N-CTD) vector also increased (7.97 ± 0.85 a.u., 6.75×; unadjusted *p* = 0.0077) (Supplementary Fig. 5e). These cytokine changes parallel the depletion of insulin stores and blunted GSIS/KSIS described for the same MIN6 conditions, indicating a coupled secretory-inflammatory phenotype when IAPP and S/N co-exist.

We next examined cytokines in islets isolated from FVB hIAPP-Tg homozygotes (ex vivo) exposed to lentiviral S/N or purified proteins. With lentiviral delivery, the hIAPP+CON LV produced 0.75 ± 0.07 a.u. IL-6. hIAPP+N-CTD LV increased IL-6 to 1.37 ± 0.09 a.u. (1.83×; adjusted *p* = 0.0026), while hIAPP+Spike LV reached 1.12 ± 0.13 a.u. (1.49×; *p* = 0.0310). For short-term exposure, hIAPP+N-CTD protein levels rose to 1.13 ± 0.05 a.u. (2.38× vs hIAPP 0.48 ± 0.06 a.u.; adjusted *p* = 0.0005), and hIAPP+Spike protein levels reached 1.50 ± 0.26 a.u. (3.15×; *p* = 0.0088) (Supplementary Fig. 5f). Of note, wild-type FVB islets showed much lower IL-6 (0.11 ± 0.05 a.u., 0.22× vs hIAPP; *p* = 0.0044), underscoring the pro-inflammatory baseline in hIAPP-sensitized islets. For TNF-α, hIAPP+CON LV measured 1.36 ± 0.18 a.u.; hIAPP+N-CTD LV increased TNF-α to 3.72 ± 0.60 a.u. (2.73×; unadjusted *p* = 0.0100), and hIAPP+Spike LV to 2.09 ± 0.25 a.u. (1.53×; *p* = 0.0382). Protein exposure reproduced the direction with greater variability (hIAPP+Spike protein 4.99 ± 1.88 a.u., 3.70× vs hIAPP; adjusted *p* = 0.0631; hIAPP+N-CTD protein 1.95 ± 0.83 a.u., 1.45× vs hIAPP; *p* = 0.0019). FVB islets remained low (0.25 ± 0.06 a.u., 0.18× vs hIAPP; *p* = *0.0046*) (Supplementary Fig. 5g).

Across MIN6 and hIAPP transgenic islets, IAPP together with S/N elevates IL-6 and TNF-α in parallel with depressed insulin content, impaired GSIS/KSIS, and EV hypersecretion. The strongest cytokine induction is observed under IAPP + N-CTD (IL-6) and IAPP+Spike (TNF-α) conditions, matching the direct S/N-IAPP interfaces and SNARE/complement pathway remodeling defined in Figs. 3–7. This supports a model in which viral S/N proteins exacerbate IAPP-driven β-cell stress, combining secretory pathway failure with a pro-inflammatory milieu that likely amplifies β-cell dysfunction.

In summary, key components of the complement system (e.g., C1S, C2, C3, C4BPA, SERPING1, and C1QC) are hyperactivated in aged islets following SARS-CoV-2 infection through classical and terminal pathways. This overactivation contributes to β-cell ER stress and inflammatory cytokine production,^34^ exacerbating islet dysfunction in COVID-19.

### Spatial proteomics links IAPP pathology to vesicle trafficking disruption

One of the most intriguing findings from the proteomic analysis was the downregulation of SNARE complex proteins and other vesicle trafficking regulators in infected islets. This suggested a mechanism whereby IAPP aggregation might directly impair the machinery of insulin granule transport and exocytosis. To explore this, we examined key components of the insulin vesicle pathway in pancreatic tissues. To visualize the impact of viral infection on vesicular transport, we used Pathview to map differential protein expression in the KEGG pathway “SNARE interactions in vesicular transport.” The results indicated a broad suppression of key vesicle-associated proteins, including VAMP4, VAMP7, and VAMP8, in SARS-CoV-2-infected aged monkeys (Supplementary Fig. 6a).

TEM revealed a marked reduction in insulin granules, which are typically encased by a single membrane, in infected β-cells, with many appearing as membrane remnants. Many granule “ghosts” with only a single limiting membrane remained, lacking dense cores, indicating degranulation (Fig. 8d). High-magnification views of the peri-islet capillary basement membrane revealed dense fibrous material that appeared to trap vesicles (yellow arrows), accompanied by membrane debris and cellular fragments scattered between the fibers (white asterisks) (Fig. 8e, f and Supplementary Fig. 5h). This provides a direct visual correlate to the biochemical finding that SNARE-mediated trafficking is impaired: IAPP aggregates physically obstruct vesicular transport routes in the islet.

Rab5 and VPS35 are essential regulators of endocytosis and membrane-trafficking, maintaining intracellular transport homeostasis in β-cells. VAMP4 forms part of the SNARE complex with STX7, STX8, and VTI1B to mediate lysosomal targeting of insulin granules, helping maintain insulin homeostasis and potentially preventing IAPP aggregation.^35^ VAMP8, in complex with Munc18b and syntaxin-3, facilitates granule fusion and insulin secretion, and its dysfunction is implicated in impaired insulin exocytosis and IAPP aggregation in type 2 diabetes.^36,37^ We next performed multiplex immunofluorescence for VAMP4, VAMP8, Rab5, and VPS35 to assess in situ expression and localization in the pancreas (Fig. 8g): a. Rab5: In adult monkeys, Rab5 expression was uniform in islets (18.57%). Aged monkeys showed significantly elevated expression (36.96%), consistent with enhanced endocytic capacity. However, in SARS-CoV-2-infected aged monkeys, Rab5 expression was sharply reduced (7.62%). Notably, Rab5^+^IAPP^+^ double-positive cells were significantly more abundant in infected monkeys (2.96%) than in adults (0.473%, *P* = 0.00181) and aged controls (1.600%, *P* = 0.0430). b. VPS35: In adult islets, VPS35 expression was low (2.45%) but increased markedly in aged monkeys (34.71%, *P* = 2.3229 × 10^−6^), likely reflecting enhanced retromer complex-mediated trafficking from endosomes to the Golgi or plasma membrane. In the infected aged group, VPS35 expression was significantly downregulated (16.79%) and displayed aggregated distribution patterns. Importantly, VPS35^+^IAPP^+^ cells were more prevalent in the infected group (2.89%) than in adults (0.08%, *P* = 0.000841) and aged controls (0.998%, *P* = 0.00753), suggesting that IAPP aggregation impairs VPS35 function, disrupting intracellular cargo recycling. c. VAMP4: In adult monkeys, VAMP4 expression was low (1.71%) but modestly increased in aged controls (5.09%). Infection led to a significant reduction in expression (1.51%). VAMP4^+^IAPP^+^ cells were significantly more abundant in infected monkeys (1.84%) than in adult (0.033%, *P* = 0.000145) and aged (0.026%, *P* = 0.000141) controls, indicating that IAPP aggregation may sequester VAMP4 and hinder vesicle targeting. d. VAMP8: VAMP8 expression was nearly undetectable in adult monkeys (0.20%) but significantly increased in aged monkeys (2.90%, *P* = 0.0102), consistent with a compensatory mechanism to maintain insulin homeostasis. However, infection sharply suppressed VAMP8 expression (0.17%, *P* = 0.000469), compromising insulin granule fusion and exocytosis (Fig. 8h).

Collectively, the spatial proteomics and follow-up analyses reveal a mechanistic picture wherein SARS-CoV-2 infection in aged islets triggers a cascade: complement system hyperactivation (driving inflammation and cell injury) and concomitant downregulation of SNARE and endosome machinery (blocking insulin granule trafficking).

### Primary islet experiments clarify insulin transcription/processing changes and support granule maturation defects

To explicitly test whether reduced secretion reflects reduced insulin production versus selective loss of exocytic machinery, new primary islet experiments were performed using islets isolated from FVB controls and hIAPP-overexpressing mice, followed by lentiviral transduction with Spike or Nucleocapsid. Comprehensive mRNA (Fig. 8i; Supplementary Table 2) and protein (Fig. 8j, k) profiling confirmed β-cell identity markers (*Pdx1, Mafa, Nkx6-1* and *Ucn3*), assessed insulin mRNA abundance (*ins1* and *ins2*), quantified insulin precursor (proinsulin) accumulation, and measured the expression of granule biogenesis (*Pcsk1*), transport (*Rab5a, Vps35* and *Vamp4*), and exocytosis biomarkers (*Vamp2, Vamp8, Stx1a, Stx2, Stx4* and *Stxbp1*) at both the transcript and protein levels. First, overexpression of the spike or nucleocapsid via adenovirus significantly increased human IAPP expression (Supplementary Fig. 5i). A key new observation was an insulin mRNA stall after spike/nucleocapsid transduction accompanied by compensatory upregulation of multiple trafficking/exocytosis-associated genes at the mRNA level, while many corresponding proteins showed limited change; nevertheless, insulin precursor proteins accumulated, supporting impaired processing/trafficking in the context of IAPP aggregation. Because overall total protein expression remained relatively stable (Supplementary Fig. 7, original western blotting data), this increased proinsulin accumulation proves that the cellular manufacturing machinery (transcription and initial translation) is desperately attempting to function. The functional precursor proteins are successfully synthesized but are immediately subjected to “physical entanglement” and spatial blockade by the rapidly forming, viral-induced IAPP fibril web within the ER/Golgi network. Denied physical transit to the distal secretory vesicles where prohormone convertases (*Pcsk1*) reside, proinsulin cannot be cleaved into mature insulin. Consequently, glucose-stimulated and KCl-stimulated insulin secretion assays (GSIS/KSIS) demonstrated severe secretory blunting. The cells, in distress, release an order-of-magnitude more exosomal vesicles, but due to physical blockade, these vesicles are drastically depleted of their insulin cargo. This explains the mechanical obstruction of functional proteins.

This pattern is consistent with a scenario in which components are expressed but become functionally compromised by IAPP entanglement/sequestration rather than transcriptional silencing.

## Discussion

Our study addresses a central open question in post-COVID-19 metabolic biology: how a transient viral infection can plausibly initiate a durable β-cell failure trajectory. We provide evidence for a mechanism in which SARS-CoV-2 structural proteins directly bind IAPP, accelerate IAPP aggregation, and thereby compromise insulin granule biology in a manner that is markedly more pronounced in aged primate islets. This framework integrates in situ pathology, structural prediction, quantitative binding assays, and functional β-cell/islet data into a coherent axis linking viral exposure to amyloidogenic proteostasis collapse.

The relationship between SARS-CoV-2 infection and amyloid protein aggregation has garnered increasing scientific attention. Beyond its well-documented effects on the respiratory system, SARS-CoV-2 is now recognized to impact multiple extrapulmonary systems, including the CNS, where it may contribute to severe neurodegenerative outcomes. Emerging evidence indicates that SARS-CoV-2 facilitates amyloidogenic processes through direct interactions between its nucleocapsid (N) protein and host stress granules (SGs)—membraneless organelles involved in mRNA regulation during cellular stress. The N protein can insert into SGs and disrupt their dynamic disassembly, thereby promoting persistent aggregation. This interaction is largely driven by liquid–liquid phase separation (LLPS) and is implicated in enhancing amyloid deposition associated with amyotrophic lateral sclerosis (ALS).^38^ In parallel, neurodegenerative disorders such as Alzheimer’s disease (AD) and Parkinson’s disease (PD) are characterized by pathogenic amyloid accumulation that contributes to neuronal damage and cognitive decline. Notably, SARS-CoV-2 infection may accelerate the progression of such diseases by promoting intracerebral amyloid accumulation, potentially explaining some of the neurological symptoms observed in COVID-19 patients.^39^ Once the virus gains access to the brain, it appears to exacerbate amyloid deposition, contributing to cellular toxicity and neurodegeneration. Interestingly, we compared a published PIGs-associated signature (Supplementary Table 3)^40^ with our spatial islet proteome and found APOE as a convergently upregulated factor; STRING-based interaction mapping and KEGG contextualization positioned APOE within networks linked to inflammatory and trafficking modules, nominating it as a potential mediator or biomarker of this injury state (Supplementary Fig. 3d). Importantly, this evidence opens new avenues for therapeutic development, suggesting that targeting viral interactions with amyloidogenic proteins^41^ or modulating phase separation dynamics could mitigate both metabolic^42,43^ and neurodegenerative complications following viral infections such as COVID-19.^15,44^

The primate data also suggest that both direct infection and paracrine/bystander pathways likely contribute. SARS-CoV-2 can involve the exocrine pancreas, microvasculature, and endocrine compartments, and inflammatory mediators from infected exocrine regions can plausibly impair nearby islets through paracrine signaling. Sense/antisense viral RNA localization with quantitative scoring supports focal replication-associated signals and provides a path to reconcile limited islet infection with robust islet injury. Spatial microproteomics and multiplex immunostaining further support local complement activation and cytokine induction within islet regions, offering a mechanistic bridge between infection geography and endocrine dysfunction. Importantly, in β-cell stress states, reduced stimulus-evoked secretion can reflect reduced insulin content, impaired granule maturation, granule mislocalization, defective fusion, or combinations thereof. Our primary islet profiling indicates an insulin mRNA “stall” with compensatory transcriptional responses in trafficking/exocytosis genes, while protein-level changes are comparatively modest, yet insulin precursor proteins accumulate, supporting impaired processing/trafficking in the setting of IAPP aggregation. Together with EM-based granule/vesicle quantification, these data favor a “granule maturation/availability and trafficking disruption” model rather than a selective global collapse of exocytic machinery.

Several limitations are notable. First, while age is a strong modifier in our dataset (minimal adult-infected IAPP pathology versus extensive age-infected plaques), additional controlled comparisons across adult-infected cohorts and timepoints would strengthen causal inference about aging mechanisms. Second, some vesicle markers (e.g., Rab5) can be sensitive to antibody performance and fixation; therefore, we interpret localization patterns cautiously and prioritize convergent evidence from quantitative imaging, qPCR, western blotting, proteomics, and functional readouts. Third, while extracellular vesicle data are consistent with altered vesicle trafficking, we do not rely on them as sole evidence for insulin export mechanisms; instead, we emphasize granule morphology, insulin content, and stimulus responses as field-accepted anchors. An additional limitation is that although the exploratory ITM2B SPR data suggest overlap between viral spike protein and BRICHOS-accessible IAPP interfaces, dedicated cell- or islet-based chaperone rescue experiments will be required to determine whether BRICHOS/ITM2B-related molecules can functionally suppress viral protein-accelerated IAPP toxicity.

In conclusion, we propose that SARS-CoV-2 Spike and Nucleocapsid can hijack IAPP as an amyloidogenic substrate, accelerating toxic assembly and triggering a cascade of complement/inflammatory activation and vesicle trafficking disruption that collectively impairs insulin granule biology. Mapping this highly specific mechanistic axis offers immediate, actionable translational opportunities for managing post-COVID-19 diabetes. By precisely identifying the 5-amino-acid interaction pocket necessary for S/N-IAPP physical binding via our SPR mutant assays, we have highlighted a highly specific, druggable interface. The development of small-molecule inhibitors, rationally designed peptide antagonists, or anti-amyloid chaperone therapies designed to sterically block this 5-amino-acid pocket could prevent the initial viral seeding event.^45^ This mechanism provides a testable framework for understanding age-associated vulnerability to post-infectious metabolic dysfunction and suggests therapeutic opportunities focused on interrupting the viral protein–IAPP interface or stabilizing IAPP proteostasis.

## Materials and methods

### Ethics statement and animal experiments

All animal tests were conducted in an ABSL3 facility using isolators with HEPA filters. All experiments were carried out following the Animal Welfare Act and other related animal and experimental regulations (Protocol Approval Number: DW20010). In this research, adult rhesus macaques (*Macaca mulatta*) were aged between 3 and 5 years, and elderly rhesus macaques were 23–31 years old (the date of birth in months/date/years of elderly animals is listed in the following table). These age ranges align with the typical lifespan and aging process of NHPs and are indicative of adult and elderly stages in human terms.^14^ Research protocols, particularly those involving animals, received approval from the Institutional Animal Care and Use Committee of the Institute of Laboratory Animal Science, Peking Union Medical College. In short, all samples were taken after administering 10 mg/kg ketamine hydrochloride for anesthesia.^10,11,13,14^

**Table:** 

Animal no.	Date of birth	Date of dissection	Age at death
Elder model 1	06/08/1997	09/10/2020	23
Elder model 2	07/13/1989	09/10/2020	31
Elder model 3	08/03/1995	09/10/2020	25
Elder model 4	07/23/1998	09/11/2020	22
Elder control 1	05/07/1995	09/12/2020	25
Elder control 2	05/03/1994	09/12/2020	26
Elder control 3	08/03/1994	09/12/2020	26

### FVB-hIAPP transgenic mouse islet experiments

#### Mice

Transgenic mice overexpressing human IAPP, FVB/N-Tg (Ins2-IAPP) RHFSoel/J (JAX stock #008232), were used at 12–14 weeks of age (male and female, from Jackson Labs). Age-matched FVB mice were utilized as the control group throughout the study. All animals were housed under specific pathogen-free conditions with a standardized light/dark cycle (12-hour/12-hour) and provided with food and water ad libitum. The use of this isogenic, inbred FVB strain as a control ensures a consistent genetic background against which experimental phenotypes can be accurately compared and quantified. All animal procedures were reviewed and approved by the Institutional Animal Care and Use Committee (IACUC) of ILAS (Protocol Approval Number: QC24004) and were conducted in strict accordance with the relevant guidelines and regulations.

#### Islet isolation

For islet culture experiments, islets were collected using the collagenase method (Collagenase P, Roche, Basel, Switzerland) and cultured overnight. Briefly, 3 ml of 1 mg/ml collagenase P was injected into the opening of the pancreatic duct using a 27 G needle. The pancreas was then removed and placed in a 50 ml tube containing 2 ml of collagenase solution. The tube was later placed in a 37;°C water bath for 12–13 minutes with 100–120 shakings/min until the tissues were homogenously digested. Then, 20 ml of cold HBSS was added to stop the digestion, and the tubes were centrifuged at 300 × *g* for 30 seconds at 4 °C. The islets were then centrifuged using a Ficoll gradient Histopaque-1077 (Sigma‒Aldrich, Kawasaki, Japan) per the manufacturer’s instructions. Finally, the islets were handpicked and incubated overnight in RPMI-1640 medium containing 10% fetal bovine serum, 100 U/ml penicillin, 100 µg/ml streptomycin, 10 mM HEPES, 2 mM L-glutamine, 1 mM sodium pyruvate, 0.05 mM 2-mercaptoethanol and 5.5 mM glucose.

### Method details

#### Virus

The SARS-CoV-2 virus, labeled SARS-CoV-2/WH-09/human/2020/CHN (GenBank: MT093631.2), was supplied by ILAS, PUMC, China. The viral stocks were identified by amplifying the plaque-purified viral isolate as previously described.^10,14^

#### Cells for virus cultivation

SARS-CoV-2 stocks were reproduced using Vero cells. The maintenance of Vero cells was performed using Dulbecco’s modified Eagle’s medium (Invitrogen, Carlsbad, CA, USA) enriched with 10% fetal bovine serum, 100 IU/mL penicillin, and 100 µg/mL streptomycin and incubated at 37 °C and 5% CO_2_. SARS-CoV-2 titers were determined using a 50% tissue-culture infectious dose (TCID_50_) assay.^10,14,46^

#### Viral protein exposure

Islets (~50 per condition in triplicate) were either infected with lentiviral vectors encoding Spike (Spike protein, CMV enhancer-MCS-EF1a-ZsGreen1-T2A-puromycin) or N (N-CTD protein, CMV-MCS-EF1a-mCherry-T2A-neomycin) or treated with 10 µg/mL recombinant Spike S1 or N-CTD protein mixed with 1 µM protein delivery reagent (JiKai Gene) to facilitate uptake. Control islets received control lentivirus or protein delivery reagent without cargo. After 48 h, islets were processed for assays.

#### IHC optimization

Using the Leica BOND RXm stainer, an automated system from Leica Biosystems, IHC optimization was carried out with antibodies that were already optimized and validated,^14^ as noted in the KEY RESOURCES TABLE. Tissue sections of 3–4 μm thickness, fixed in formalin and embedded in paraffin, were baked and dewaxed using Dewax Solution from Leica. Antigen retrieval and antibody stripping were conducted at 100 °C using either Epitope Retrieval Solution 1 or 2, based on the antibody datasheet, along with Bond Wash Solution (Leica). A Novocastra BOND Polymer Refine Detection Kit was used to detect all cell markers, with antibody labeling identified through a diaminobenzidine reaction and counterstaining performed with hematoxylin. To achieve consistent staining, a series of tests were performed using various antibody dilutions and antigen retrieval conditions until the optimal conditions for the primary and secondary antibodies in the positive controls were identified.^14^

#### Multiplex immunofluorescence staining combined with confocal microscopy

We evaluated and confirmed various commercial antibodies to determine if the positive signals were specific reactions. Multiplex immunofluorescence staining was conducted using either an Opal Polaris 7-Color manual IHC Kit or an Opal 7-Color automation IHC Kit. Various primary antibodies were used one after the other to identify specific cell markers, followed by a fluorophore from the Opal 7-Color IHC kit, which includes DAPI and Opal Polaris 520, 540, 570, 620, 650, and 690. The kit for the new panels with seven antibodies included the TSA fluorophore Opal Polaris 480 (#FP1500001KT, Akoya Biosciences). Incubation with HRP-linked secondary antibodies was followed by tyramide signal amplification (TSA). Following each TSA cycle, slides underwent microwave treatment. After antigen labeling, nuclei were stained with DAPI (Sigma‒Aldrich, St. Louis, MO) for 10 minutes and then cover-slipped using Prolong Gold Antifade. Using Leica Application Suite X (Leica Microsystems, Wetzlar, Germany), stained slides were scanned to capture fluorescent spectra at 20-nm wavelength intervals ranging from 420 to 720 nm, maintaining consistent exposure times, and the scans were integrated to form a single stacked image. Alternatively, the Akoya Vectra Polaris advanced multispectral imaging system was used to scan them, allowing for the detection and subsequent quantification of several overlapping biomarkers by separating the signals from each other and from autofluorescence. The images were spectrally unmixed with PerkinElmer inForm® software and exported as multi-image TIFF files.^14^ Total target protein quantification was executed utilizing the Akoya Vectra Polaris advanced multispectral imaging system. Stained tissue sections were spectrally unmixed to separate specific fluorophore signals from endogenous tissue autofluorescence using inForm® software. The Trainable Tissue Segmentation module was employed, utilizing DAPI to define cellular compartments, followed by thresholding the specific fluorescent channels. The total target protein levels were derived by calculating the percentage of the specific target protein-positive fluorescent area relative to the total defined islet area across a minimum of 10 randomly selected, high-power fields per experimental group. This algorithm ensures unbiased, high-fidelity protein quantification.

#### Statistical analysis

Statistical analyses were performed using GraphPad Prism 10.0 (GraphPad Software, San Diego, CA, USA). Data are presented as the mean ± SD or mean ± SEM, as indicated in the corresponding figure legends. All statistical tests were two-sided, and *P* < 0.05 was considered statistically significant. Significance is denoted as *P < 0.05, **P < 0.01, and ***P < 0.001; ns not significant.

The experimental unit was defined according to the biological design of each experiment. For NHP studies, n refers to the number of animals: adult control (AC, *n* = 3), SARS-CoV-2-infected adult model (AM, *n* = 3), elder control (EC, *n* = 3), and SARS-CoV-2-infected elder model (EM, *n* = 4). For image-based analyses, multiple sections, islets, microscopic fields, or ultrastructural images acquired from the same animal were considered technical sampling units; values were averaged within each animal, and animal-level means were used for statistical testing. Accordingly, NHP results were interpreted in the context of effect size and consistency across pathological, fluorescence imaging, ultrastructural, and molecular readouts. The numbers of animals, mice, biological replicates, independent experiments, sections, fields, or images analyzed for each experiment are provided in the corresponding figure legends.

For comparisons between two independent groups, unpaired two-tailed Student’s *t* tests were used unless otherwise specified. Welch’s two-sided *t* tests with Holm correction were used for selected normalized cell-based assays involving multiple comparisons, as indicated in the figure legends. For comparisons involving more than two groups or two experimental factors, one-way or two-way ANOVA followed by Tukey’s or Dunnett’s post hoc test was used, as appropriate. For insulin secretion assays, AUC values were calculated over the indicated GSIS or KSIS intervals before group comparisons. Proteomic differential-abundance and pathway-enrichment analyses were evaluated using adjusted *P* values or false-discovery-rate correction where applicable.

## Supplementary information

Supplementary Files

Supplementary Figure 1

Supplementary Figure 2

Supplementary Figure 3

Supplementary Figure 4

Supplementary Figure 5

Supplementary Figure 6

Supplementary Figure 7–1

Supplementary Figure 7–2

Supplementary Figure 7–3

Supplementary Table 3

Supplementary material of raw size distribution and concentration profiles of exosomes

## Data Availability

The mass spectrometry proteomics data have been deposited to the ProteomeXchange Consortium (https://proteomecentral.proteomexchange.org/cgi/GetDataset? ID = PXD079488) via the iProX partner repository^47,48^ (https://www.iprox.cn/page/project.html?id=IPX0017666000) with the dataset identifier PXD079488. All data associated with this study are in the paper or supplementary materials.
