# Supplementary material for: SARS-CoV-2 seeds IAPP aggregation and β-cell failure
Source: Signal Transduct Target Ther. 2026 Oct 9;11:438. doi: 10.1038/s41392-026-02914-y (PMC13649786; doi:10.1038/s41392-026-02914-y)
Supplement: Supplementary file 1 [file 41392_2026_2914_MOESM11_ESM.pdf]

Plaque-Induced Genes (PIGs) cluster	Upregulation in spatial proteomics
H2-D1	ZRANB3
B2M	ZNF551
C4B	WNT4
GFAP	VWA8
SERPINA3N	VTN
APOE	VPS37C
CTSB	UPRT
HEXA	TUBGCP2
AXL	TRMT2A
CTSD	TRIM56
LGALS3BP	TRAPPC5
CD63	TPM2
CTSL	TMEM258
LYZ2	TMEM11
CD63-PS	THOC3
CTSZ	TGM1
NPC2	TCN2
CD9	TAOK1
H2-K1	TAB3
TREM2	SYNPO2
TYROBP	STX18
ARPC1B	STAT2
C4A	STAMBP
CTSA	ST14
CYBA	SRPX
GRN	SPTLC1
HEXB	SPON1
LAPTM5	SORL1
MPEG1	SLIT3
GPX4-PS	SLC44A1
CLQA	SLC25A43
CLU	SIPA1
CTSS	SH3GL2
FCERLG	SGO2
GUSB	SFRP1
IGFBP5	SERPING1
LGMN	SERPINF1
OLFM13	SERPINB12
S100A6	SEC11A
CLQB	SDR39U1
CSFLR	SDCBP
CTSH	S100A4
FCGR3	RUNDC3A
GNS	RPTOR
ITGB5	RHBDD1
LY86	RBP7
PLEK	QSOX1

RP118A
CLQC
CST3
CX3CR1
FCRLS
GPX4
ITM2B
MAN2B1
PRDX6
VSIR

PRDX4
PLXNB2
PLCL2
PLCB4
PLA1A
PGK2
PDK2
PDK1
PCOLCE
PCM1
PADI2
OSBPL8
OGFOD3
OAT
NUDCD1
NOSTRIN
NID2
NELFB
NDUFB11
MYL4
MYCBP2
MTHFR
MT-CO2
MRPS25
MRPS16
MRPL53
MRPL48
MMAA
MIPEP
MFGE8
METTL16
MAMDC2
LRRK1
LRP1
LOC723314
LOC721200
LOC708923
LOC706710
LOC694628
LOC114674464
LOC106996192
LIN7C
LIN7B
LBP
KRT78
KRT77
KRT5
KRT20

KRT2
KRT16
KRT13
KRT10
KRT1
KPRP
JOSD2
ITIH5
IMP3
ILVBL
IGSF8
IGLV4-69
HYCC2
HYCC1
HSPG2
HPN
HIRIP3
HIPK1
HGF
HDDC3
HADH
GPC6
GBP3
GALNT7
FCGR1A
EXOC7
EXOC6
EXOC5
EXOC3L2
EMC8
EFR3B
DSG1
DSC1
DPP3
DOCK2
DHODH
DES
DERL2
DERL1
DCN
CTNNA2
CRTAC1
CRIP1
CPOX
COL6A2
COL6A1
COL15A1
COG8

COASY
CFH
CFB
CFB
CFAP54
CELF1
CDSN
CCDC127
CAPN7
C9
C8G
C8B
C4BPA
C3
C1S
C1QC
ASCC1
ARG1
APOE
APOA4
APCS
ANKH
AMBP
AKR1C8;LOC722496;AKR1C8
AFM
AFG2A
ADAMTSL1
ADAMTS1
ABCB4
A1BG

Downregulation in spatial proteomics
ZNF598
ZNF346
ZNF24
ZNF22
ZNF207
ZMYM3
ZMYM2
ZHX3
ZC2HC1A
ZBTB20
YWHAZ
YWHAQ
YPEL5
YES1
YAP1
XPO5
WWP2
WDR47
WASHC3
WAC
VWA5B1
VPS35
VMP1
VMA21
VKORC1L1
VDAC2
VASN
VAMP8
VAMP7
VAMP4
UXT
USP40
UMAD1
UBQLN4
UBE2M
UBE2J1
UBE2G2
UBE2D1
UBAP1
UBA1
UACA
TXNRD2
TUSC3
TUBGCP3
TTC38
TTC21B
TSPAN6

TSEN34
TSC22D3
TSC22D1
TRIO
TRIM9
TRIM33
TRIM25
TPRKB
TPI1
TPD52L2
TP53I11
TOX4
TNRC6B
TNFSF10
TMIGD1
TMEM63A
TMEM50B
TMEM254
TMEM222
TMEM199
TMEM119
TMEM106B
TMCC3
TIMP2
THBS3
THAP11
TESC
TDP2
TCEAL5
TCEAL1
TCEA3
TBC1D30
TAGLN3
TAGLN2
TAB2
SZRD1
SYNJ2
SYNE3
SUGP1
STX2
STX10
STIM2
STEEP1
ST13
SSBP1
SRP68
SRCAP
SPTAN1

SPEN
SPATS2L
SPATS2
SP2
SP110
SOWAHC
SNX16
SNRPE
SNIP1
SNCG
SMYD3
SMCR8
SMARCA1
SMAD5
SLC7A4
SLC6A17
SLC5A1
SLC43A1
SLC41A3
SLC38A5
SLC38A4
SLC37A4
SLC35A1
SLC29A1
SLC29A1
SLC25A35
SLC25A3
SLC25A10
SLC20A2
SLC12A7
SIDT2
SHARPIN
SFXN4
SEZ6L
SESN1
SEPTIN7
SEPTIN6
SEPTIN11
SELK
SELENOI
SCRN3
SAYSD1
S
RXRB
RUFY3
RUFY2
RTF2
RPN2

RPLP2
RPL39
RPAP3
RPAP2
ROBO1
RNLS
RNF5
RNF2
RNF146
RIOK3
RIOK1
RIN1
RHPN2
RFX5
RFX1
RETNLB
REPIN1
REEP3
RCN2
RBX1
RBMX
RBM5
RBM22
RBM14
RBIS
RBFA
RASAL2
RASA3
RASA1
RAP2A
RAN
RALBP1
RAE1
RAC2
RABGAP1L
RAB5C
RAB5B
RAB5A
RAB3IP
RAB3B
RAB3A
RAB19
PWP2
PVR;LOC714190
PTPRT
PTPN2
PTBP3
PTBP2

PTBP1
PSMD13
PSMA1
PRXL2B
PRR36
PRR15L
PRKCE
PRKAG2
PRKACG
PPT1
PPP2R1B
PPP1R12B
PPP1R11
POU2F1
POP1
POMT1
POLR2J
POLB
POGZ
PNKD
PNISR
PML
PLP2
PLCD4
PIP4P2
PIN1
PIK3R2
PIK3CB
PHAF1
PHACTR1
PFDN4
PEBP1
PDZK1
PDGFRA
PDE4D
PDE1C
PDCD7
PDAP1
PCYT2
PCMTD1
PCIF1
PCBD1
PBX3
PBX2
PARP9
PARP10
PARP1
PARN

PAOX
PALS1
PALD1
PAIP1
PAFAH1B3
PAFAH1B1
OSCP1
OR52L1
NVL
NUP58
NUBPL
NUB1
NT5DC1
NSUN5
NSMCE4A
NRIP2
NR2C2AP
NPNT
NOXA1
NOP9
NME2
NMD3
NLGN4X
NKAP
NIPSNAP3B
NIFK
NFYB
NFXL1
NFAT5
NEK7
NEDD1
NDUFS6
NDRG1
NDFIP1
ND2
NCBP1
NAP1L5
NACAD
NACA
MYO19
MYH10
MTX3
MTMR1
MTIF3
MTCH2
MSL1
MSH3
MRPS17

MRGPRD
MPRIP
MPI
MOGS
MOB3B
MNX1
MIF
MGAT1
MFN1
MEIS1
MED30
MED25
MED24
MEAK7
MCUR1
MCFD2
MAX
MATR3
MATN2
MAT2A
MAST4
MARCKS
MAPT
MAPK1IP1L
MAP4K4
MAOB
MALT1
MAIP1
MACROD1
LSS
LSM14B
LRRC20
LOC719702
LOC716490
LOC716161
LOC709412
LOC704083
LOC698256
LOC696134
LOC106995404
LMTK2
LIPA
LIMS1
LGALSL
LFNG
LETM1
LEO1
LENG1

LANCL3
KRT7
KRI1
KMT2D
KLC1
KIFC3
KIF19
KIAA1143
KIAA0513
KHDRBS3
KDELR3
KDELR1
JPT2
JPT1
JMJD6
JADE2
ITCH
ISY1
IQSEC3
IP6K1
INTS7
INTS4
INSR
IMPA1
ILF2
IL1RAP
IGSF21
IGHV3-49
IDNK
HSPE1
HSPA8
HSBP1
HOOK3
HNRNPU
HNRNPK
HNRNPA2B1
HNRNPA1
HNF1A
HMGA2
HDHD3
HDGFL3
HAT1
HACD2
H2AC6
GZMB
GTF2A1
GSTP1
GSTM5

GSTK1
GRAMD4
GPR180
GPALPP1
GOSR1
GON7
GNAI2
GMEB2
GLRX5
GLRX
GLMP
GIT2
GFER
GATA4
G3BP1
FUBP1
FOXP1
FKBPL
FKBP7
FDX2
FBXW9
FBXL4
FAM98A
FAM241B
FAM20A
FAM168A
FAM162B
FAM114A1
FAM107B
F10
EVI5L
EVA1B
EVA1A
ESD
ERLIN1
EPB41L5
EPB41L3
EPB41L3
EP400
EML4
EMC4
EMC3
EIF4H
EIF4E
EIF3K
EHMT2
EHBP1
EEF1AKMT2

EDN3
EDF1
ECHDC2
E2F4
DYNLL1
DVL1
DUS3L
DPY19L3
DPP8
DNTTIP2
DNAJB7
DMAC2
DIDO1
DHX40
DENND1A
DENND11
DECR2
DDX31
DCDC2
DCDC1
DBR1
DBNL
CYP2J2
CYB561
CXADR
CWC27
CTTN
CTNNB1
CTCF
CTBS
CSRP1
CSNK2B
CSNK1E
CRY1
CRK
CRELD1
CRACD
CPT1C
CPLX1
COX5B
COQ3
COMMD8
COMMD1
CNOT7
CNKSR3
CMTM6
CMPK1
CLIP4

CLIC2
CLEC16A
CLCN6
CITED1
CIAPIN1
CHURC1
CHUK
CHMP6
CEP43
CDKN2B
CDKN1B
CDK7
CDK17
CDK13
CDH13
CDC40
CD99
CD74
CD200
CCNT2
CCNDBP1
CCDC13
CCDC106
CBX7
CBFB
CASP10
CAPZA2
CAMK2B
CALR
CALM1
CADM1
CACTIN
C3H7orf50
C14H11orf58
C14H11orf52
C11H12orf75
BTF3
BRIX1
BLVRB
BLTP1
BLOC1S5
BICD2
BCO1
BCAS3
BCAN
BBS7
BBLN
AVIL

ATP6V0A2
ATP5ME
ATP1B2
ATN1
ATF2
ATAT1
ARX
ARPC5
ARPC1A
ARL5A
ARHGEF11
ARF5
APTX
APOO
APOC2
APEX1
AP5B1
ANXA6
ANKRD46
ANAPC4
AMPH
ALKAL2
ALG12
ALDOA
ALDH1A1
AKT3
AKT1S1
AKAP7
AKAIN1
AIFM2
AGTRAP
AGO3
AGAP3
ADSS2
ADHFE1
ACTR6
ACTR5
ACTB
ACSS2
ACSF3
ACO2
ABTB3
ABL2
ABI1
ABHD14B
ABCD4
ABCA7
XPC

LBH
