# Supplementary material for: SARS-CoV-2 seeds IAPP aggregation and β-cell failure
Source: Signal Transduct Target Ther. 2026 Oct 9;11:438. doi: 10.1038/s41392-026-02914-y (PMC13649786; doi:10.1038/s41392-026-02914-y)

Size & Concentration Report

5 30×

Data File	20250903 5 30× 25.nfa	Population	Total

SN: FNAN30E20071514
Software: V2.0
Sample Pressure: 1.0Kpa

Laser: 5/40 mW 488
SS Decay: 10%
Threshold/sub: 70.2 9.2 1 1/0 0 0 0
Min Width: 0.3 ms

Total Size Infomation

Gating Range 30 - 150 nm

All Events	2858	
Gating Events	2661	
% of all	93.11	
Median	82.2	nm
Mean	87.1	nm
Std Dev.	19.9	nm

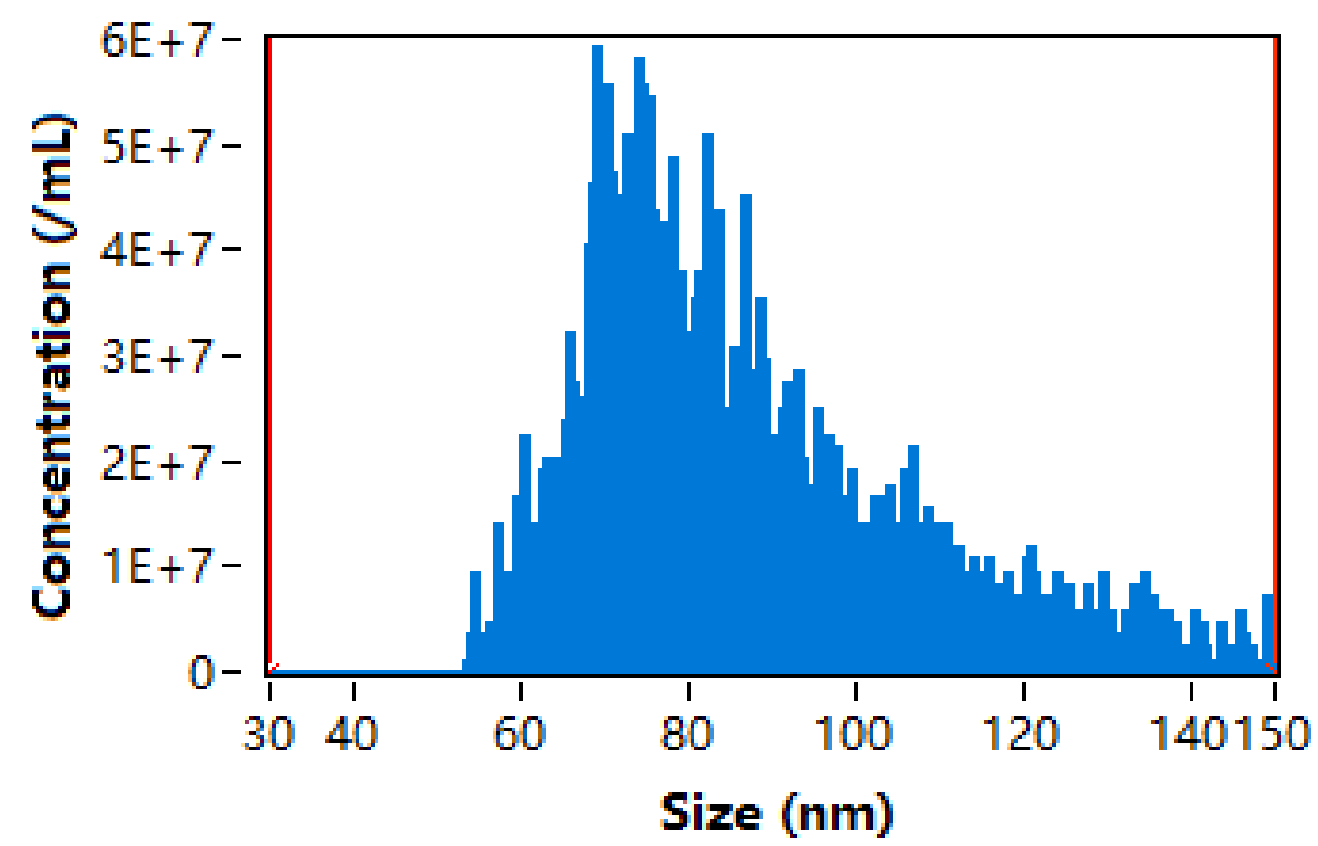

Total Concentration Infomation

	Particle Number	Dilution Factor
STD	5461	100
Blank	51	——
Sample	2909	30

STD Con.	2.16E+10	Particles/mL
Sample Flow Rate	25.28	nL/min
Sample Con.	3.39E+9	Particles/mL

Corrected Ratio: 2858/2858 100.0%

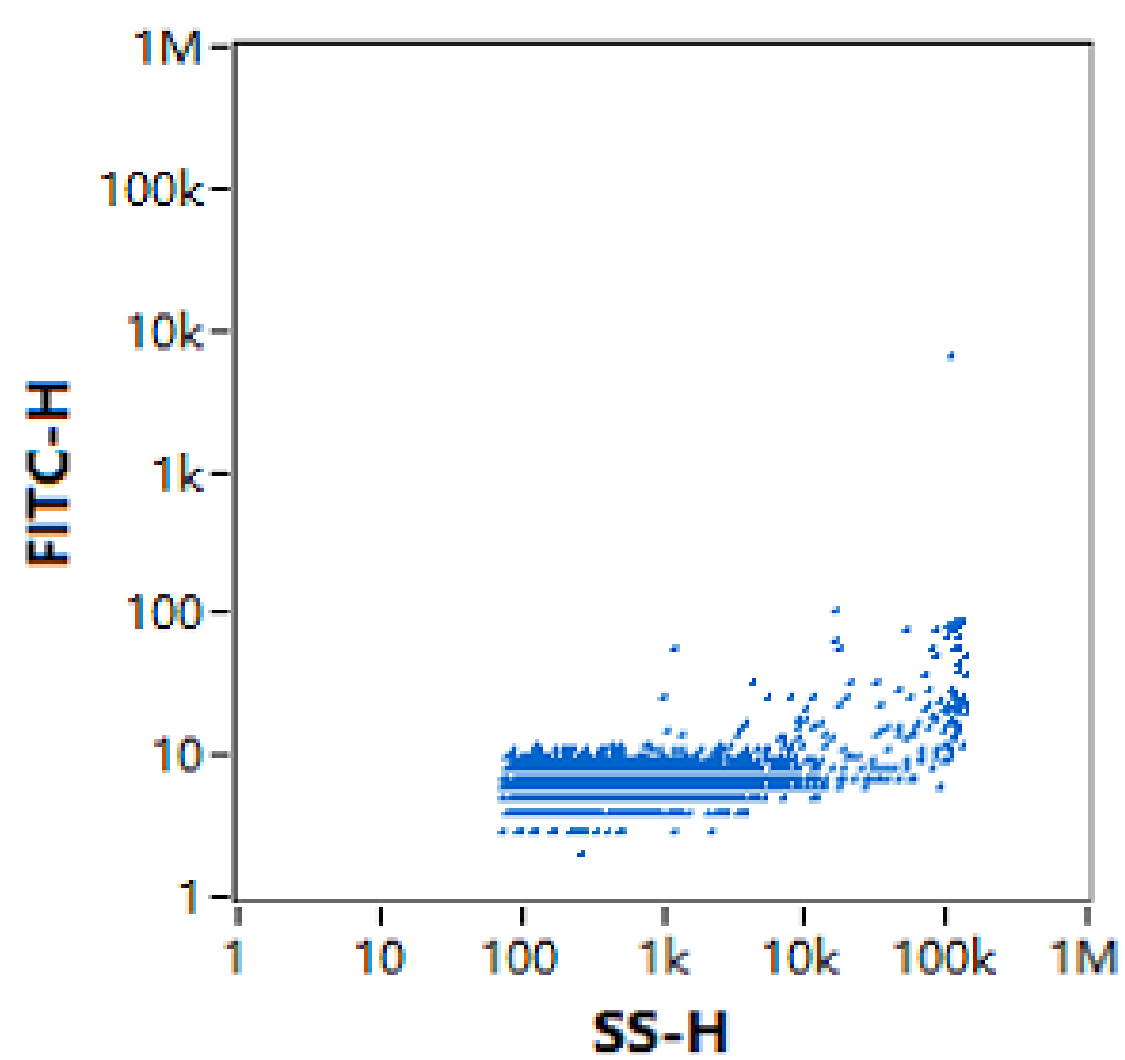

Size & Concentration Report

3 60×

Data File	20250903 3 60× 23.nfa	Population	Total

SN: FNAN30E20071514
Software: V2.0
Sample Pressure: 1.0Kpa

Laser: 5/40 mW 488
SS Decay: 10%
Threshold/sub: 69.8 9 1 1/0 0 0 0
Min Width: 0.3 ms

Total Size Infomation

Gating Range 30 - 150 nm

All Events	2452	
Gating Events	2353	
% of all	95.96	
Median	81.2	nm
Mean	86.5	nm
Std Dev.	18.9	nm

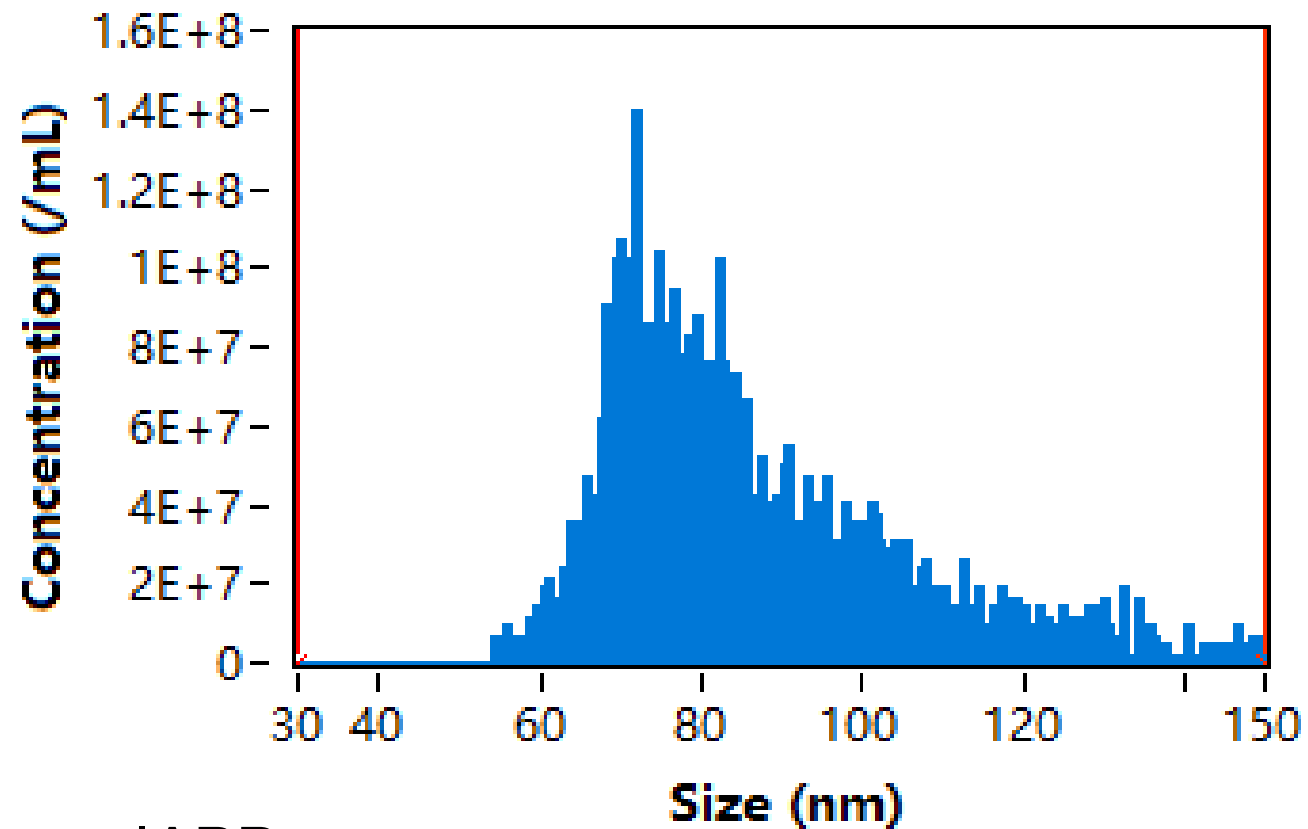

IAPP

Total Concentration Infomation

	Particle Number	Dilution Factor
STD	5461	100
Blank	51	——
Sample	2503	60

STD Con.	2.16E+10	Particles/mL
Sample Flow Rate	25.28	nL/min
Sample Con.	5.82E+9	Particles/mL

Corrected Ratio: 2452/2452 100.0%

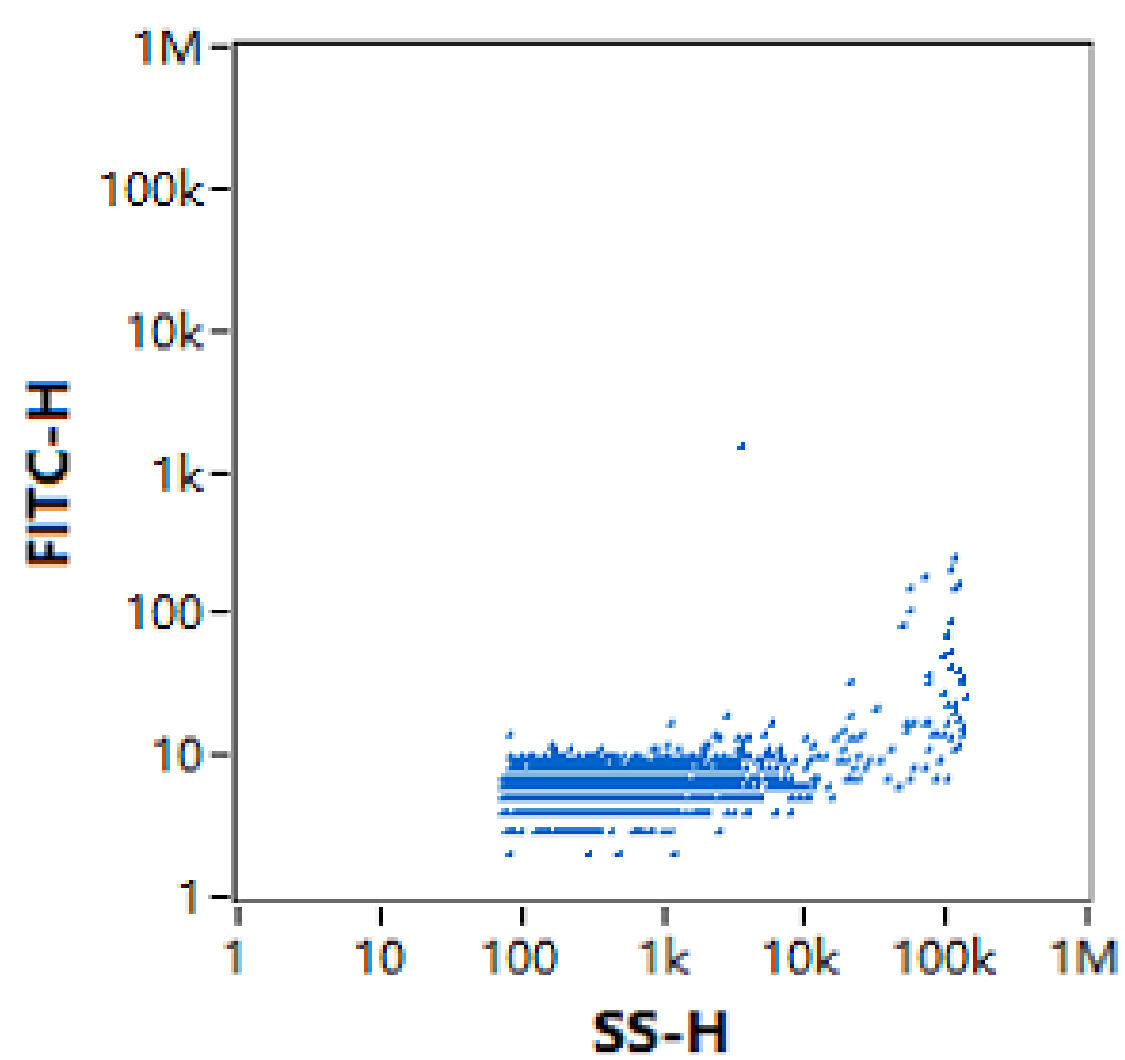

Size & Concentration Report

4 60×

Data File	20250903 4 60× 24.nfa	Population	Total

SN: FNAN30E20071514
Software: V2.0
Sample Pressure: 1.0Kpa

Laser: 5/40 mW 488
SS Decay: 10%
Threshold/sub: 70.3 9.6 1 1/0 0 0 0
Min Width: 0.3 ms

Total Size Infomation

Gating Range 30 - 150 nm

All Events	3303	
Gating Events	3104	
% of all	93.98	
Median	77.8	nm
Mean	81.9	nm
Std Dev.	16.3	nm

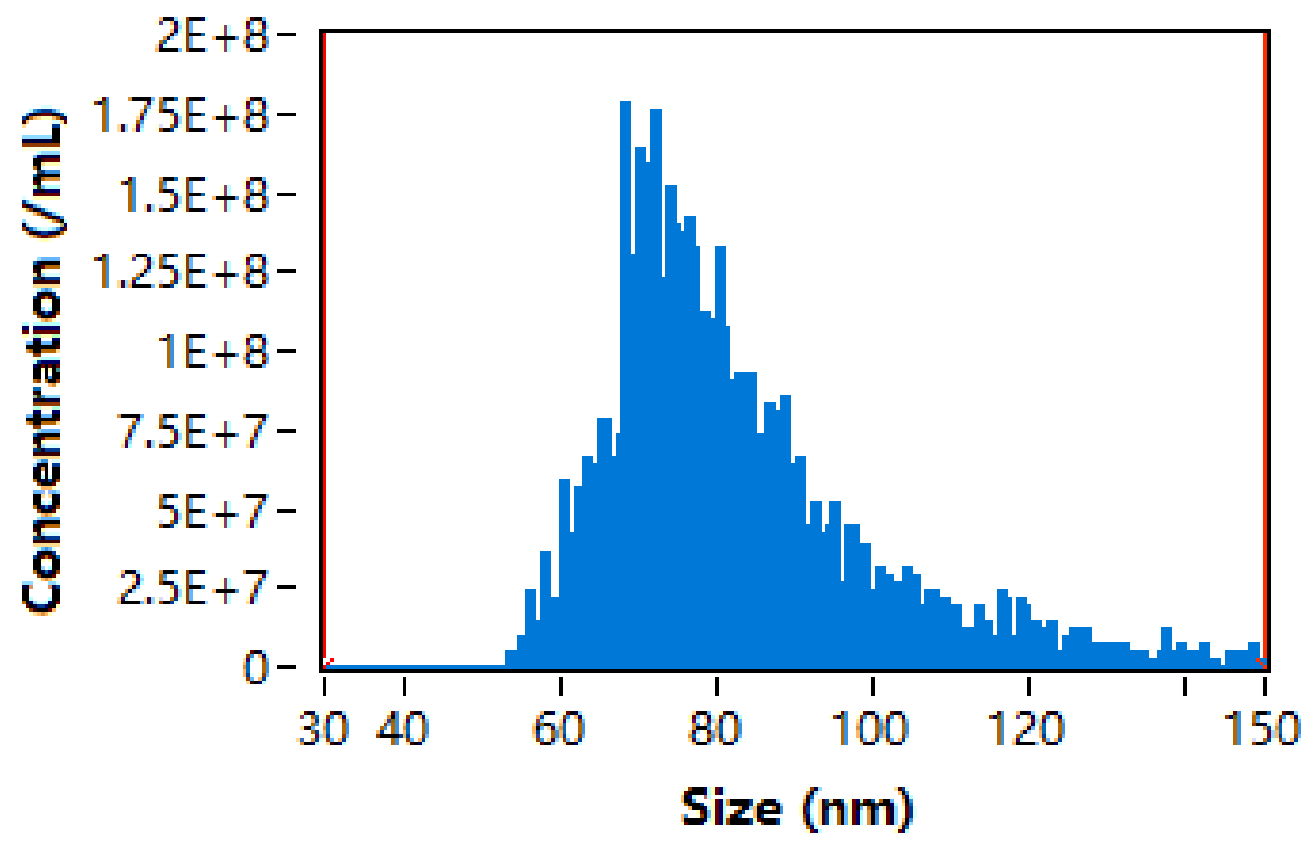

Total Concentration Infomation

	Particle Number	Dilution Factor
STD	5461	100
Blank	51	——
Sample	3354	60

STD Con.	2.16E+10	Particles/mL
Sample Flow Rate	25.28	nL/min
Sample Con.	7.84E+9	Particles/mL

Corrected Ratio: 3303/3303 100.0%

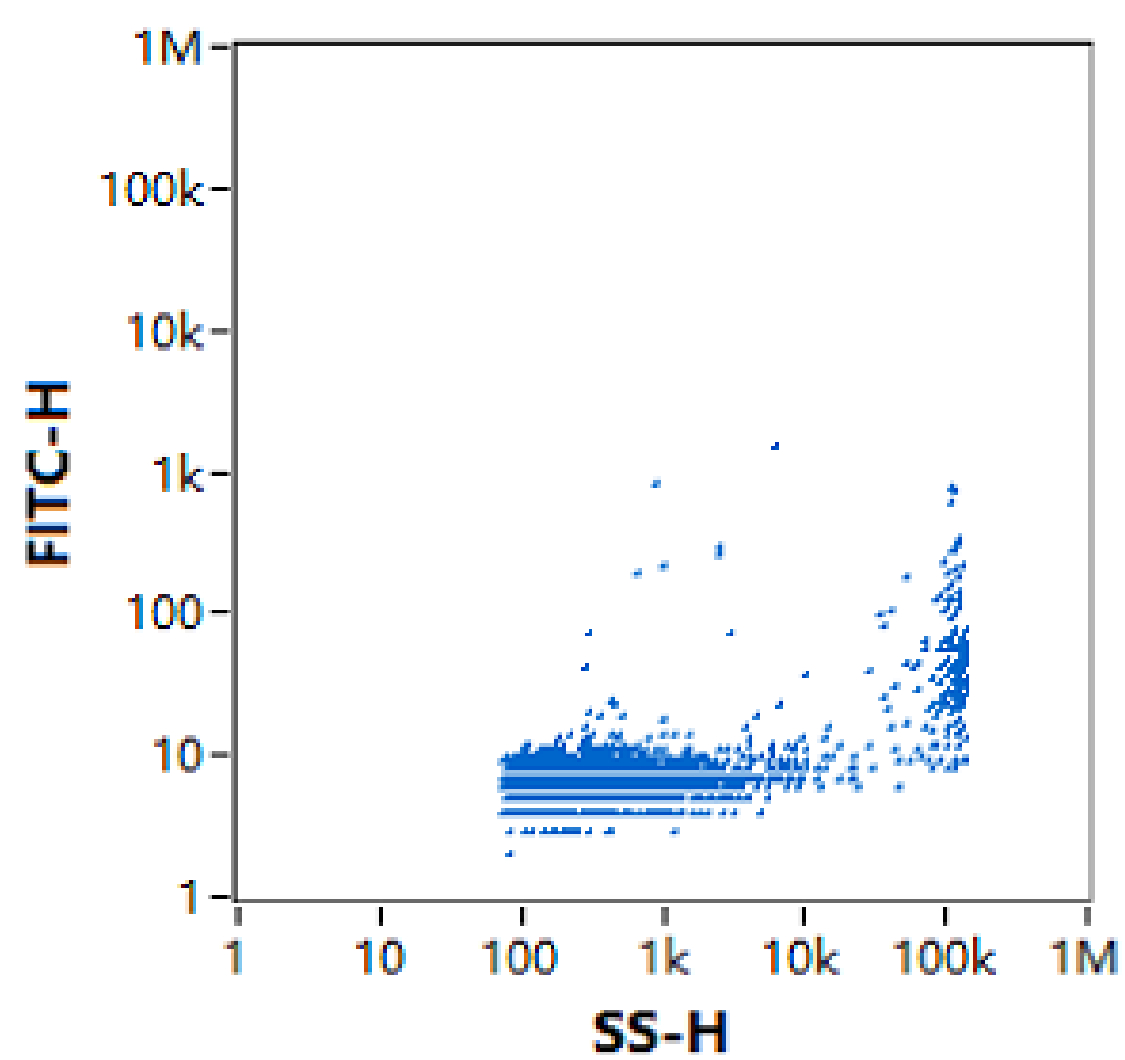

Size & Concentration Report

6 60×

Data File	20250903 6 60× 26.nfa	Population	Total

SN: FNAN30E20071514
Software: V2.0
Sample Pressure: 1.0Kpa

Laser: 5/40 mW 488
SS Decay: 10%
Threshold/sub: 70.9 9.9 1 1/0 0 0 0
Min Width: 0.3 ms

Total Size Infomation

Gating Range 30 - 150 nm

All Events	2952	
Gating Events	2774	
% of all	93.97	
Median	81.2	nm
Mean	86.3	nm
Std Dev.	19.0	nm

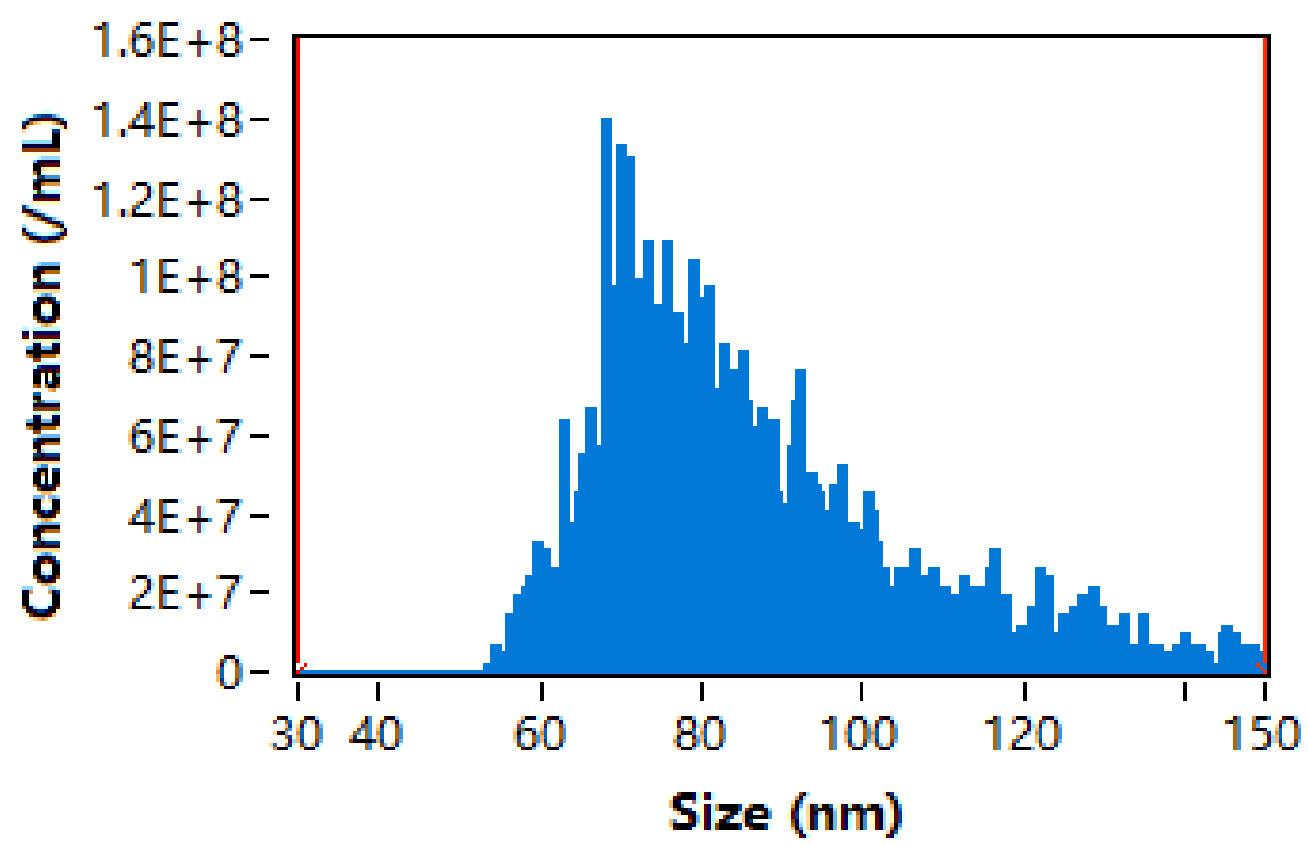

Total Concentration Infomation

	Particle Number	Dilution Factor
STD	5461	100
Blank	51	———
Sample	3003	60

STD Con.	2.16E+10	Particles/mL
Sample Flow Rate	25.28	nL/min
Sample Con.	7.01E+9	Particles/mL

Corrected Ratio: 2952/2952 100.0%

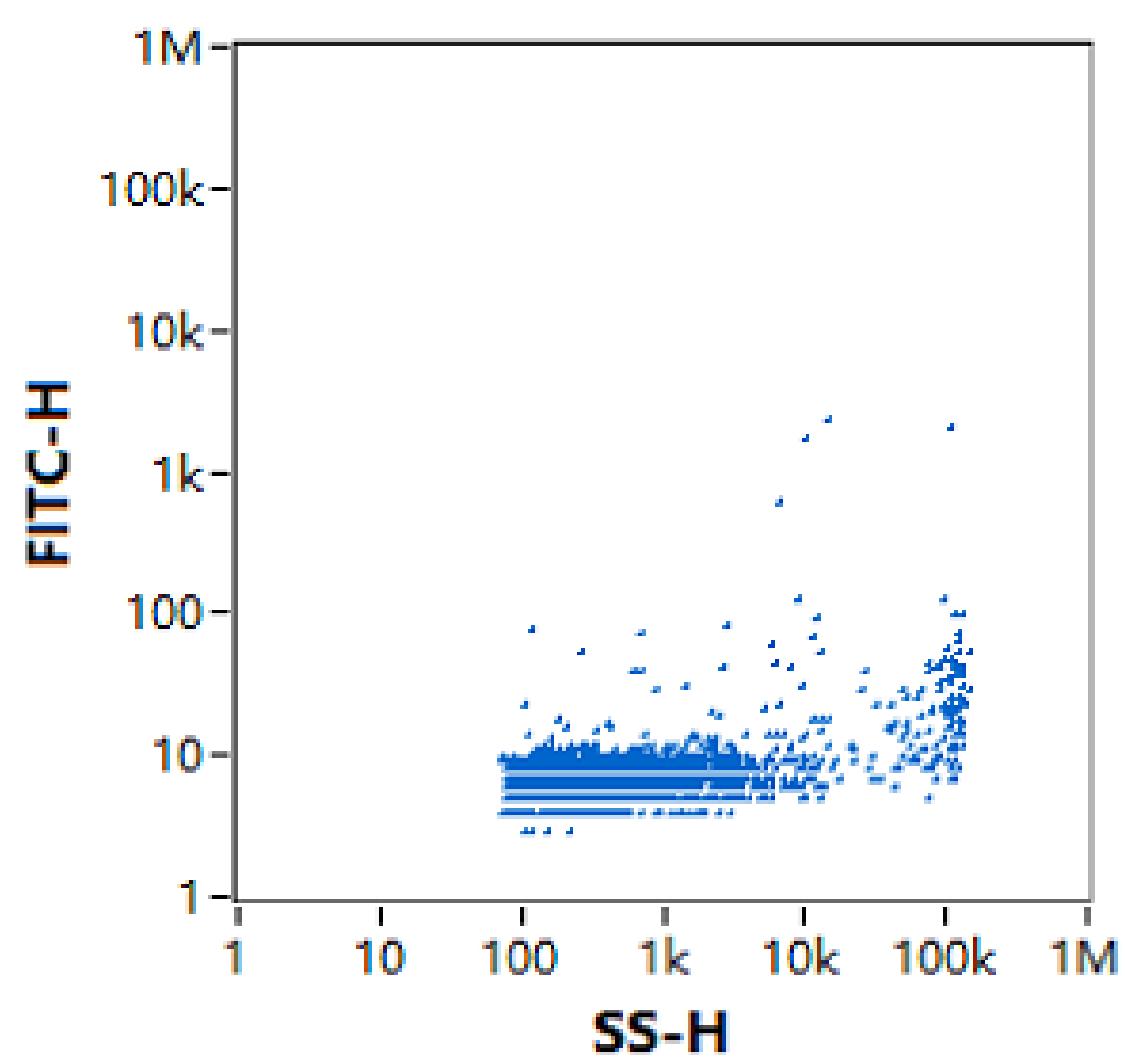

Size & Concentration Report

7 60×

Data File	20250903 7 60× 27.nfa	Population	Total

SN: FNAN30E20071514
Software: V2.0
Sample Pressure: 1.0Kpa

Laser: 5/40 mW 488
SS Decay: 10%
Threshold/sub: 70 9.2 1 1/0 0 0 0
Min Width: 0.3 ms

Total Size Infomation

Gating Range 30 - 150 nm

All Events	3558	
Gating Events	3336	
% of all	93.76	
Median	83.2	nm
Mean	89.3	nm
Std Dev.	21.1	nm

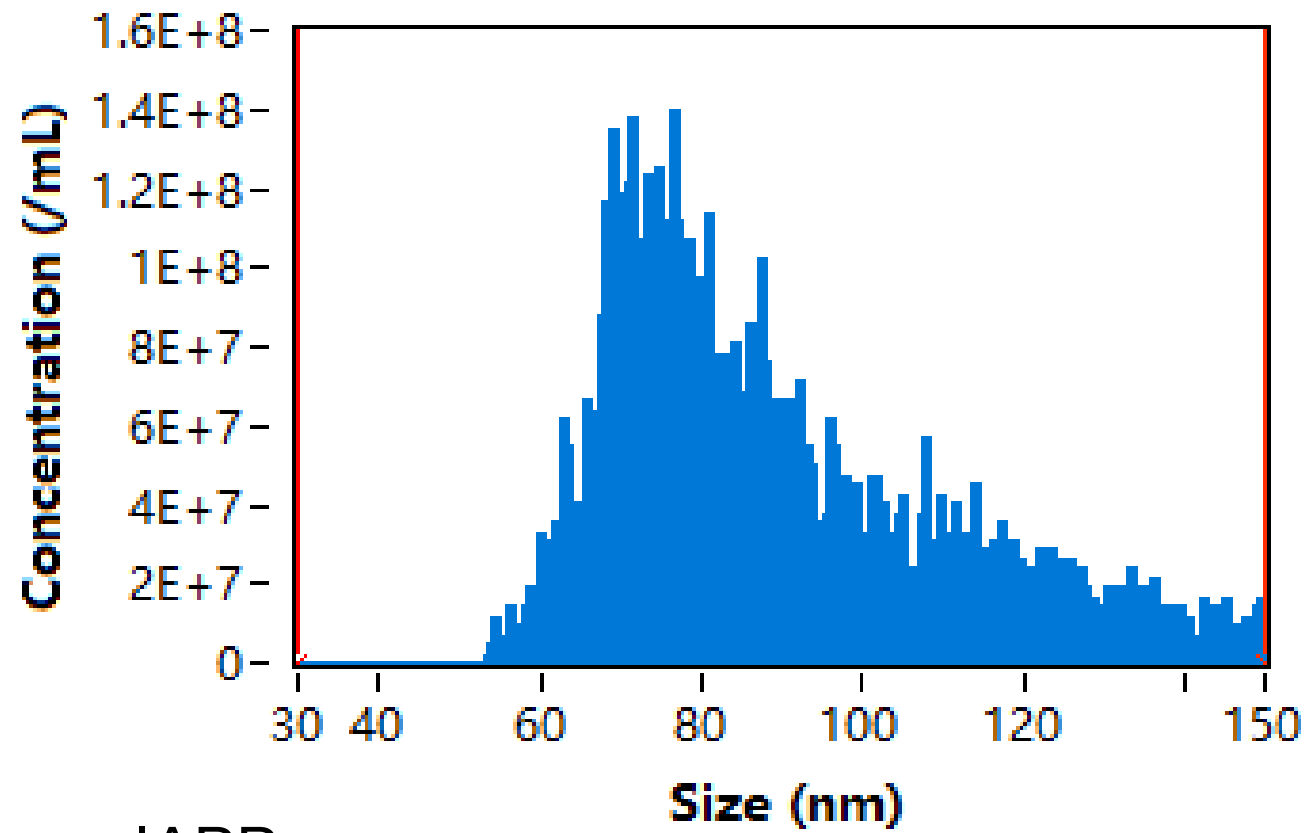

IAPP

Total Concentration Infomation

	Particle Number	Dilution Factor	
STD	5461	100	
Blank	51	———	
Sample	3609	60	
STD Con.	2.16E+10	Particles/mL	
Sample Flow Rate	25.28	nL/min	
Sample Con.	8.44E+9	Particles/mL	

Corrected Ratio: 3558/3558 100.0%

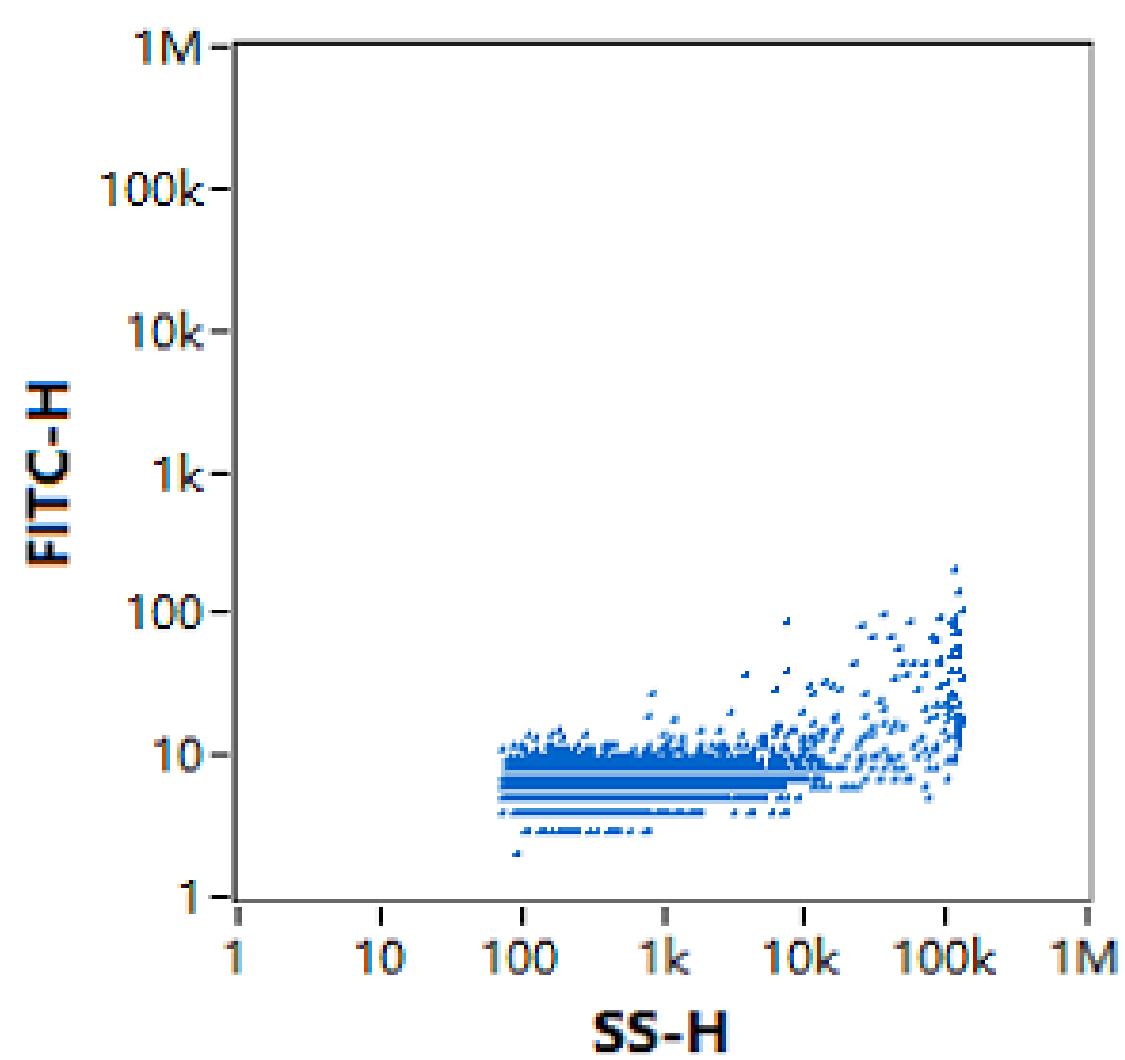

Size & Concentration Report

8 6×

Data File	20250903 8 6× 28.nfa	Population	Total

SN: FNAN30E20071514
Software: V2.0
Sample Pressure: 1.0Kpa

Laser: 5/40 mW 488
SS Decay: 10%
Threshold/sub: 69 11 1 1/0 0 0 0
Min Width: 0.3 ms

Total Size Infomation

Gating Range 30 - 150 nm

All Events	2263	
Gating Events	2104	
% of all	92.97	
Median	83.8	nm
Mean	89.4	nm
Std Dev.	21.3	nm

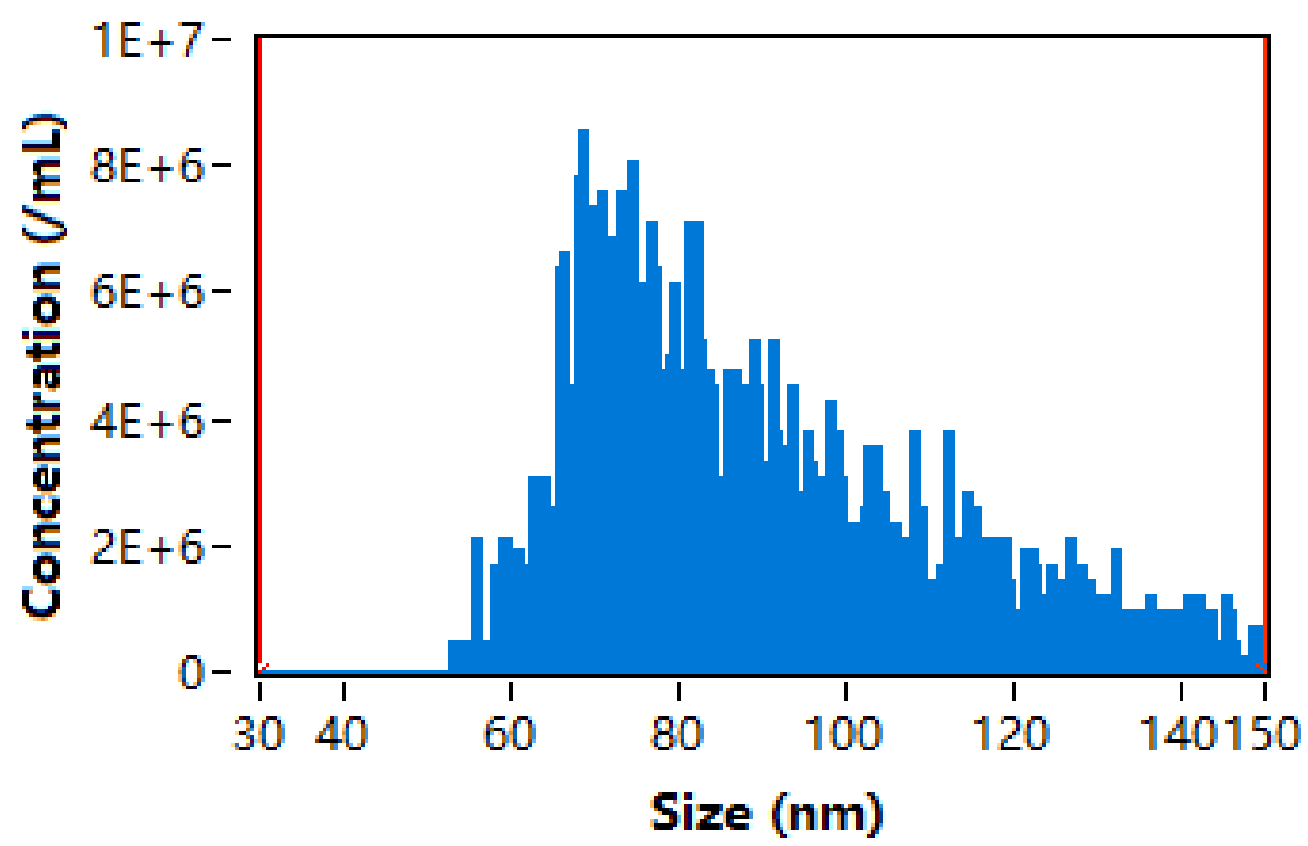

Total Concentration Infomation

	Particle Number	Dilution Factor
STD	5461	100
Blank	51	———
Sample	2314	6

STD Con.	2.16E+10	Particles/mL
Sample Flow Rate	25.28	nL/min
Sample Con.	5.37E+8	Particles/mL

Corrected Ratio: 2263/2263 100.0%

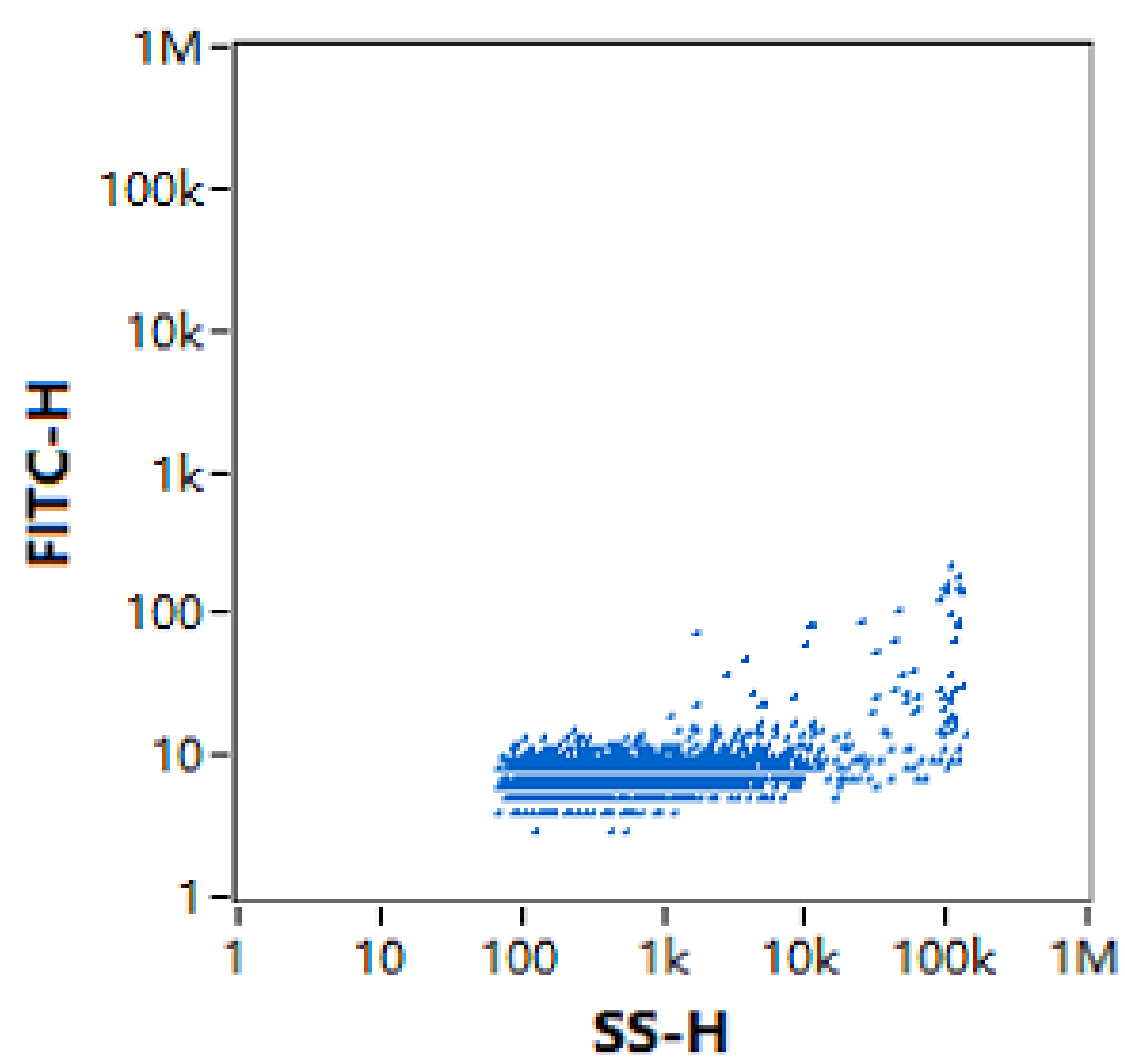

Concentration Report

250 nm Std FL SiNPs

Data File	20250903 250 nm Std FL SiNPs 1.nfa	Population	Total

SN: FNAN30E20071514
Software: V2.0
Sample Pressure: 1.0Kpa

Laser: 20/40 mW 488
SS Decay: 0.2%
Threshold/sub: 67 8 1 1/0 0 0 0
Min Width: 0.3 ms

Total Concentration Infomation

	Particle Number	Dilution Factor
STD	5461	100
Blank	55	——
Sample	5563	100

STD Con.	2.16E+10	Particles/mL
Sample Flow Rate	25.28	nL/min
Sample Con.	2.18E+10	Particles/mL

Corrected Ratio: 5508/5508 100.0%

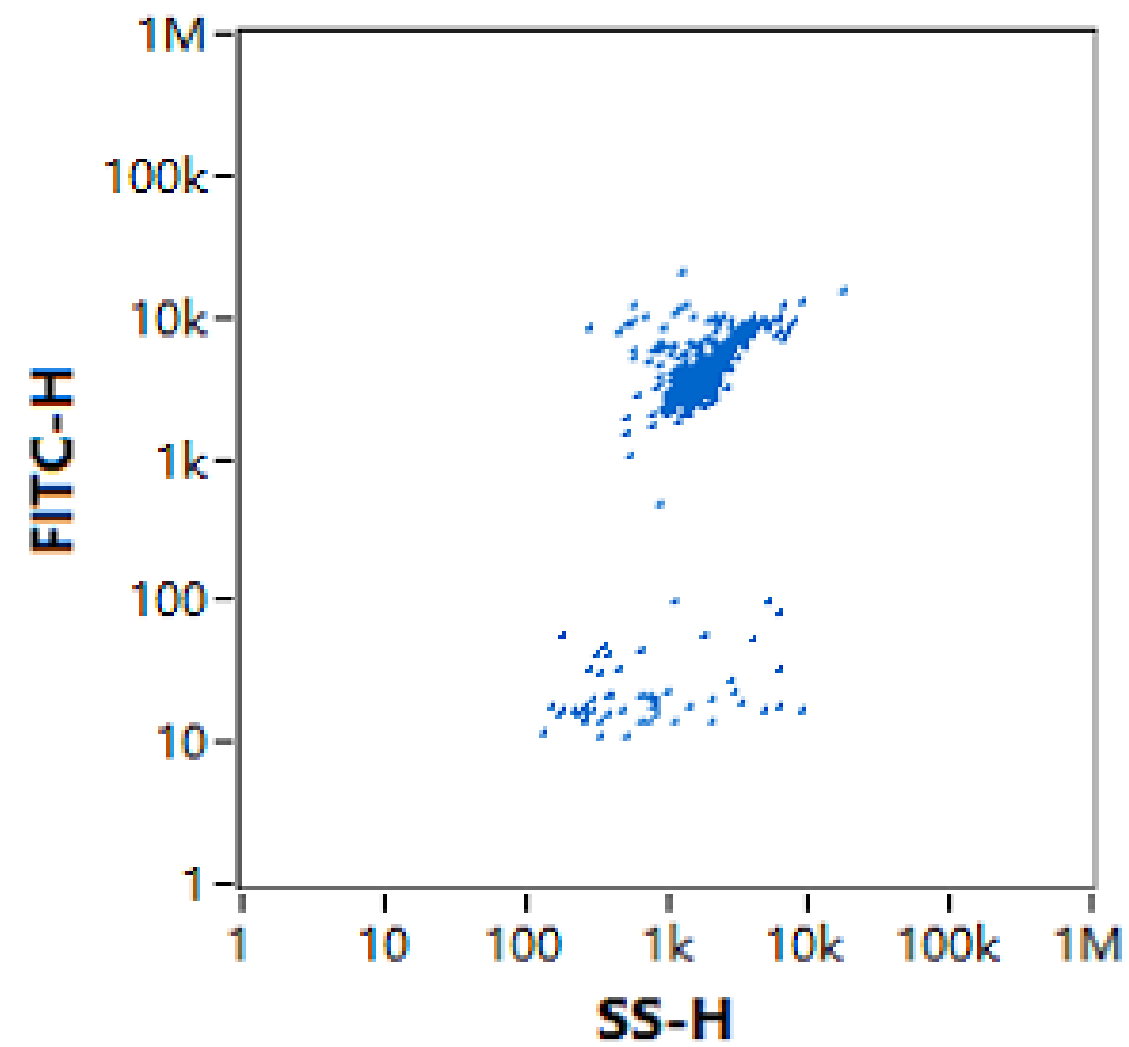

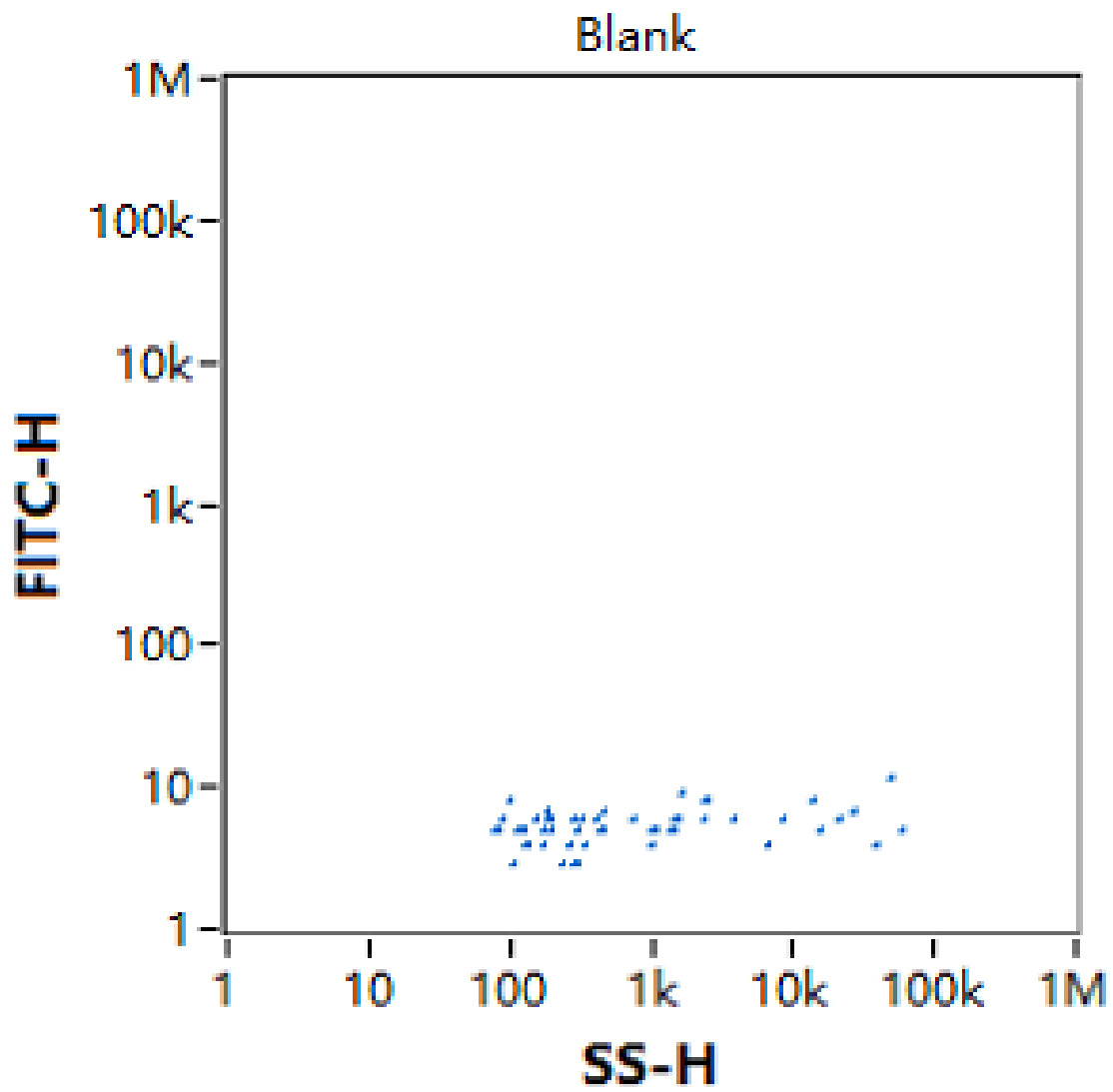

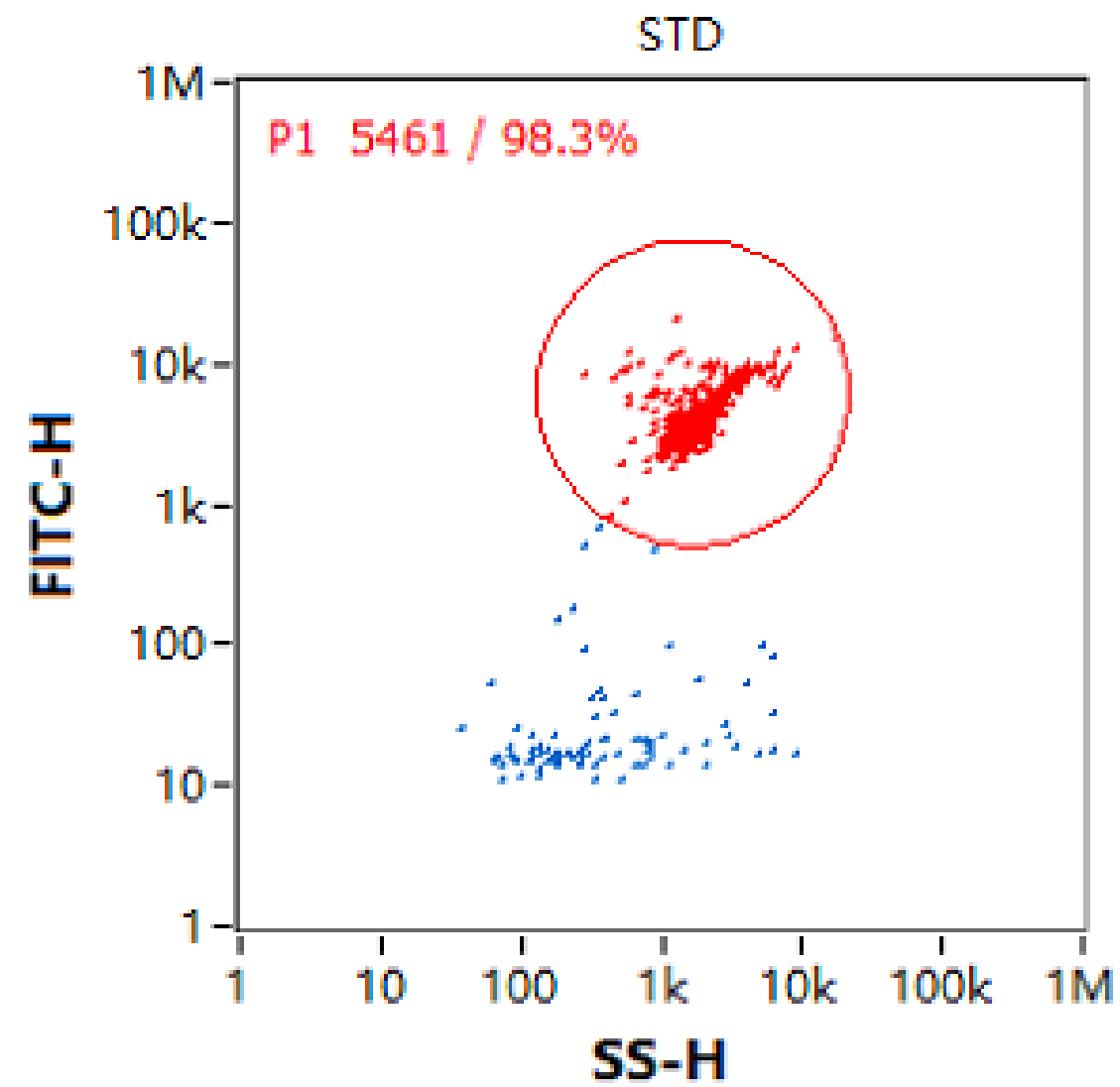

Size Report

68-155 S16M-Exo

Data File	20250903 68-155 S16M-Exo 2.nfa	Population	Total

SN: FNAN30E20071514
Software: V2.0
Sample Pressure: 1.0Kpa

Laser: 5/40 mW 488
SS Decay: 10%
Threshold/sub: 67 8 1 1/0 0 0 0
Min Width: 0.3 ms

Total Size Infomation

Gating Range 30 - 150 nm

All Events	10045	
Gating Events	7648	
% of all	76.14	
Median	89.8	nm
Mean	96.3	nm
Std Dev.	23.0	nm

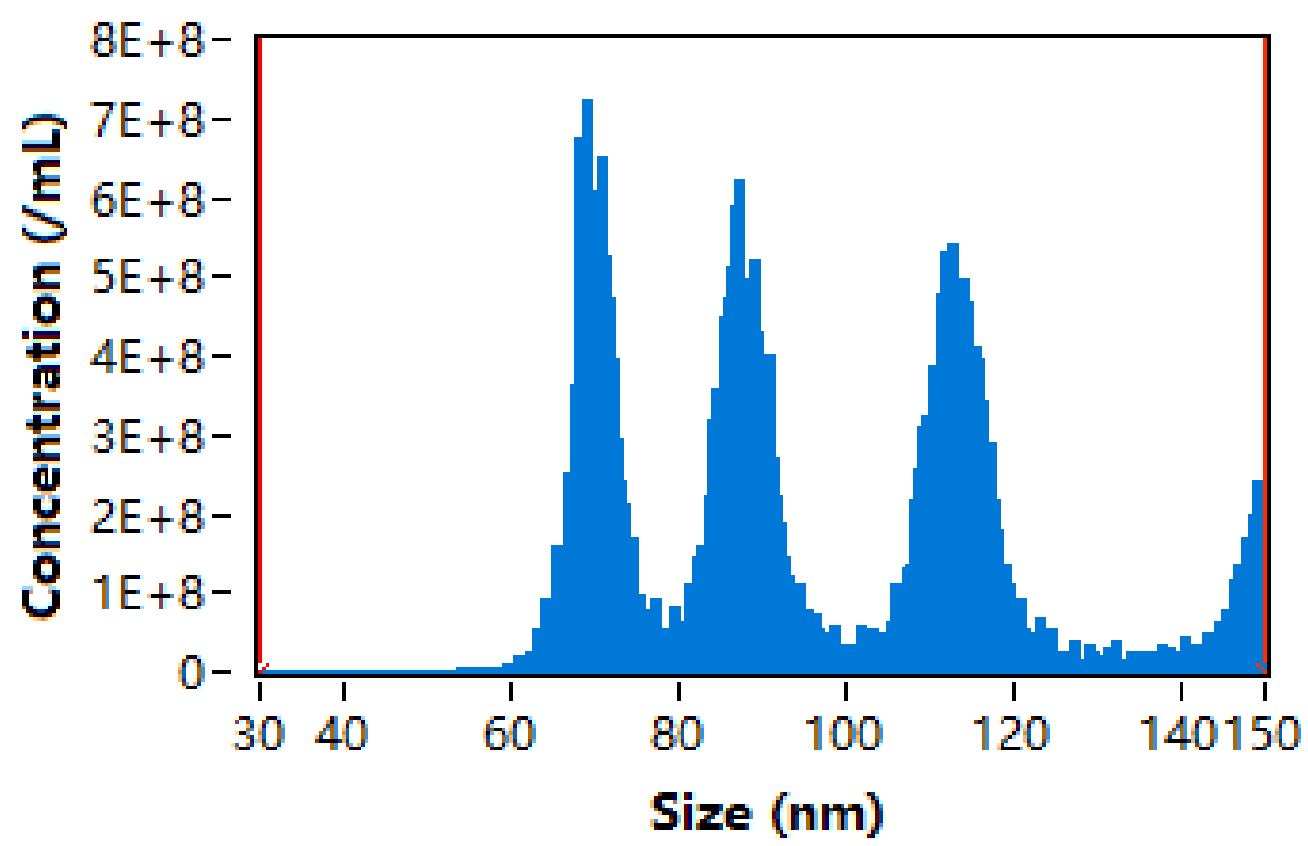

Size (nm)	SS-H
68	151.8
91	456.9
113	1557.9
155	7123.3
0	0
0	0

Standard Curve

Fit type: Power

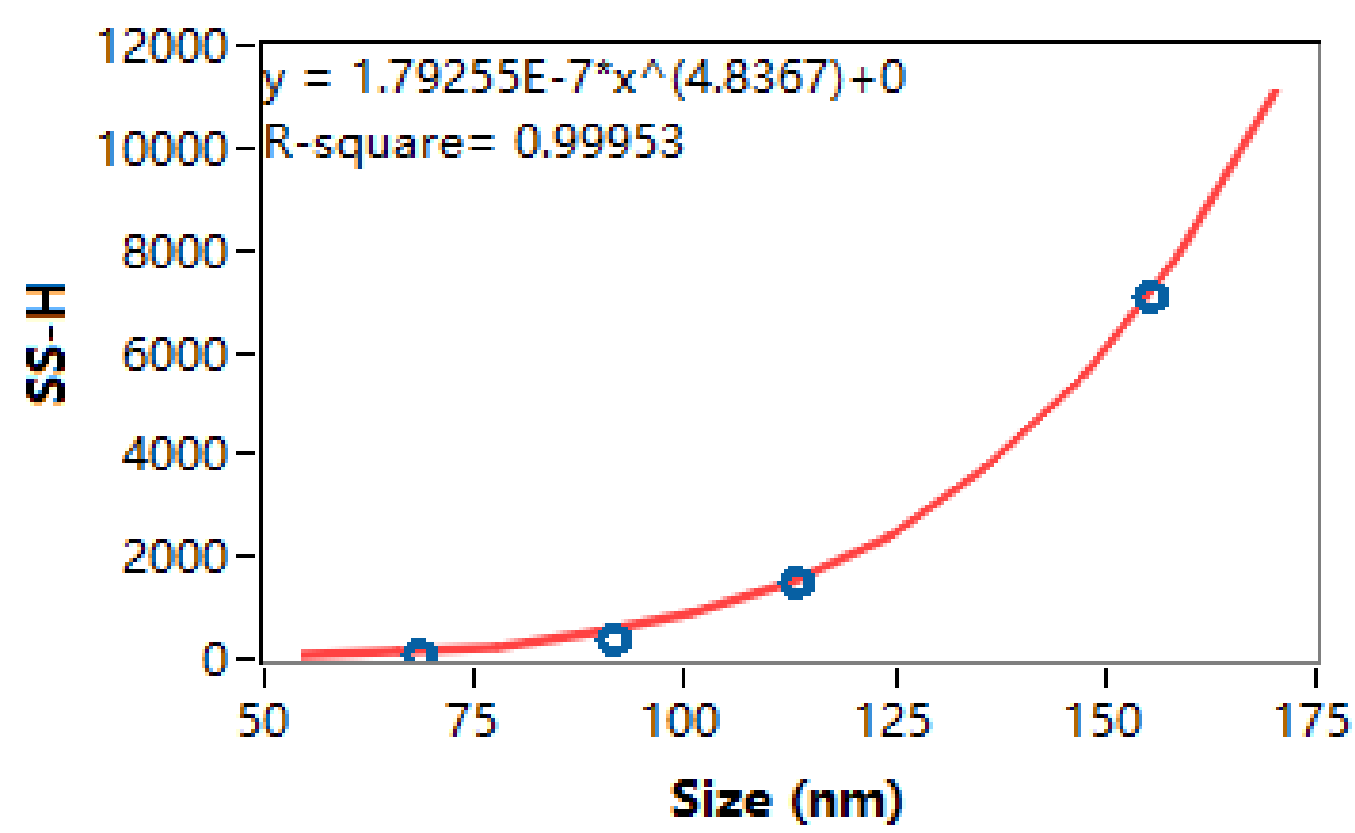

Supplement: Supplementary file 2 [file 41392_2026_2914_MOESM12_ESM.pdf]
