# Supplementary material for: SARS-CoV-2 seeds IAPP aggregation and β-cell failure
Source: Signal Transduct Target Ther. 2026 Oct 9;11:438. doi: 10.1038/s41392-026-02914-y (PMC13649786; doi:10.1038/s41392-026-02914-y)
Supplement: Supplementary file 3 [file 41392_2026_2914_MOESM1_ESM.docx]

Supplementary Materials for

SARS-CoV-2 Seeds IAPP Aggregation and β-Cell Failure

Zhiqi Song^1^, Yanfeng Xu^1^, Wenjie Zhao^1^, Ling Zhang^1,3^, Yanhong Li^1^, Feifei Qi^1^, Wei Liang^1^, Yunlin Han^1^, Ruolin Wang^1^, Qi Lv^1^, Xujian Liang^1^, Fengdi Li^1^, Ran Deng^1^, Wei Deng^1^*, Linlin Bao^1^*, Chuan Qin^1,2^*

^1^ State Key Laboratory of Respiratory Health and Multimorbidity; NHC Key Laboratory of Comparative Medicine; National Human Diseases Animal Model Resource Center, National Center of Technology Innovation for animal model; Beijing Key Laboratory for Animal Models of Emerging and Remerging Infectious Diseases; Key Laboratory of Pathogen Infection Prevention and Control, Ministry of Education, National Institute of Pathogen Biology; Institute of Laboratory Animal Science, Chinese Academy of Medical Sciences and Comparative Medicine Center, Peking Union Medical College, Beijing, China

^2^ Plastic Surgery Hospital, Chinese Academy of Medical Sciences and Peking Union Medical College, Beijing, China

^3^ Institute of Aging and Health, Chinese Academy of Medical Sciences and Peking Union Medical College, Beijing, China

Correspondence:

Wei Deng (dengwei717@163.com), Linlin Bao (baoll@cnilas.org), Chuan Qin (qinchuan@pumc.edu.cn)

**This PDF file includes:**

**Materials and Methods**

Key Resources Table

Protein Structural Modeling and Docking

Surface Plasmon Resonance (SPR) Binding Assays

Cell Culture and Transfection

Co-immunoprecipitation and Western Blotting

Hematoxylin and eosin staining

Histopathological analysis and immunohistochemical (IHC) techniques

Multiplex Fluorescence In Situ Hybridization (FISH) and Co-localization

Absolute Ultrastructural Quantification via Electron Microscopy

RNA Extraction and Quantitative Real-Time PCR (qPCR)

Preparation of IAPP peptides

Aggregation assay and kinetic analysis

Transmission electron microscopy of fibril

Mass Spectrometry of IP samples

MIN6 Functional Assays

Exosome isolation and analysis

Proximity ligation assay (PLA)

Insulin secretion from islets

Cytokine secretion

Proteomic analysis of islets

Transmission Electron Microscopy

Laser-capture microdissection (LCM) and spatial proteomics Workflow

Supplementary Fig. 1-7

Supplementary Tables 1-3

Supplementary material of raw size distribution and concentration profiles of exosomes

**Materials and Methods**

Key Resources Table

| REAGENT or RESOURCE | SOURCE | IDENTIFIER |
| --- | --- | --- |
| Antibodies |  |  |
| Anti-SARS spike glycoprotein antibody [1A9] | Abcam | Cat# ab273433 |
| GeneTex:SARS-CoV-2 (COVID-19) nucleocapsid antibody [HL5511] | GeneTex | Cat# GTX635689 |
| SARS-CoV-2 (COVID-19) nucleocapsid antibody [HL455-MS] | GeneTex | Cat# GTX635712 |
| SARS-CoV-2 (COVID-19) Spike S1 antibody [HL6] | GeneTex | Cat# GTX635654 |
| SARS-CoV-2 (COVID-19) Spike antibody [1A9] | GeneTex | Cat# GTX632604 |
| Anti-Amylin/DAP antibody [EPR22556-138] | Abcam | Cat# ab254259 |
| Anti-Amylin/DAP antibody | Abcam | Cat# ab55411 |
| Anti-Oligomer A11 Polyclonal Antibody | Invitrogen | Cat# AHB0052 |
| Anti-IAPP Fibril Antibody [mOC87] | Abcam | Cat# ab201062 |
| Anti-Insulin antibody [EPR17359] | Abcam | Cat# ab181547 |
| Glucagon Antibody | Cell Signaling Technology | Cat# 2760 |
| Anti-Somatostatin 28 antibody [EPR3359(2)] | Abcam | Cat# ab111912 |
| Anti-Pancreatic Polypeptide antibody [EPR23320-10] | Abcam | Cat# ab272732 |
| Anti-C4BPA antibody [EPR14780] | Abcam | Cat# ab200345 |
| Anti-Hemopexin antibody [EPR5610] | Abcam | Cat# ab226099 |
| Anti-Ceruloplasmin antibody | Abcam | Cat# ab48614 |
| Anti-DDIT3 antibody [9C8] | Abcam | Cat# ab11419 |
| Anti-GRP78 BiP antibody | Abcam | Cat# ab21685 |
| Anti-IL-6 antibody | Abcam | Cat# ab6672 |
| Anti-TNF alpha antibody [TNF706 + P/T2] | Abcam | Cat# ab212899 |
| Anti-VAMP4 antibody [EPR25066-77] | Abcam | Cat# ab290726 |
| Anti-VAMP8/EDB antibody [EP2629Y] | Abcam | Cat# ab76021 |
| Anti-VPS35 antibody [EPR11501(B)] | Abcam | Cat# ab157220 |
| Anti-Rab5 antibody [EPR21801] | Abcam | Cat# ab218624 |
| Anti-SYBL1/VAMP-7 antibody | Abcam | Cat# ab224535 |
| Anti-MLKL (phospho S358) antibody [EPR9514] | Abcam | Cat# ab187091 |
| Anti-RIP3 (phospho S227) antibody [EPR9627] | Abcam | Cat# ab209384 |
| Anti-Rabbit IgG antibody [SP137] | Abcam | Cat# ab208334 |
| Anti-PDX1 antibody [D59H3] | Cell Signaling Technology | Cat# 5679 |
| Anti-Syntaxin 2 antibody | Proteintech | Cat# 55033-1-AP |
| Anti-nkx6.1 antibody [EPR20405] | Abcam | Cat# ab221549 |
| Anti-VAMP2 antibody [D6O1A] | Cell Signaling Technology | Cat# 13508 |
| Anti-STX1A [D4E2W] | Cell Signaling Technology | Cat# 18572 |
| Anti-Syntaxin 4 antibody [EPR15473] | Abcam | Cat# ab184545 |
| Anti-Sodium Potassium ATPase Antibody | Beyotime | Cat# AF1864 |
| Anti-His Tag Antibody | Beyotime | Cat# AF2876-50μl |
| Critical commercial assays | | |
| Thioflavin S | Sigma‒Aldrich | Cat# 1326-12-1 |
| Bond Polymer Refine Detection | Leica Biosystems Newcastle Ltd | Cat# DS9800-CN |
| Bond Wash Solution 10X Concentrate | Leica Biosystems Newcastle Ltd | Cat# AR9590-CN |
| Bond Dewax Solution | Leica Biosystems Newcastle Ltd | Cat# AR9222-CN |
| Bond Epitope Retrieval Solution 1 | Leica Biosystems Newcastle Ltd | Cat# AR9961-CN |
| Bond Epitope Retrieval Solution 2 | Leica Biosystems Newcastle Ltd | Cat# AR9640-CN |
| Bond Primary Antibody Diluent | Leica Biosystems Newcastle Ltd | Cat# AR9352-CN |
| Bond Research Detection System | Leica Biosystems Newcastle Ltd | Cat# DS9455 |
| Bond Aspirating Probe Cleaning Kit | Leica Biosystems Newcastle Ltd | Cat# CS9100 |
| Bond Open Container 7 mL - 10 Pack | Leica Biosystems Newcastle Ltd | Cat# OP79193 |
| Bond Titration kit - 10 Pack | Leica Biosystems Newcastle Ltd | Cat# OPT9049 |
| Opal Polaris 7-Color Manual IHC Kit | Akoya Biosciences | Cat# NEL861001KT |
| OPAL 7-Color auomation IHC KIT 50 SLIDES | Akoya Biosciences | Cat# NEL821001KT |
| Opal Polaris 480 Reagent Pack | Akoya Biosciences | Cat# FP1500001KT |
| Opal Polaris 780 Reagent Pack | Akoya Biosciences | Cat# FP1501001KT |
| RNAscope Probe-V-nCoV2019-S | Advanced Cell Diagnostics | Cat# 848561 |
| RNAscope Probe-V-nCoV2019-orf1ab-sense-C2 | Advanced Cell Diagnostics | Cat# 859151-C2 |
| RNAscopeTM Multiplex Fluorescent Detection Reagents v2 | Advanced Cell Diagnostics | Cat# 323110 |
| RNAscopeTM H2O2 and Protease Reagents | Advanced Cell Diagnostics | Cat# 322381 |
| 2.5HD DAB detection kit | Advanced Cell Diagnostics | Cat# 322300 |
| Duolink® In Situ Proximity Ligation Assay reagent | Sigma‒Aldrich | DUO92008-30RXN |
| SARS-CoV-2 (2019-nCoV) Spike Gene ORF  cDNA clone expression plasmid | Sino Biological | Cat# VG40589-ACG |
| SARS-CoV-2 (2019-nCoV) Nucleoprotein Gene  ORF cDNA clone expression plasmid | Sino Biological | Cat# VG40588-ACR |
| Human IAPP/Amylin Gene ORF cDNA clone  expression plasmid | Sino Biological | Cat# HG13736-CF |
| Cell Counting Kit-8 | Beyotime | Cat#C0038 |
| LDH Cytotoxicity Assay Kit with WST-8 | Beyotime | Cat#C0018S |
| Mouse Insulin ELISA Kit (Ultrasensitive) | Beyotime | Cat#PI602 |
| Mouse IL-6 ELISA Kit | Beyotime | Cat#PI326 |
| Mouse TNF-α ELISA Kit (High-sensitive) | Beyotime | Cat#C0018S |
| Software and algorithms |  |  |
| ImageJ 1.53c | NIH | https://imagej.nih.gov/ij/ |
| Graphpad Prism 10.0 | GraphPad Software | https://www.graphpad.com |
| HALO v3.0.311.262 | Indica Labs | https://indicalab.com/halo/ |
| PerkinElmer's inForm® advanced image analysis software | PerkinElmer, Inc. | https://www.perkinelmer.com.cn/ |
| Phenochart 1.1.0 | PerkinElmer, Inc. | https://www.perkinelmer.com.cn/ |

Protein Structural Modeling and Docking:

*AlphaFold modeling and Molecular docking*

To elucidate the structural basis of the virus-host interaction, we performed one-to-one complex modeling using AlphaFold3 (AF3) between human proIAPP (UniProt: P10997) and two SARS-CoV-2 viral proteins: the Spike protein (GenBank: QOS45029.1) and the Nucleocapsid C-terminal domain (N-CTD; UniProt: P0DTC9). The modeling aimed to generate high-confidence structural predictions of the interaction interfaces. For each complex, the predicted structures were evaluated using the composite model confidence score (pTM + ipTM), where values exceeding 0.75 were considered indicative of a reliable docking outcome. The pTM (predicted Template Modeling) score assesses the global fold accuracy of the entire protein complex, while the ipTM (interface pTM) score specifically quantifies the local structural precision of the protein-protein interaction interface.

Following AF3 structure prediction, the resulting models were visually inspected and rendered using PyMOL (The PyMOL Molecular Graphics System, Version 2.0 Schrödinger, LLC). Protein-protein interaction interfaces within the proIAPP-viral protein complexes were systematically mapped, and subsequent pocket detection analysis was conducted on the IAPP moiety to identify potential binding sites or functional residues involved in the intermolecular recognition. Because the interface-specific confidence remained modest, AF3 models were used only to nominate candidate residues for experimental testing.

Docking of IAPP to Spike and N was done using HDOCK (an FFT-based protein-protein docking server). We treated IAPP as a flexible ligand and the viral protein domains as receptors. We ran 100 docking simulations with semi-flexible refinement. The top 10 poses by docking score were examined. The pose with the lowest binding energy was selected for each complex. We analyzed interfaces by PyMOL and PDBsum to identify interacting residues and hydrogen bonds/salt bridges. Additionally, we used PDBePISA to calculate interface areas and ΔG estimates for each complex.

*Binding energy calculations*

To refine binding energy estimates, molecular dynamics (MD) simulations were performed for the IAPP-Spike and IAPP-N complexes. Each complex was placed in a solvated box with 0.15 M NaCl and run for 50 ns MD (NPT ensemble) using GROMACS with the AMBER force field. Frames from 40-50 ns were extracted (10 frames) for MM/PBSA free energy calculation using g mmpbsa. This yielded an average binding free energy. Separately, PPA-Pred (Protein-Protein Affinity Predictor) was used with the docked complex structures to get an approximate dissociation constant (K D) at 25°C.

Surface Plasmon Resonance (SPR) Binding Assays

*IAPP or IAPP pentamutant with SARS-CoV-2 Spike or nucleocapsid protein*

The protein-protein interactions were quantitatively analyzed using Surface Plasmon Resonance (SPR) on a Biacore 8K instrument (Cytiva). Recombinant Human Islet Amyloid Polypeptide (IAPP) or IAPP pentamutant (R44A/I59A/S62A/T69A/Y70F) peptides was used as the analyte, while Recombinant SARS-CoV-2 Spike S1+S2 ECD protein and the Recombinant Human coronavirus SARS-CoV-2 nucleocapsid protein served as ligands. Experiments were performed by Shanghai Stawin Biotechnology Co., Ltd. The ligands were immobilized on a CM5 sensor chip (Cytiva) via standard amine-coupling chemistry. Briefly, the carboxymethylated dextran surface was activated with a mixture of N-ethy-N'-(3-dimethylaminopropyl) carbodiimide hydrochloride (EDC) and N-hydroxysuccinimide (NHS). Optimal immobilization conditions for each ligand were determined by pre-concentration tests across a pH gradient (acetate buffers, pH 4.0-5.5). The Spike and Nucleocapsid proteins were subsequently immobilized in pH 5.0 and pH 5.5 acetate buffer, respectively, at a flow rate of 10 µL/min for 360 seconds.

For kinetic measurements, serial dilutions of the IAPP analyte were prepared in HBS-EP running buffer, ranging from 4 µM down to 0.03125 µM. Binding assays were performed at 25 ℃ with a constant flow rate of 30 µL/min. The association phase was monitored for 120 seconds, followed by a 240-second dissociation phase in running buffer. The sensor surface was regenerated with a 30-second pulse of 2.5 mM glycine-HCl (pH 2.5) after each binding cycle. All experiments were performed in multi-cycle kinetics mode.

Sensorgram data were reference-subtracted and fitted to a 1:1 binding model using the Biacore 8K evaluation software. The association rate constant (ka, 1/Ms), dissociation rate constant (kd, 1/s), and equilibrium dissociation constants (binding affinity, KD) of interaction were calculated using BIAcore® 8K evaluation software.

*ITM2B with IAPP or IAPP+SARS-CoV-2 Spike or IAPP+SARS-CoV-2 nucleocapsid protein*

ITM2B and IAPP or IAPP + Spike or IAPP + necleocapsid mixture protein binding kinetics were performed on the same Biacore 8K platform. A running buffer containing HBS-EP was prepared, filtered and degassed before use. Flow cells of a CM5 sensor chip were activated with a 1:1mixture of 0.1 M N hydroxysuccinimide and 0.1 M N-ethyl-N’-(3- diethylaminopropyl)-carbodiimide at a flow rate of 10 μL/min. The ITM2B or IAPP + Spike or IAPP + necleocapsid mixture protein were diluted in 10 mM sodium acetate (pH 5.0) to a concentration of 5 μg/mL, and immobilized on the CM5 chip to a response signal. The remaining binding sites on the chip were blocked by 1 M ethanolamine (pH 8.5). Indicated Islet amyloid at a series of concentrations were injected into the flow cell to pass over the immobilized target. The binding signal was measured by a multiple-cycle method with 120 s association and 240 s dissociation at a flow rate of 30 μL/min. Sensor surfaces were regenerated with glycine-HCl (pH 2.0). The equilibrium dissociation constants (binding affinity, KD) of interaction were calculated using BIAcore® 8K evaluation software.

Cell Culture and Transfection:

*Cell lines*

PANC-1 (human pancreatic ductal cell line), HEK293T (human embryonic kidney) were obtained from ATCC and maintained in DMEM with 10% fetal bovine serum and penicillin-streptomycin at 37°C, 5% CO_2._ MIN6 (mouse insulinoma) cells were maintained in DMEM with 15% FBS and β-mercaptoethanol (50 µM). All cell lines were Mycoplasma-free (tested by PCR).

*Plasmids*

a. Human IAPP/Amylin Gene ORF cDNA clone expression plasmid, C-Flag tag; b. SARS-CoV-2 (2019-nCoV) Spike Gene ORF cDNA clone expression plasmid, C-GFPSpark tag (vector was pCMV3-C-GFPSpark); c. SARS-CoV-2 (2019-nCoV) Nucleoprotein Gene ORF cDNA clone expression plasmid, COFPSpark tag (vector was pCMV3-C-OFPSpark); d. Control plasmids was pCMV3-C-Flag (empty Flag). All constructs were verified by sequencing.

*Transfection*

For immunofluorescence and co-IP experiments, PANC-1 or 293T cells were seeded in 6-well plates and transfected at ~70% confluency using Lipofectamine 3000 (ThermoFisher) according to manufacturer instructions. A 1:1 ratio of IAPP plasmid and viral protein plasmid (total 2 µg DNA per well) was used for co-transfections; equal total DNA was kept by adding empty vector to single transfections. Transfection efficiency in 293T was >80%; in PANC-1 it was ~40–50%. For MIN6, which are more difficult to transfect, we used a Nucleofection approach (Lonza kit) to introduce plasmids, or alternatively treated cells with purified proteins (see below). Cells were harvested 48 h post-transfection for analyses.

Co-immunoprecipitation and Western Blotting

Transfected 293T cells were lysed in IP lysis buffer (50 mM Tris pH 7.5, 150 mM NaCl, 1% NP-40, 0.1% SDS, 5 mM EDTA, plus protease inhibitor cocktail). 5% of each lysate was saved as “Input”. The rest was incubated with anti-Flag M2 affinity agarose (Sigma) overnight at 4°C with gentle rotation. Beads were washed 4× with lysis buffer. For negative control IP, a parallel set of lysates was incubated with normal mouse IgG bound to agarose. After washes, bound proteins were eluted by boiling in SDS sample buffer.

For Western blot, proteins were resolved on 4-20% gradient SDS-PAGE gels and transferred to PVDF membranes. Blots were probed with: anti-Flag-HRP (1:5,000) to detect IAPP-Flag, anti-PDX1 (1:1,000), anti-VAMP4 (1:1,000), anti-VAMP8 (1:1,000), anti-VAMP2 (1:1,000), anti-Rab5 (1:1,000), anti-VPS35 (1:1,000), anti-STX2 (1:1,000), anti-SNAP25 (1:1,000), anti-MAFA (1:1,000), anti-NKX6-1 (1:1,000), anti-STX1A (1:1,000), anti-STX4 (1:1,000), anti-Spike S1 (1:2,000) followed by HRP-secondary, anti-Nucleocapsid (1:3,000), and anti-GAPDH (1:5,000) or β-actin (1:5,000) as loading control. Bands were visualized by ECL substrate on a ChemiDoc imager (Bio-Rad). Co-immunoprecipitated Spike and N proteins were identified by their expected molecular weights (~180 kDa for Spike monomer, often appearing as smeared band due to glycosylation; ~50 kDa for N).

Additionally, after IP, we ran one gel for Coomassie blue staining to verify pull-down efficiency and protein integrity (Figure S2B). The Coomassie gel of Flag IP samples showed prominent bands corresponding to IAPP-Flag and co-precipitated partners in the co-transfected conditions, and no bands in the IgG controls, confirming the specificity.

Hematoxylin and eosin staining

All harvested organs were preserved in a 10% buffered formalin solution within a wet specimen bank, and paraffin sections measuring 3-4 µm in thickness were prepared following standard procedures. H&E staining was applied to all tissue sections, and an Olympus microscope was used to observe histopathological changes in various tissues.

Histopathological analysis and immunohistochemical (IHC) techniques

The organs collected were preserved in a 10% buffered formalin solution, and paraffin sections with a thickness of 3-4 µm were prepared according to a previous report. We have evaluated and confirmed various commercial antibodies to determine if the positive signal of viral antigens or different antibodies is a specific reaction. Briefly, paraffin sections were created and stained with H&E before being examined under a light microscope. For IHC staining or initial experiments to detect the expression of various antibodies manually, dehydrated paraffin sections were processed with an antigen retrieval kit (AR0022; Boster Bio, Pleasanton, CA) for 1 minute at 37℃ and quenched for endogenous peroxidases in 3% H_2_O_2_ in methanol for 10 min. Following a 1-hour block in 1% normal goat serum at room temperature, the sections were treated with different antibodies at 4℃ overnight, followed by incubation with a horseradish peroxidase (HRP)-labelled goat anti-mouse IgG secondary antibody (ZDR-5307, 1:200; ZSGB Bio), HRP-labelled goat anti-rabbit IgG secondary antibody (ZDR-5306, 1:200; ZSGB Bio), or HRP-labelled goat-anti rat IgG secondary antibody (ZF-0312, 1:200; ZSGB Bio) for 1 h. Ultimately, the sections were incubated with 3,3′-diaminobenzidine tetrahydrochloride (DAB) and observed under an Olympus microscope. Sequential sections from all gathered tissues were directly treated with HRP-labeled goat anti-mouse or anti-rabbit IgG, serving as the omission control for various antibody staining. Sequential sections from all collected tissues were incubated with a recombinant anti-rabbit IgG antibody [SP137] as the negative control for protein expression.

In situ hybridization

SARS-CoV-2 genomic RNA in formalin-fixed paraffin-embedded tissues was analyzed using ISH with the RNAscope® 2.5HD DAB detection kit and the ISH Probe-V-nCoV2019-S (genomic RNA fragment 21631-23303, RefSeq #NC_045512.2), following established protocols. In summary, tissue sections were deparaffinized using xylene, followed by a series of ethanol washes and peroxidase blocking, then heated in antigen retrieval buffer and digested with proteinase. The sections underwent exposure to an ISH target probe and were incubated at 40°C in a hybridization oven for 2 hours. After rinsing, the ISH signal was amplified using a pre-amplifier and amplifier conjugated to alkaline phosphatase and incubated with DAB for visualization at room temperature. The sections were stained with hematoxylin, allowed to air dry, mounted, and kept at 4°C until they were analyzed.

Multiplex Fluorescence In Situ Hybridization (FISH) and Co-localization

To visualize active viral replication centers in situ, a highly specific “2 RNA + 1 Protein” multiplex assay was developed. Advanced RNAscope technology was utilized with proprietary probes designed to simultaneously recognize the SARS-CoV-2 positive-sense genomic RNA (representing structural virions) and the negative-sense (antisense) RNA (representing the obligate active replication intermediate) according to the manufacturer’s instructions. Following the rigorous hybridization protocol, tissue sections were co-stained with anti-IAPP antibodies. High-resolution confocal microscopy coupled with HALO image analysis software was deployed to perform absolute quantification of the spatial distance and physical colocalization percentages between the active antisense RNA clusters and the dense IAPP amyloid plaque cores. For object-based plaque-association analysis, the antisense RNA channel was used as the primary readout of discrete replication-associated foci, whereas the positive-sense RNA channel was interpreted as a broader genomic RNA signal. An antisense RNA-positive focus was considered plaque-associated when its segmented signal mask intersected the segmented IAPP-positive plaque mask within the same field. The overlap percentage was calculated as the number of plaque-associated antisense foci divided by the total number of antisense foci per field.

Absolute Ultrastructural Quantification via Electron Microscopy

For ultrastructural analysis, small pieces of pancreatic tissue (~1 mm^3^) from monkeys were fixed in 2.5% glutaraldehyde/2% PFA in 0.1 M phosphate buffer (pH 7.4) at 4°C overnight. Samples were post-fixed in 1% osmium tetroxide, en bloc stained with 2% uranyl acetate, dehydrated through graded ethanol, and embedded in Epon resin. Ultrathin sections (70 nm) were cut and mounted on copper grids. Sections were counterstained with uranyl acetate and lead citrate and examined on a JEOL 1400 transmission electron microscope (JEOL, Tokyo, Japan) at 80 kV. Digital images were captured with an AMT CCD camera.

For absolute structural quantification, blinded researchers utilized specialized image analysis algorithms to delineate 100 µm² regions of interest within the β-cell cytoplasm. Manual, rigorous counting was performed to quantify mature insulin granules (identified by a distinct dense crystalline core and clear halo), immature/empty vesicles, and pathological “aggregated-wrapped vesicles” (vesicles completely entombed by fibrillar networks). Mean vesicular diameters were calculated to evaluate accumulation.

RNA Extraction and Quantitative Real-Time PCR (qPCR)

*RNA Extraction*

Total RNA was extracted from cell samples using a silica membrane column‑based method.

*Reverse Transcription*

First‑strand cDNA was synthesized from the extracted RNA using the HiScript 1st Strand cDNA Synthesis Kit (Vazyme) according to the manufacturer’s instructions.

*Quantitative Real‑Time PCR (qPCR)*

qPCR was performed using AceQ qPCR SYBR Green Master Mix (without ROX) (Vazyme) on a CFX96 Real‑Time PCR System (Bio‑Rad) and a QuantStudio Real‑Time PCR System (Applied Biosystems). The reaction mixture and thermal cycling conditions are detailed in the Appendix. All primers were designed, synthesized, and validated prior to use. Relative gene expression was analyzed using the 2^–ΔΔCt^ method, with GAPDH as the internal reference.

Preparation of IAPP monomers

Recombinant Human Islet amyloid polypeptide (hIAPP) [Cusabio technology LLC., CSB-YP010931HU(A4)] and IAPP pentamutant (R44A/I59A/S62A/T69A/Y70F) peptides (Peptidebank, P225608) were resuspended at 2 mg/mL in pure hexafluoro-isopropanol (HFIP, Sigma) with shaking overnight at room temperature, lyophilized using an Alpha 1-2 LDplus freeze dryer (Christ, Osterode am Harz, Germany), and reconstituted in buffer to the desired concentration.

Aggregation assay and kinetic analysis

Spontaneous aggregation of 10μM hIAPP monomers in the absence and presence of 2μM Spike or Nucleocapsid proteins was assessed by monitoring amyloid fibril formation via the increase of fluorescence of the amyloid‐specific dye thioflavin-T (Sigma, T3516-5G) 96 hours. Lyophilized monomeric IAPP was reconstituted in 20 mM Tris-HCL (pH 6.8-7.4). Reconstituted peptides were mixed with ThioT (filtered at 0.22 µm) to a final concentration of 20 µM in 96 well microplate, PP, F-Bottom, chimney well (Greiner, REF655209, Germany), and amyloid formation was recorded on a LUX plate reader (ME Gemini EM) measuring fluorescence emission at 485 nm(excitation at 450 nm) every 10 min at 37℃ temperature (while shaking 20 secs before and between read). ThT fluorescence detected aggregation curves were made by

plotting the emission signals on y-axis vs the aggregation time on x-axis. All ThT curves are averages of at least three replicates and all experiments were performed at three independent times.

Transmission electron microscopy of fibril

For this experiment, 20 μl of each samples were added to 400-mesh carbon-coated nickel grids for 1 min and air-dried. Then, the samples on the nickel grids were stained with 1% (w/v) uranyl acetate for 1 min and air-dried. The specimens were observed under a JEM1400 transmission electron microscope (JEOL, Tokyo, Japan) at 80 kV.

Mass Spectrometry of IP samples

For MS analysis of co-IP, the Flag IP eluates (from IAPP+Spike, IAPP+N, and from control IPs) were run a short distance into an SDS-PAGE gel to concentrate proteins, then the entire lane was excised and subjected to in-gel trypsin digestion. Tryptic peptides were extracted and analyzed by LC-MS/MS on a Thermo Orbitrap instrument in DDA mode. MS/MS spectra were searched against a database containing SARS-CoV-2 protein sequences. Peptide hits for SARS-CoV-2 Spike and N with high confidence (Mascot score >50, FDR <1%) were considered valid. We identified 29 unique peptides from Spike (covering ~28% of Spike’s sequence) and 20 unique peptides from N (covering ~56% of N sequence) in the IAPP IP from co-transfected cells, whereas none were found in control IPs. This unambiguously confirmed the presence of Spike and N in the IAPP complexes.

MIN6 Functional Assays

For functional assays, MIN6 cells were plated in 24-well plates (~10^5^ cells/well) for secretion assays or 96-well plates for viability assays. The following conditions were tested in parallel: (1) untransfected control, (2) transfected with hIAPP-Flag alone, (3) transfected with Spike plasmid alone, (4) transfected with N plasmid alone, (5) co-transfected IAPP + Spike, (6) co-transfected IAPP + N. Each condition was done in n=3 wells for secretion and n=4 wells for viability.

*Cell viability* 48 h after transfection, CCK-8 reagent was added to 96-well cultures, and absorbance at 450 nm measured after 2 h to assess metabolic activity (proportional to viable cell number). LDH release was measured in culture supernatants using a colorimetric Cytotoxicity Assay Kit , with Triton X-100-lysed cells as 100% lysis control.

*Insulin secretion assay* After 48 h, MIN6 cells in 24-well plates were pre-incubated in Krebs-Ringer bicarbonate buffer (KRB) with 2.8 mM glucose for 30min. Then cells were stimulated with KRB containing 16.7 mM glucose for 1 hour (GSIS). The buffer was then replaced with 30 mM KCl in KRB (with 2.8 mM glucose) for 20 min (to trigger depolarization-induced insulin release). Insulin released into the buffer was collected, and cells were extracted with acid-ethanol (0.18 M HCl in 70% ethanol) to measure intracellular insulin content. Insulin was quantified by mouse insulin ELISA. Secretion was expressed as a percentage of content or as absolute concentration normalized to total protein. Data were averaged over 3 independent experiments.

*Cytokine measurement* After the secretion assay, culture media were also assayed for IL-6 and TNF-α using ELISA kits (Beyotime) according to manufacturer instructions, results normalized to cell protein.

Exosome isolation and analysis

For exosome studies, MIN6 cells were cultured in exosome-depleted FBS media and treated for 48 h with different vectors. Conditioned media (15 mL per condition) were collected and centrifuged at 300 ×g (to remove cells) and 10,000 ×g (to remove debris) then ultracentrifuged at 100,000 ×g for 70 min. Exosome pellets were washed in PBS and centrifuged again. The final pellet was resuspended in PBS. Nanoparticle tracking analysis (NTA) was performed using a NanoSight NS300 (Malvern) to determine particle size distribution and concentration. Median particle diameter and total particle count (particles/mL) were recorded. For insulin in exosomes, an aliquot of exosome prep was lysed with RIPA buffer and insulin content measured by ELISA.

Proximity ligation assay (PLA)

Isolated mouse islets were digested into single cells using trypsin-EDTA. The dissociated cells were then collected by centrifugation, resuspended, and fixed in 4% PFA. To detect in situ protein interactions, Duolink PLA kits (Sigma) were used. Fresh-frozen sections from β cells of FVB-hIAPP mouse islets treated with viral proteins were fixed with 4% PFA and permeabilized. Sections were incubated with primary antibody pairs: mouse anti-IAPP and rabbit anti-Spike S1 (or rabbit anti-N). Species-specific PLA probes (PLUS and MINUS oligonucleotide-conjugated secondary antibodies) were applied. Ligation and amplification steps were performed according to manufacturer’s protocol. PLA signals (puncta) indicate proximity <40 nm of the target proteins. Images were captured by confocal microscopy. The number of PLA puncta per islet was quantified for treated vs control islets.

Insulin secretion from islets

Batches of 10 islets were pre-incubated in low-glucose (2.8 mM) Krebs buffer for 30 min, then stimulated with high glucose (16.7 mM) for 1 h. The buffer was then replaced with 30 mM KCl in KRB (with 2.8 mM glucose) for 20 min (to trigger depolarization-induced insulin release).Insulin in the supernatant and islet insulin content (after acid-ethanol extraction) were measured by mouse insulin ELISA (which cross-reacts with rodent insulin; human IAPP presence does not interfere with the assay). Data were normalizedper total protein.

Cytokine secretion

After incubations, culture medium from islet wells was collected and IL-6/TNF-α levels measured by ELISA. Data were normalizedper total protein.

Proteomic analysis of islets

Treated and control islets were lysed in SDS sample buffer. Proteins were run ~5 mm into an SDS-PAGE gel and in-gel digested for shotgun proteomics (since yield was low, we pooled islets from 4 mice per condition). A label-free quantification using MaxQuant was performed. Due to limited material, only the most abundant changes were considered reliable (we observed decreases in F10, VAMP4 and increases in complement C3, qualitatively similar to NHP data, although not all passed statistical cutoff given sample pooling). These guided our targeted validation via immunostaining in NHP tissues.

Laser-capture microdissection (LCM) and spatial proteomics Workflow

*Laser-capture microdissection Preparation*

Formalin-fixed paraffin-embedded (FFPE) tissue sections were mounted onto Leica PET membrane slides. Hematoxylin and eosin (H&E) staining was performed prior to microdissection. Briefly, tissue sections were deparaffinized twice in xylene for 10 min each, followed by rehydration through a graded ethanol series (100%, 90%, 70%, and 50%) for 5 min each. Sections were subsequently rinsed in running water for 10 min, stained with hematoxylin for 3 min, differentiated in 1% hydrochloric acid ethanol for 1 min, counterstained with eosin for 1 min, and washed with running water for 5 min.

Regions of interest (ROIs) were selected according to histopathological characteristics. Laser capture microdissection was performed using the Leica Laser Microdissection System with Leica Laser Microdissection V8.2 software. Tissue sections were scanned under a 5× objective lens, and target regions were outlined manually using the Draw and Cut function. The cutting area was set to 0.1 mm². Laser parameters were configured as follows: energy=45; aperture width= 6; speed=3; specimen balance=17; offset=100%; pulse frequency=364. Microdissected samples were directly collected into low-binding collection tubes without sample transfer.

*Protein Extraction and Trypsin Digestion*

Microdissected tissue samples were lysed in 30 μL of 25 mM ammonium bicarbonate and incubated at 95℃ for 1 h. After cooling to room temperature, 2.5 μL trypsin solution (20 ng/μL; Promega) was added for enzymatic digestion. The digestion system was adjusted to weak alkaline conditions (approximately pH 8.0) and incubated at 37℃ for 4 h. Digestion was terminated by adding 1 μL of 10% formic acid to achieve a final pH of approximately 3.0.

Peptide samples were concentrated to approximately 15 μL using a vacuum concentrator, centrifuged at 20,000 × g, and the supernatant was transferred into autosampler vials for LC-MS/MS analysis.

*NanoLC Separation*

Peptide separation was performed on a nanoElute nanoLC system (Bruker, Germany). Peptides were first loaded onto a PepMap Neo Nano C18 trap column (300 μm × 5 mm, 5 μm particle size, 100 Å; Thermo Fisher Scientific) and subsequently separated on a self-packed analytical C18 column (150 μm × 35 cm, 1.8 μm particle size, 100 Å). Mobile phase A consisted of 0.1% formic acid in water, while mobile phase B consisted of 0.1% formic acid in acetonitrile. Peptides were eluted using the following gradient: 5-10% B for -3 min, 10-30% B for 3-43 min, 30-40% B for 43-53 min, and 40-100% B for 53-65min.

*Mass Spectrometry Acquisition*

Eluted peptides were analyzed using a timsTOF Pro 2 mass spectrometer (Bruker, Germany) operated in data-dependent acquisition parallel accumulation–serial fragmentation (ddaPASEF) mode.

For each acquisition cycle, one MS1 scan followed by four PASEF MS/MS scans was acquired. Ion accumulation and ramp times in the dual TIMS analyzer were both set to 100 ms. The ion mobility range was set from 1/K0=1.35 to 0.75 Vs cm^-2^.

Precursor ions were isolated using 2 Th windows for m/z < 700 and 3 Th windows for m/z > 700 across the mass range of m/z 100-1700. Collision energy was linearly ramped from 59 eV at 1/K0=1.6 Vs cm^-2^ to 20 eV at 1/K0=0.6 Vs cm^-2^. Precursors with intensities above 1,000 a.u. were selected for fragmentation until reaching a target intensity of 20,000 a.u., with a dynamic exclusion duration of 30 s.

*Protein Identification and Bioinformatics Analysis*

Raw MS data were processed using Spectronaut 17 against the UniProt database for peptide and protein identification. The minimum peptide length was set to seven amino acids, and up to two missed cleavages were allowed for tryptic digestion. N-terminal acetylation and methionine oxidation were set as variable modifications. Carbamidomethylation of cysteine was not set as a fixed modification because reduction and alkylation procedures were not performed during sample preparation.

The false discovery rate (FDR) for peptide-spectrum matches and protein identification was controlled below 1%.

Protein abundance values were log2 transformed, and missing values were imputed using half of the global minimum intensity. Differentially expressed proteins were identified using one-way ANOVA analysis with thresholds of p < 0.05 and |log2 fold change| ≥ 1.

Supplementary Fig. 1 Ultrastructural characteristics of primate islets in elder controls.

(a) TEM of β-cell from EC animal showing intact mitochondria, and normal ER. Scale bar, 2 µm.

(b) Baseline TEM image of EC β-cell for reference. Scale bar, 2 µm.

(c) Quantification of P-MLKL^+^ and P-RIP3^+^  positive cells without overlap with IAPP per field (20× magnification; n=10 images examined in total / group). Ten images from 3-4 animals per group were quantified. Measurements from the same animal were averaged, and animal-level means were used for statistical testing. Data are presented as the mean ± SD. p values were calculated by unpaired two-tailed Student’s t test: ***p < 0.01; ***p < 0.001*; ns, not significant.

(d) qPCR confirms expression of Flag-IAPP in transfected PANC-1 cells (n = 3, mean ± SEM, ***p < 0.01*).

(e) Confocal microscopy of co-transfected PANC-1 cells showing co-localization of Spike or Nucleoprotein (green) with IAPP (red). DAPI (blue). Scale bar, 5 µm.

(f) Internalization of Spike and N-CTD proteins into MIN6 β cells. MIN6 β cells were incubated with Spike-His or N-CTD-His at 37 °C for 24 h. Cells were then fixed and subjected to immunofluorescence staining with anti‑His antibody (red) to detect the internalized proteins, and anti‑ATPase antibody (green) as a plasma membrane marker. Nuclei were counterstained with DAPI (blue). Brightfield images show the cellular morphology and confirm the intracellular localization of the His‑tagged proteins (f1 and f2). Scale bar: 100 μm. Representative images from three independent experiments are shown.

Supplementary Fig. 2 Raw images of Western blot for co-immunoprecipitation in Figure 4. and ITM2B differentially recognizes free IAPP and viral protein-IAPP preformed complexes in SPR assays.

(a) The Western blot confirming successful co-expression of target proteins in HEK293T cells.

(b) Coomassie gel of immunoprecipitated complexes: Coomassie blue staining of the IP samples confirms successful pull-down and absence of bands in IgG control. Visible bands correspond to IAPP and co-precipitated proteins. No protein bands in the IgG-only IP lane indicate specificity.

(c) IP assays show specific interaction between Flag-IAPP and either Spike or Nucleoprotein. IgG control and empty vector controls validate specificity.

(d) Representative SPR sensorgrams and kinetic fitting curves for IAPP binding to immobilized recombinant ITM2B, showing high-affinity interaction (KD = 2.75E-09M).

(e) Representative SPR sensorgrams for recombinant ITM2B binding to the preformed Spike–IAPP complex, showing markedly weakened residual interaction (KD = 7.96E-05M).

(f) Representative SPR sensorgrams for recombinant ITM2B binding to the preformed Nucleocapsid-IAPP complex, showing no measurable binding under the same assay conditions.

Supplementary Fig. 3 Spatial proteomics reveals significant changes in differentially expressed proteins from pancreatic islets.

(a) Volcano plot visualizing significantly upregulated (red) and downregulated (blue) proteins in EM islets compared to EC islets. Thresholds are set at adjusted *p-value <0.05* and |log₂FoldChange| >1.

(b) Hierarchical clustering heatmap representing the relative abundance of significantly dysregulated proteins (rows) across individual samples (columns), clearly differentiating infected (EM) from uninfected elder control (EC) groups. Color intensity corresponds to normalized protein abundance (red, increased; blue, decreased).

(c) Venn diagram of the intersection between differentially expressed proteins in primary islet model proteomics and spatial proteomics, showing two proteins exhibiting consistent directional changes (downregulated F10 and upregulated C3).

(d) Cross-study APOE/STRING/KEGG contextualization panel highlighting APOE as a convergent upregulated factor across PIGs-associated literature and the spatial islet proteome.

**Supplementary Fig. 4 KEGG pathway visualization of complement and coagulation cascades.**

(a) KEGG pathway visualization highlighting significantly dysregulated proteins involved in the complement and coagulation cascades in elder model (EM) pancreatic islets. Color intensity indicates fold-change of protein expression (red: increased; green: decreased).

**Supplementary Fig. 5 Endoplasmic reticulum (ER) stress and inflammatory responses in SARS-CoV-2-infected islets.**

(a) Immunofluorescence assessment of ER stress and inflammatory markers (CHOP, green; GRP78, orange; IL-6, red; TNF-α, yellow) alongside insulin (cyan) and IAPP (white) reveals increased ER stress and inflammatory cytokine expression specifically in EM islets. Insets show magnified views highlighting co-localization of ER stress markers with inflammatory cytokines and IAPP aggregates. Low-magnification image with scale bar, 800 μm. Insets show enlarged views, Scale bars, 50 µm.

(b) Quantification of immunofluorescence staining for ER stress markers CHOP and GRP78, and inflammatory cytokines IL-6 and TNF-α, expressed as percentage of positive cells per islet field (5×magnification). Elder model (EM) macaques display significantly increased expression levels of these markers compared with adult control (AC) and elder control (EC) groups.

(c) Co-localization analysis showing the percentages of cells double-positive for IAPP and CHOP, GRP78, IL-6, or TNF-α, indicating enhanced ER stress and inflammatory responses specifically associated with IAPP-positive cells in infected elder macaques.

Statistical analysis: Ten fields/images from 3-4 animals per group were quantified. Measurements from the same animal were averaged, and animal-level means were used for statistical testing. Data are presented as the mean ± SD. p values were calculated by unpaired two-tailed Student’s *t* test: ***p < 0.01*; ****p < 0.001*; ns, not significant.

(d, e) IL-6 and TNF-α levels in media from MIN6 in vitro model acute expression of spike or nucleocapsid-or cognate peptides. Both cytokines are elevated in Spike+ IAPP and N-CTD + IAPP treated Min6 model relative to alone group or control group.

(f, g) IL-6 and TNF-α levels in media from primay isolated islets ex vivo model acute expression of spike or nucleocapsid-or cognate peptides. Both cytokines are elevated in Spike+ IAPP and N-CTD+IAPP relative to alone group or control group. N=3, multiple comparisons were evaluated by two-sided Welch’s t-tests with Holm correction within each family of tests (vs control).

(h) Quantification of vesicular diameter in the region of trapped vesicles. (n=14 vesicles).

(i) qPCR analyses of human and mouse IAPP mRNAs. mRNA levels were quantified and normalized to GAPDH. mRNA expression data are presented relative to the FVB control group. Data are shown as mean ± SD from three independent experiments (n = 3). Statistical significance was determined using unpaired two-tailed Student’s t-test: **p < 0.05, **p < 0.01, ***p < 0.001*; ns, not significant.

**Supplementary Fig. 6 KEGG pathway visualization of SNARE interactions in vesicular transport.**

(a) KEGG analysis shows downregulation of SNARE pathway components including VAMP4/7/8 in EM islets. Protein expression changes are indicated by color intensity (green: downregulated; red: upregulated).

**Supplementary Fig. 7 Full-length blots are provided to demonstrate the integrity of the data, with the cropped regions in main text used for quantification clearly marked.** Membranes were probed with anti-target protein antibody and anti-β-actin/GAPDH antibody as a loading control. The positions of the molecular weight markers (in kDa) are indicated on the left of each blot. Arrows denote the specific bands corresponding to target protein.

**Supplementary Table 1 Confidence metrics for AlphaFold3 molecular docking predictions**

| **Bait protein** | **Bait protein predicted Template Modeling score (pTM)** | **Genomic protein ID** | **interface predicted Template Modeling score (ipTM)** | **pTM** | **ipTM + pTM** |
| --- | --- | --- | --- | --- | --- |
| IAPP | 0.25 | QOS45029.1 | 0.22 | 0.71 | 0.93 |
| IAPP | 0.25 | P0DTC9 | 0.17 | 0.32 | 0.49 |

**Supplementary Table 2 Primers used for quantitative real-time PCR.**

| **Gene** | **Forward sequence (5'-3')** | **Reverse sequence (5'-3')** | **PCR Products(bp)** |
| --- | --- | --- | --- |
| GAPDH | TGGAGCCAAAAGGGTCATCA | ATGAGCCCTTCCACAATGCC | 183 |
| IAPP-homo | TCTCATCTACCAACGTGGGATC | GGGCAAGTAATTCAGTGGCTC | 86 |
| Iapp-mus | TGGCACTGAACCACTTGAGA | GTTGTTGCTGGAACGAACCAA | 131 |
| Ins1 | GGACTATAAAGCTGGTGGGCA | AGGTGGGGACCACAAAGATG | 195 |
| Ins2 | GCAGAAGCGTGGCATTGTAG | TTCATTGCAGAGGGGTAGGC | 111 |
| Vamp4 | AAATCAGGTCACCACTGCCG | TCACAAACTGCTGCAAGGGA | 83 |
| Vamp8 | CCAGAATGTGGAGCGGATCT | CCTTCTGGGACGTTGTCTTGA | 110 |
| Rab5a | TACTACCGAGGAGCACAAGC | GGACTTGCTTGCCTTTGAAGT | 107 |
| Vps35 | ATCAGCGGATTCGCTTCACA | AGCACTGATAGTCTGGTGAGC | 146 |
| Stx2 | TTTCACAACAGTGGCCTTGC | CGAAGTCTCTTCGTCAGCACT | 151 |
| Pdx1 | AAGAGGACCCGTACTGCCTA | TTCCACTTCATGCGACGGTT | 167 |
| Stx1a | GTGGCCAGAGTTGCATGTTG | TGGCACATCAATGGGAGACG | 141 |
| Stxbp1 | TCACCAACATGGCTCACCTC | TGAGAGCTGGTAGGTCTGCT | 110 |
| Mafa | CAAGGAGGAGGTCATCCGAC | CTCTCCAGAATGTGCCGCTG | 108 |
| Nkx6-1 | AGACCCACGTTCTCTGGACA | AATAGGCCAAGCGTGCTCTC | 97 |
| Ucn3 | GGAAGATGTGCCCTTGCTGA | GCTGTGCCTGGGATTGGTAT | 173 |
| Slc2a2 | ATTCTTTGGTGGGTGGCTCG | CAGCAATGATGAGGGCGTGT | 134 |
| Pcsk1 | AGGTCAACCCCACACTTTCC | CATGAGTTGTCTGGGGCTCT | 84 |

**Supplementary Table 3 Spatial proteomics maps a conserved APOE-driven PIGs overlap and raw size distribution and concentration profiles of exosomes had uploaded in Supplementary Material**
